# Analysis of the Codon Usage Pattern of HA and NA Genes of H7N9 Influenza A Virus

**DOI:** 10.3390/ijms21197129

**Published:** 2020-09-27

**Authors:** Jiumeng Sun, Wen Zhao, Ruyi Wang, Wenyan Zhang, Gairu Li, Meng Lu, Yuekun Shao, Yichen Yang, Ningning Wang, Qi Gao, Shuo Su

**Affiliations:** College of Veterinary Medicine, Nanjing Agricultural University, Nanjing 210095, China; 2019207028@njau.edu.cn (J.S.); 2017207031@njau.edu.cn (W.Z.); 2017107069@njau.edu.cn (R.W.); wenyan__zhang@163.com (W.Z.); ligru2018@163.com (G.L.); LM98981128@163.com (M.L.); chrissyk@163.com (Y.S.); yichenyang0708@163.com (Y.Y.); 17416104@njau.edu.cn (N.W.); gaoqi@njau.edu.cn (Q.G.)

**Keywords:** H7N9, codon usage bias, HA gene, NA gene, host specificity

## Abstract

Novel H7N9 influenza virus transmitted from birds to human and, since March 2013, it has caused five epidemic waves in China. Although the evolution of H7N9 viruses has been investigated, the evolutionary changes associated with codon usage are still unclear. Herein, the codon usage pattern of two surface glycoproteins, hemagglutinin (HA) and neuraminidase (NA), was studied to understand the evolutionary changes in relation to host, epidemic wave, and pathogenicity. Both genes displayed a low codon usage bias, with HA higher than NA. The codon usage was driven by mutation pressure and natural selection, although the main contributing factor was natural selection. Additionally, the codon adaptation index (CAI) and deoptimization (RCDI) illustrated the strong adaptability of H7N9 to *Gallus gallus*. Similarity index (SiD) analysis showed that *Homo sapiens* posed a stronger selection pressure than *Gallus gallus*. Thus, we assume that this may be related to the gradual adaptability of the virus to human. In addition, the host strong selection pressure was validated based on CpG dinucleotide content. In conclusion, this study analyzed the usage of codons of two genes of H7N9 and expanded our understanding of H7N9 host specificity. This aids into the development of control measures against H7N9 influenza virus.

## 1. Introduction

Before the outbreak of H7N9 in China in 2013, H7 subtype avian influenza viruses (AIVs) mainly existed in birds and with less frequency in humans leading to mild symptoms [1]. After March 2013, H7N9 was first isolated in human [2,3,4] and so far, five epidemic waves have been well studied and defined from October of each year to September of the following year [5]. By the end of the fifth wave, H7N9 caused 1564 human cases with a mortality rate of nearly 40% according to the World Health Organization (WHO). The number of infections of the first four waves decreased almost year by year, while the number of cases in the fifth wave increased sharply from late 2016 to early 2017, up to 766 cases (http://www.who.int/influenza/human_animal_interface/HAI_Risk_Assessment/en/), with the simultaneous emergence of highly pathogenic H7N9, indicating a serious threat to public health. The main route of human H7N9 infection is transmission from poultry [6,7]. According to the WHO, most human clinical cases had been exposed to live birds, in particular, live poultry markets [6,8] which may facilitate the adaptability and transmission from birds to humans.

H7N9 AIV belongs to the influenza a virus genus of *Orthomyxoviridae*. It is a single segmented negative-stranded RNA enveloped virus. The genome contains eight fragments of a total length of approximately 13,000 bases. The genome encodes the hemagglutinin (HA), neuraminidase (NA), matrix (M1, M2), RNA polymerase (PB1, PB2, PA), nucleoprotein (NP) proteins, and the NS1 and NS2 non-structural proteins. It was reported that H7N9 influenza virus originated from gene reassortment [4,9]. The surface of the virus is H7 and N9 probably derived from migratory birds, while the internal six gene fragments derived from another avian influenza virus, H9N2 [10]. Gene reassortment is an important mechanism of influenza virus evolution. Mutation and recombination also drive viral evolution, another evolution mechanism in other RNA and DNA viruses [11,12,13,14,15,16,17]. HA and NA are two major envelope proteins. In highly pathogenic AIV, the HA cleavage site contains at least four basic amino acids allowing distinction between high and low pathogenicity strains [18]. HA is responsible for the attachment to sialic acid receptors and entry into host cells, and is the main antigen determinant of host-induced immune response [19]. NA acts as an enzyme to release sialic acid to aid the release of the virus [20]. These two proteins determine the subtype and can be used as targets for antiviral drugs.

Amino acids are coded in the form of triplet codons. An amino acid can be encoded by one or more (no more than six) triplet codons. Codons encoding the same amino acid are called synonymous codons. The preferential use of a particular codon is called codon bias [21]. Factors that can influence codon usage bias include natural selection, mutation pressure, structure and properties of proteins, tRNA abundance, and nucleotide composition shape [22,23,24]. The codon usage pattern varies among viruses, and the codon usage analysis has been widely used to reveal virus genetic evolution, host adaptation. The codon usage patterns of Zika, Henipa, and Equine influenza viruses are more driven by natural selection and are host-specific, while in Rubella virus the codon usage pattern is dominated by mutation pressure [25,26,27,28]. A similar codon usage pattern between virus and host will severely impede host translation. In addition, codon bias also affects protein function and translation efficiency [29,30]. Therefore, the analysis of H7N9 codon usage pattern can contribute to understanding the host adaptation and virus evolution, providing valuable information for vaccine design strategies. A previous study showed a low codon usage of the H7N9 PB2 gene [31]. Since HA and NA play key roles in attachment to the host, pathogenicity, and progeny production, we performed a comprehensive analysis of the codon usage pattern and phylogenetic analysis of these two genes in China to better understand the evolutionary changes of H7N9.

## 2. Results

### 2.1. Phylogenetic Analyses of the HA and NA Genes of H7N9

**Figure 1:**
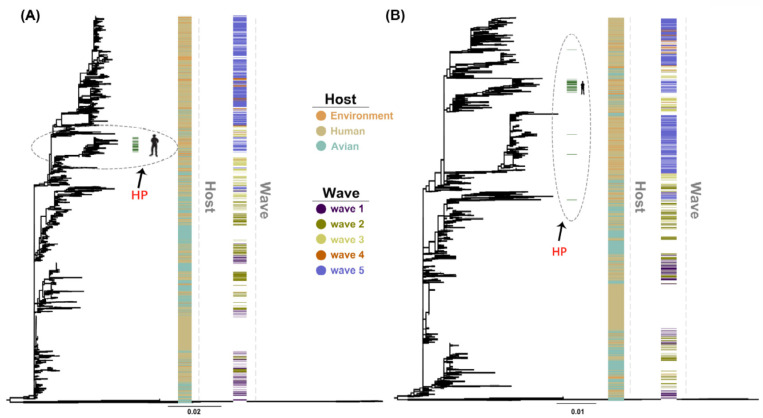
Maximum likelihood (ML) trees of H7N9 hemagglutinin (HA) (**A**) and neuraminidase (NA) (**B**) genes were reconstructed using RAxML (v8.2.10) with 1000 replications. The environment, human, and avian are represented in orange, beige, and cyan, respectively. Dark purple, olive green, light yellow, orange, and light purple correspond to waves 1 to 5. Grass green corresponds to high pathogenicity. HP: high pathogenicity.

The maximum likelihood (ML) trees of HA and NA genes showed similar topology to previous studies [5] with HA highly pathogenic sequences of human clustering in almost a single branch and dispersion of highly pathogenic sequences in the NA gene (Figure 1). Furthermore, we found a closer relationship of human with chicken compared with duck, with branches containing most duck-derived strains far from other strains. In addition, the newly added strains in 2018 clustered with the fifth wave.

### 2.2. Trends of Codon Usage Patterns Based on Different Classifications of HA and NA

**Figure 2:**
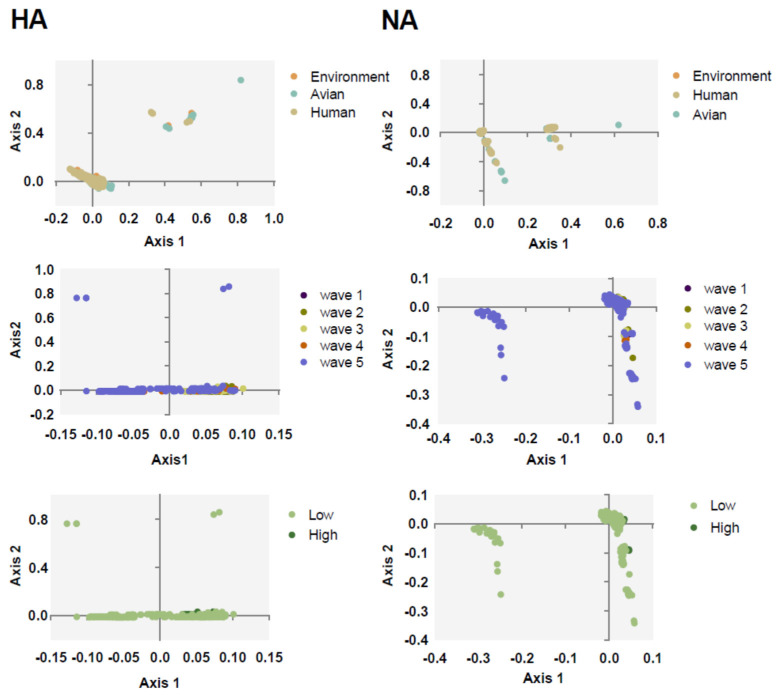
PCA taxonomic analysis of HA (**left column**) and NA (**right column**). The environment, human, and avian are represented in orange, beige, and cyan, respectively. Circles are marked by dark purple, olive green, light yellow, orange, and light purple, corresponding to waves 1 to 5, respectively. Grass green corresponds to high pathogenicity.

To understand the major variations of H7N9 HA and NA, PCA was calculated according to the relative synonymous codon usage (RSCU) value. We found that the first and second axis account for 29.81% and 27.95% variations of HA, respectively, and 27.62% and 21.76% for NA. Next, we classified all the sequences in clusters. Strains categorized based on the environment, avian, and human clustered together with no effective separation among them, except for several avian strains for both NA and HA (Figure 2). This is consistent with the evolutionary tree suggesting that they may derive from the same source. We also found that, apart from the strains of fifth wave and low pathogenicity strains, other strains often clustered together.

### 2.3. Nucleotide Composition

We found that the highest mean mononucleotide composition of H7N9 HA and NA corresponded to nucleotide A (greater than 34%), while the remaining nucleotide values were approximately 20%. The frequency of nucleotide on the third position suggested that, in synonymous codons, A was also the highest in accordance with the mononucleotide composition. The overall AU content accounted for 60% compared with 40% of GC. For HA and NA, the highest value of the position of GC was for position 1 and the lowest value for position 3 (Table 1). The same patters were identified when considering different classifications, different waves, hosts, and pathogenicity. Altogether, we can conclude that these two genes are biased towards the use of A bases and thus, the existence of codon usage preferences in the HA and NA genes.

### 2.4. Lower Codon Usage Bias in HA and NA Gene

**Figure 3:**
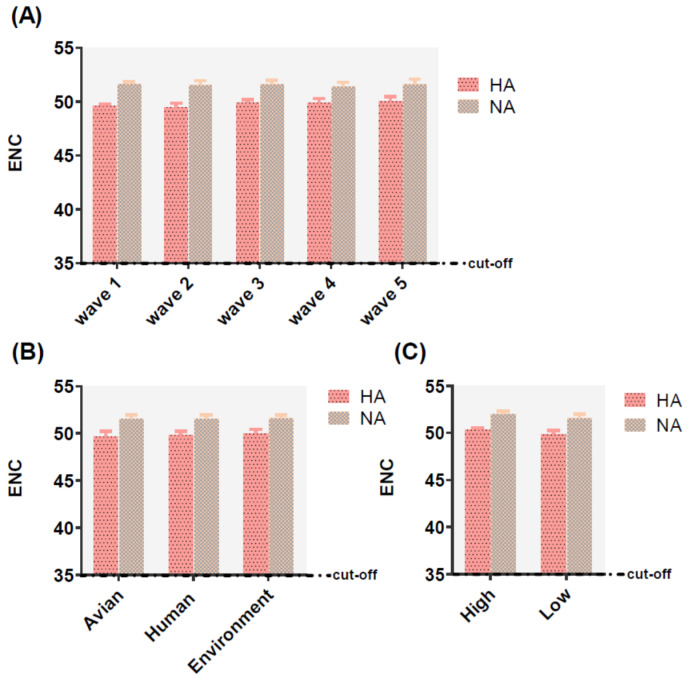
Effective number of codon (ENC) analysis of HA (displayed in pink and black dot histogram) and NA (displayed in earthy yellow and gray squares histogram) of different waves (**A**), hosts (**B**), and pathogenicity (**C**). The cut-off value of the ENC value is 35. The larger the ENC value, the lower the codon usage bias.

The effective number of codons (ENC) values ranged from 48.49 to 53.46 for HA and 49.36 to 53.02 for NA, which the ENC value higher 35 represented a lower codon usage. In addition, the mean values of HA and NA were 49.78 ± 0.476, 51.54 ± 0.404, respectively. The same phenomenon was also identified in different classifications including, waves, hosts, and pathogenicity (Table S2), with values greater than 35, indicative of these two genes possessing a lower preference of codon usage no matter in relating to waves, hosts, or pathogenicity (Figure 3).

### 2.5. RSCU Value of HA and NA Genes

Based on RSCU analysis (Table 2), we found that optimal codons terminated in nucleotide A (11 codons in A, 5 in U, and 1 in G and C) for HA. For NA, nucleotide A was also the most commonly used base at the end of the optimal codon, followed by C, U, and G. It is worth noting that 7 and 8 for HA and NA genes of the 18 preferred synonymous codons had a value > 1.6, the highest being AGA (3.4 for *HA* and 2.47 for *NA*), which indicates they are over-represented. Moreover, almost all of the above synonymous codons were A-ended, whereas most of synonymous codons were underrepresented. Most of the low-expression synonymous codons ended in C, G, and U, with the exception of UUA and CGA, which encode Leu and Arg in HA. In addition, the codon usage results based on different categories were closely related to the results of all sequence analyses. Furthermore, we evaluated the H7N9 RSCU value compared with the host species, even though the association was almost non-existent. In particular, for the optimal synonymous codon only 3 to 5 of the 18 preferred codons were identical. The relevance was considered to be minimal. The RSCU of highly pathogenic HA and NA were identical with the host, rather than with all sequences. 

### 2.6. Factors Driving Codon Usage Bias

**Figure 4:**
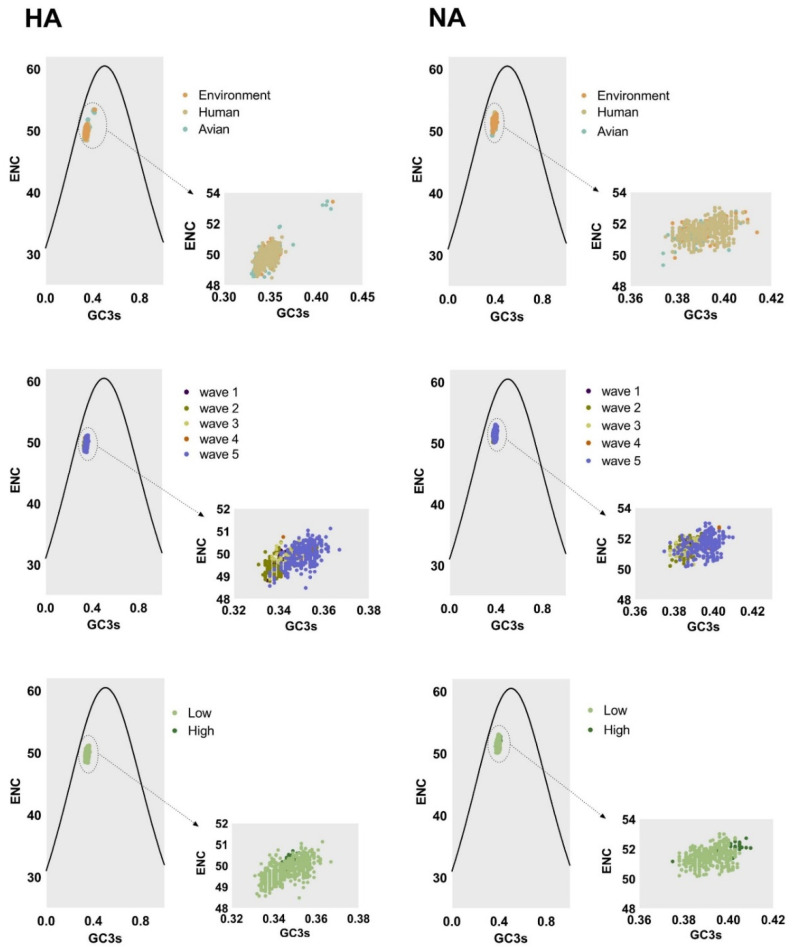
Left column and right column of ENC-plot analysis represent the HA and NA genes, respectively. The environment, human, and avian are represented in orange, beige, and cyan, respectively. Dark purple, olive green, light yellow, orange, and light purple correspond to waves 1 to 5, respectively. Grass green corresponds to high pathogenicity.

**Figure 5:**
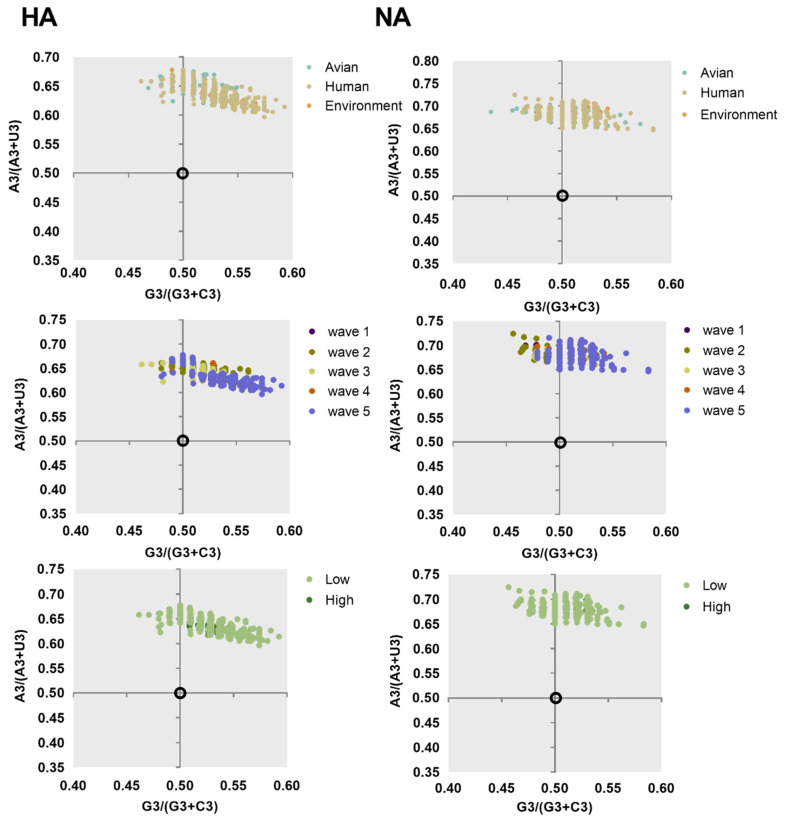
Parity Rule 2 (PR2) analysis of HA and NA of different classification. Far away from the origin indicates that there is a bias between the effect of mutation pressure and natural selection.

Factors shaping the codon usage bias of H7N9 HA and NA genes were illustrated by ENC-plot, Parity Rule 2 (PR2), and neutrality analysis. We found that the points corresponding to HA and NA genes clustered below the expected curve regardless of the classifications in ENC-plot (Figure 4). This indicates the effect of mutational pressure on codon usage bias with natural selection being more important than other factors. Based on PR2 analysis (Figure 5), these points were away from the origin (0.5, 0.5), indicating a bias between the effect of mutation pressure and natural selection.

**Figure 6:**
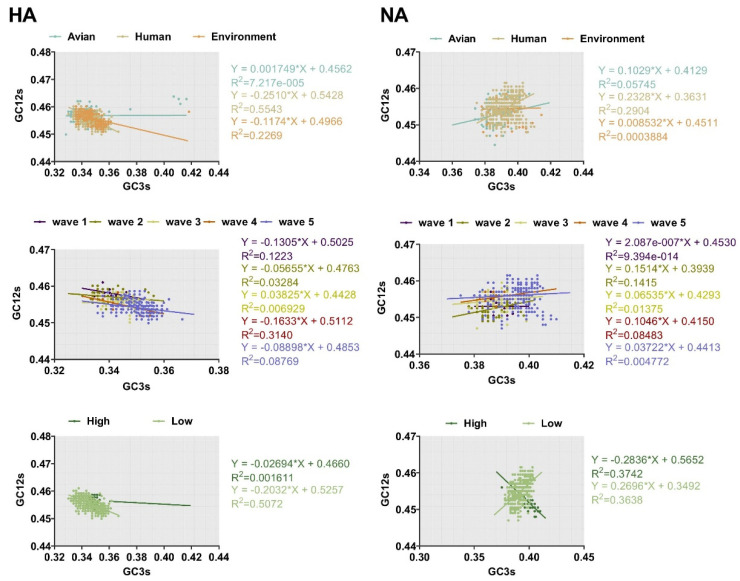
Neutrality analysis of HA and NA depicted by plotting GC3s against GC12s. The higher the slope, the greater the effect of natural selection pressure. The environment, human, and avian are represented in orange, beige, and cyan, respectively. Dark purple, olive green, light yellow, orange, and light purple correspond to waves 1 to 5, respectively. Grass green corresponds to high pathogenicity.

The ENC-plot analysis and PR2 analysis showed that both mutation pressure and natural selection govern the codon usage pattern. Next, to assess the extent of the mutational pressure compared with natural selection, the correlation between GC12s and GC3s was investigated by neutrality analysis. For both HA and NA, the correlation between the indexes was extremely significant (*p* < 0.0001). However, the coefficient of the slope was 0.001749 ± 0.4562, −0.2510 ± 0.5428, and −0.1174 ± 0.4966 for avian, human, and the environment of HA, respectively. This indicates that the contribution of natural selection was 99%, 75%, and 89%, respectively. We also found strong natural selection pressure in sequence analysis according to other classifications, with the regression slope close to 0.0 (Figure 6). In general, although natural selection pressure had different strength for different classifications, the influence of natural selection was more dominant than mutation pressure in shaping codon usage bias of HA and NA complete sequences.

### 2.7. The HA and NA Genes of H7N9 Virus Are Highly Adapted to Gallus gallus

**Figure 7:**
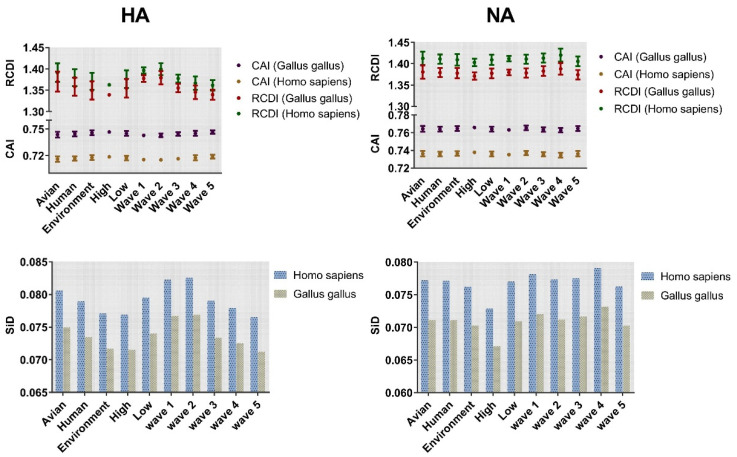
Codon adaptation index (CAI) and relative codon deoptimization index (RCDI) analysis of HA and NA. The coordinate axis was divided into two segments, and then placed the above two analyses on the same figure for observation. CAI corresponds to dark purple for avian and coffee for human. In RCDI, dark red and dark green represent *Gallus gallus* and *Homo sapiens*, respectively. Cylindrical maps are classified according to different taxonomy with SiD values as ordinates. Blue and yellow are used to represent *Homo sapiens* and *Gallus gallus*, respectively.

The adaptation of H7N9 HA and NA genes to *Gallus gallus* and *Homo sapiens* was investigated by codon adaptation index (CAI) analysis (Figure 7). Both the HA and NA genes showed higher CAI values in *Gallus gallus* compared with *Homo sapiens*. Among the three classifications, the highest values of the HA gene were environment, high pathogenicity, and wave 5 with mean ± SD values of 0.7457 ± 0.003, 0.7467 ± 0.001, and 0.747 ± 0.0019, respectively. Similar results were found for NA (Figure 7). Regarding the lowest CAI value, a different trend was identified for HA and NA. The lowest CAI value was found in the low pathogenicity classification irrespective of the gene.

Relative codon deoptimization index (RCDI) analysis was performed to explore the codon deoptimization. The value of *Homo sapiens* was higher than that of *Gallus gallus*. The RCDI value for environment (1.349 ± 0.021), high pathogenicity (1.339 ± 0.003), and wave 5 (1.340 ± 0.012) was lowest in HA. For NA, the lowest value was environment (1.378 ± 0.012), high pathogenicity (1.371 ± 0.008), and wave 5 (1.374 ± 0.011). Overall, the CAI or RCDI values of NA gene were higher than HA.

### 2.8. Strong Selection Pressure of Homo Sapiens on H7N9

**Figure 8:**
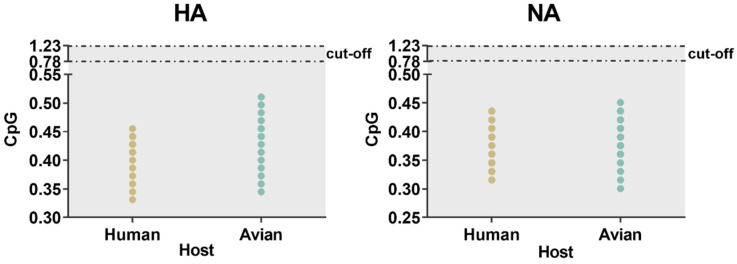
The ratio of CpG dinucleotide of strains of avian and human in HA and NA. When the relative dinucleotide abundances are <0.78, it indicates that dinucleotides are underrepresented. The color distribution is consistent with the previous figure.

Similarity index (SiD) analysis was performed to find out the effect of the overall codon usage pattern of the host on the total codon usage of the H7N9 virus. We found that *Homo sapiens* had a strong selection pressure on the virus compared with *Gallus gallus* (Figure 7). The codon similarity between host species and the varied waves, as well as pathogenicity in HA showed a gradual downward trend from wave 1 to 5 while low pathogenicity was higher than that of high pathogenicity. For NA, although there was no same downward trend as HA, wave 5 was significantly lower than the other waves, while the conclusion of pathogenicity was identical to that of HA. In general, *Homo sapiens* had a greater impact on H7N9. Furthermore, we also calculated the incidence of CpG dinucleotide frequencies to understand their relationship with the host. We tracked the evolution of all H7N9 strains, including the CpG content after cross-host (Figure 8). The range of CpG content of HA was 0.345 to 0.511 for *Gallus gallus* and 0.331 to 0.455 for *Homo sapiens*. The values of NA were 0.300 to 0.451 for *Gallus gallus* and 0.316 to 0.436 for *Homo sapiens*. All these values were lower than 0.78, implying that CpG was underrepresented.

## 3. Discussion

Influenza virus evolution is driven by genetic shift and drift [32]. H7N9 AIV originated from poultry via reassortment in 2013 and caused the highest number of human cases in the latest (fifth) wave according to the WHO. Therefore, it is urgent to analyze its genetic evolution and adaptability. Codon usage studies of the epidemic H7N9 virus in different avian hosts based on the PB2 gene have been reported [31]. These studies lay foundation for further research on the evolution of H7N9. Herein, we collected 2024 HA and 1989 NA genes sequences of all H7N9 available sequences in China from all hosts until 2019 and performed a comprehensive and systematic analysis based on host, wave, and pathogenicity.

Based on ML tree of HA and NA genes, H7N9 isolates from different waves and hosts displayed no clear dependent branch. Even if there were obvious sequence differences between exact genes of isolates with high and low pathogenicity, most of the high pathogenicity sequences clustered together. However, they shared the same branch with low pathogenicity isolates, indicating they derive from a common source as previously shown [33]. Based on codon analysis, the results of PCA were consistent with the evolutionary tree. Of note, the branch-clustering high pathogenicity strains displayed highest homology with chickens rather than other poultry animals.

Codon usage bias is common in other viruses, such as ZIKA virus [25], H3N2 CIVs [34], etc. We found that the overall AU content of HA and NA was higher than GC and the optimal codons ended with A. ENC values revealed a low-level overview among HA and NA. A higher codon usage bias is in contrast with other IAV, such as the 1918 pandemic H1N1 (52.50) [35], H3N8 EIVs (52.09) [36], and H5N1 influenza virus (almost 52.00) [37]. Moreover, the average value of the HA gene of ICV and IDV were 44.15 ± 0.92 [38], 48.3 ± 0.179 [39], respectively. It is hypothesized that a low codon bias of H7N9 AIV compared with other influenza viruse subtypes might promote effective replication by reducing competition between viruses and hosts during protein synthesis according to previous reports [40]. Hence, H7N9 had different extent of codon usage bias in the avian and human hosts with lower codon usage preference in the human than in avian host helping maintain the successful replication of the virus and possibly increase in virulence [40]. The nucleotide composition displayed an extremely higher AU content than GC, in agreement with the optimal synonymous codon on the third position. We concluded that the codon preference was impacted by composition, i.e., mutation pressure. In addition, we compared the RSCU of the virus with the host RSCU. H7N9 evolved almost exactly in the opposite direction to host RSCU. It has been reported that the usage of the same synonymous codon allows efficient translation of the virus [41]. Thus, the phenomenon observed here indicates that the translation efficiency may be reduced, while the viral protein can be correctly folded [41].

By ENC-plot and PR2 analyses, we found the effect of both mutation pressure and natural selection. However, the predominant factor in shaping the codon usage bias of specific classification was natural selection. In addition, CAI analysis was used to analyze the role of natural selection deeply. Overall, the adaption of H7N9 to *Gallus gallus* was higher than to *Homo sapiens*. However, on the basis of host classification, the CAI value of *Homo sapiens* was higher than that of *Gallus gallus*. In addition, the CAI values in *Homo sapiens* relating to waves showed a gradually increasing tendency. This may be related to the emergence of highly pathogenic strains in the fifth wave [42] leading to a large number of human deaths. The CAI of high pathogenic strains was also expected to be higher than that of low pathogenic. Therefore, we inferred that the level of CAI might be related to the virulence of the virus to host and potential hosts, similarly to previously reported data [43]. In addition, the combination of RCDI and CAI analysis further validated the high adaptability of the virus to *Gallus gallus*. For SiD analysis, the strong selection pressure on *Homo sapiens* compared with *Gallus gallus* is indicative of the virus gradually adapting to *Homo sapiens*, involving new mutations coinciding with huge outbreaks of human infections in the fifth wave in China [44]. The lower CpG content found in human, especially for HA indicates that there is a strong selection pressure in human [45].

In general, we found that H7N9 has a low codon bias and is mainly driven by natural selection. After avian influenza virus transmitted to human, a rapid adaptation was observed in relation to codon usage bias. This information is of great significance for studying the structure and function of H7N9 HA and NA and for understanding the evolution of H7N9. More and more epidemiological surveillance should be considered due to the increasing number of human infections and deaths caused by the emergence of high pathogenic viruses.

## 4. Material and Methods

### 4.1. Data Sequences

All the complete coding sequences of HA and NA gene of H7N9 virus (including viruses infecting avian and human) from China were downloaded from GenBank of National Center for Biotechnology Information (https://www.ncbi.nlm.nih.gov/genbank/) and GISAID (https://www.gisaid.org/). A total of 2024 HA and 1989 NA genes were analyzed. The detailed information of strain name, collection date, and province as well as host is listed in the Supplementary Materials (Table S1).

### 4.2. Phylogenetic Analysis

Sequences were aligned using MAFFT (v 7.1) [46], manually adjusted, and divided into three sets according to host, wave, and high or low pathogenicity to humans. Maximum likelihood trees of HA and NA genes were reconstructed with RAxML (v8.2.10) [47] using the GTR+I+Γ nucleotide substitution model, which was inferred by ModelGenerator [48].

### 4.3. Correspondence Analysis

Correspondence analysis is a method of multiple vector statistics that reveals the codon usage pattern trends of genes. Each sequence is presented in 59-dimensional result using the RSCU value as a benchmark. Previous studies showed that the first two axes account for a large proportion of the total changes, indicating that they account for the main part of codon usage change [49,50]. Therefore, we selected the first two dimensions of the data as the basis for the next analysis.

### 4.4. Codon Usage Bias Index

#### 4.4.1. Nucleotide Composition

(i) The base composition (A%, U%, G%, C% and AU, GC) were calculated using Bioedit v7.0.9.0. (ii) The different positions of GC in codons were calculated by the online cusp program (http://emboss.toulouse.inra.fr/cgi-bin/emboss/cusp). (iii) The composition of A3, U3, C3, and G3 were solved by codonW 1.4.2. Met and Trp amino acids are encoded by one codon while termination codons do not encode any amino acid; thus, there is no codon bias for these five codons and they were excluded from the analysis.

#### 4.4.2. Relative Synonymous Codon Usage Analysis

In order to understand the frequency at which codon is used in a synonymous codon family, the RSCU value was calculated by MEGA 7.0. The calculation formula of RSCU is as follows:

$$\mathrm{RSCU}=\frac{g_{ij}}{\sum_{j}^{n_{i}}g_{ij}}n_{i}$$

where the *g_ij_* is the quantity of the *i*th codon of jth amino acid. The denominator is the sum of all synonymous codons encoding the amino acid, and is multiplied by the number of synonymous codons at the end [51]. If the value = 1 means that the usage frequency of the synonymous codons is equal [52]. If it is >1.0 or <1.0, it means abundant codons and less abundant codons, respectively. Two extreme values of RSCU were >1.6 and <0.6 and were treated as ‘over-represented’ and ‘underrepresented’ codons, respectively [53].

#### 4.4.3. Effective Number of Codons Analysis

The effective number of codons is considered a standard method to evaluate codon usage bias [54]. The ENC values range from 20 to 61, representing the use of only one codon per amino acid and all possible synonymous codons. The formula to calculate it is as follows:

$$ENC=2+\frac{9}{\bar{F_{2}}}+\frac{1}{\bar{F_{3}}}+\frac{5}{\bar{F_{4}}}+\frac{3}{\bar{F_{6}}}$$

where $\bar{F_{i}}$(i = 2, 3, 4, 6) is the average of the *F_i_* values of the *i*-fold degenerate amino acids. Using the formula to calculate the *F_i_* value, we obtain:

$$\bar{F_{i}}=\frac{n\sum_{j=1}^{i}{\left(\frac{n_{j}}{n}\right)}^{2}-1}{n-1}$$

where *n* is the total number of codon occurrences of the amino acid and *n_j_* is the total number of occurrences of the *j*th codon of the amino acid. The cut-off point of the ENC value is 35 [55]. When it is less than 35, it means that the gene has a strong codon preference. The larger the ENC value, the lower the codon usage bias.

### 4.5. Factors Mediating Codon Usage Bias

#### 4.5.1. ENC-Plot Analysis

The main codon usage bias driving factors are mutation pressure and natural selection [56,57] among others such as, replication, protein structure, and dinucleotide frequency [36,58]. The ENC value is plotted in the ordinate and GC3s as the abscissa for analysis. The expected ENC value was calculated as follows:

$$ENC_{expected}=2+s+\frac{29}{s^{2}+{\left(1-s\right)}^{2}}$$

where ‘*s*’ is the frequency G + C at the third position of synonymous codons. If the point lies on or around the standard curve, it means codon usage bias is merely constrained by mutation pressure. In contrast, if the point lies below and away from the standard curve, this means other factors besides mutation pressure drive codon bias.

#### 4.5.2. Parity Rule 2 Analysis (PR2)

PR2 analysis takes [A3/(A3 + U3)] of four-codon amino acids as the ordinate and [G3/(G3 + C3)] as the abscissa and investigates the impact of mutation pressure and natural selection pressure. It takes 0.5 and 0.5 as the origin of coordinate axis. When the value is located at the origin, it is confirmed that there is no deviation between the effect of mutation pressure and natural selection [59,60].

#### 4.5.3. Neutrality Analysis

Neutrality analysis was used to verify the major factors effecting the codon usage pattern, especially mutation pressure or natural selection [61]. It uses a linear relationship representing GC12s and GC3s. If the slope is 0, the effect of direct mutation pressure is not present while if the slope of the linear relationship is 1, it means mutation pressure plays a major role. The higher the slope, the greater the effect of natural selection pressure [61]. Each dot represented one sequence of H7N9 HA gene or NA gene.

### 4.6. Potential Relationship between Host and Virus

#### 4.6.1. Codon Adaptation Index

CAI values are generally used to predict gene expression levels according to reference host RSCU values, ranging from 0 to 1.0. The CAI value was calculated by CAIcal server (http://genomes.urv.es/CAIcal/) [62]. The CAI value was calculated based on the reference value of the host. The higher the value, the stronger the adaptability of the corresponding host, and vice versa [63]. The reference RSCU was obtained from the Codon Usage Database (CUD) [64], in which the host species were *Homo sapiens* and *Gallus gallus*, as the existing hosts of H7N9.

#### 4.6.2. Relative Codon Deoptimization Index

The codon deoptimization trend is determined by comparing the codon usage of a given coding sequence with the reference genome. The RCDI was calculated by CAIcal server (http://genomes.urv.es/CAIcal/). Contrary to CAI, the value is ≥1. The larger the value, the weaker the adaptability to the host [65,66]. The reference RSCU value of the hosts *Homo sapiens* and *Gallus gallus* was obtained from CUD (http://www.kazusa.or.jp/codon/).

#### 4.6.3. Similarity Index

The Similarity index analysis is the effect of the overall codon usage pattern of the host on the codon usage of the virus. A common estimate of SiD is the cosine of the angle between A and B is:

$$R\left(A,B\right)=\frac{\sum_{i=1}^{59}a_{i}*b_{i}}{\sqrt{\sum_{i=1}^{59}a_{i}^{2}*\sum_{i=1}^{59}b_{i}^{2}}}$$

$$D\left(A,B\right)=\frac{1-R\left(A,B\right)}{2}$$

where *a_i_* denotes the RSCU value of a codon among 59 synonymous codons, and *b_i_* represents the RSCU value of corresponding codon of hosts (*Homo sapiens* and *Gallus gallus*). Overall, the D (A, B) is the value of SiD representing the influence of host to virus. It ranges from 0 to 1.0 [67].

#### 4.6.4. CpG Dinucleotides Frequency

The CpG content of each strain of HA and NA gene of H7N9 was calculated using DAMBE [68]. The ratio of CpG is divided by the observed value and the expected value. As mentioned above, the expected value was also obtained. When the relative dinucleotide abundances are >1.23 or <0.78 it indicates over-represented and under-represented dinucleotides, respectively [69].

## Figures and Tables

**Table 1:** Nucleotide composition.

Gene	Composition of A	Composition of A3	GC1s	GC2s	GC3s
HA	34.68% ± 0.21	48.14% ± 0.99	50.05% ± 0.268	41.20% ± 0.277	34.43% ± 0.796
NA	35.2% ± 0.20	49.42% ± 0.613	43.51% ± 0.398	47.26% ± 0.333	39.03% ± 0.624

**Table 2:** Relative synonymous codon usage analysis (RSCU) analysis on the basis of HA, (A) and NA (B) of three classifications: host, wave, and pathogenicity, as well as the hosts *Gallus gallus* and *Homo sapiens*. For the best synonymous codon RSCU value, fonts are bolded and italicized.

**(A)HA**	**All**	**Host**			**Pathogenicity**		**Wave**					**Reference Host**	
**Codon**		**Avian**	**Human**	**Environment**	**High**	**Low**	**Wave 1**	**Wave 2**	**Wave 3**	**Wave 4**	**Wave 5**	***Gallus gallus***	***Homo sapiens***
UUU(F)	0.69	0.71	0.68	0.67	0.64	0.69	0.72	0.72	0.68	0.65	0.64	0.91	0.93
***UUC(F)***	***1.31***	***1.29***	***1.32***	***1.33***	***1.36***	***1.31***	***1.28***	***1.28***	***1.32***	***1.35***	***1.36***	***1.09***	*****1.07*****
UUA(L)	0.5	0.52	0.48	0.49	0.49	0.5	0.46	0.51	0.5	0.48	0.48	0.45	0.46
UUG(L)	0.48	0.48	0.48	0.49	0.57	0.47	0.46	0.48	0.48	0.48	0.49	0.81	0.77
CUU(L)	0.67	0.65	0.67	0.71	0.81	0.66	0.62	0.64	0.68	0.64	0.72	0.80	0.79
CUC(L)	0.91	0.87	0.92	0.93	0.98	0.9	0.92	0.85	0.93	0.94	0.95	1.08	1.17
CUA(L)	1.55	1.59	1.54	1.46	1.3	1.56	1.69	1.68	1.45	1.43	1.41	0.38	0.43
***CUG(L)***	***1.9***	***1.89***	***1.9***	***1.92***	***1.85***	***1.9***	***1.85***	***1.84***	***1.95***	***2.03***	***1.95***	***2.48***	***2.37***
AUU(I)	1.09	1.11	1.08	1.08	1.06	1.09	1.12	1.11	1.09	1.06	1.05	1.06	1.08
AUC(I)	0.61	0.61	0.61	0.59	0.56	0.61	0.62	0.63	0.6	0.58	0.59	***1.39***	***1.41***
***AUA(I)***	***1.31***	***1.28***	***1.31***	***1.33***	***1.38***	***1.3***	***1.26***	***1.27***	***1.31***	***1.36***	***1.36***	0.55	0.51
GUU(V)	0.93	0.91	0.94	0.97	1.04	0.93	0.88	0.89	0.94	0.98	1	0.84	0.73
GUC(V)	0.75	0.75	0.75	0.74	0.7	0.75	0.75	0.75	0.75	0.76	0.75	0.87	0.95
GUA(V)	***1.17***	***1.22***	1.15	1.11	0.98	***1.18***	***1.25***	***1.23***	***1.2***	1.09	1.03	0.50	0.47
GUG(V)	1.15	1.12	***1.16***	***1.18***	***1.28***	1.14	1.12	1.13	1.11	***1.17***	***1.21***	***1.80***	***1.85***
UCU(S)	0.88	0.91	0.87	0.87	0.94	0.88	0.9	0.9	0.91	0.83	0.82	1.09	1.13
UCC(S)	0.19	0.16	0.2	0.23	0.19	0.19	0.14	0.15	0.16	0.27	0.27	1.21	1.31
UCA(S)	***1.78***	***1.79***	***1.77***	***1.76***	***1.74***	***1.78***	1.8	***1.8***	***1.76***	1.74	***1.74***	0.89	0.90
UCG(S)	0.22	0.19	0.24	0.26	0.3	0.22	0.15	0.16	0.27	0.31	0.32	0.40	0.33
AGU(S)	1.74	1.76	1.74	1.69	1.72	1.74	***1.81***	1.75	1.73	***1.75***	1.68	0.86	0.90
AGC(S)	1.19	1.2	1.19	1.2	1.11	1.2	1.2	1.24	1.16	1.09	1.17	***1.55***	***1.44***
CCU(P)	0.77	0.71	0.8	0.8	0.67	0.77	0.71	0.71	0.74	0.87	0.9	1.10	1.15
CCC(P)	0.43	0.49	0.4	0.41	0.62	0.42	0.47	0.47	0.47	0.31	0.31	***1.22***	***1.29***
***CCA(P)***	***2.11***	***2.1***	***2.11***	***2.1***	***2.04***	***2.11***	***2.11***	***2.11***	***2.08***	***2.12***	***2.11***	1.13	1.11
CCG(P)	0.7	0.71	0.69	0.69	0.66	0.7	0.71	0.71	0.7	0.7	0.67	0.56	0.45
ACU(T)	1.36	1.38	1.35	1.34	1.32	1.36	1.41	1.38	1.39	1.38	1.3	0.99	0.99
ACC(T)	0.73	0.72	0.73	0.76	0.79	0.72	0.7	0.71	0.72	0.69	0.76	***1.23***	***1.42***
***ACA(T)***	***1.88***	***1.88***	***1.89***	***1.88***	***1.78***	***1.89***	***1.88***	***1.88***	***1.88***	***1.92***	***1.91***	1.20	1.14
ACG(T)	0.03	0.02	0.04	0.03	0.12	0.03	0	0.03	0.01	0.01	0.03	0.57	0.46
GCU(A)	1.24	1.24	1.24	1.22	1.24	1.24	1.23	1.24	1.24	1.25	1.25	1.16	1.06
GCC(A)	0.56	0.56	0.56	0.57	0.55	0.56	0.55	0.55	0.55	0.56	0.57	***1.27***	***1.60***
***GCA(A)***	***1.88***	***1.89***	***1.87***	***1.87***	***1.89***	***1.88***	***1.89***	***1.9***	***1.88***	***1.85***	***1.85***	1.06	0.91
GCG(A)	0.33	0.32	0.33	0.34	0.33	0.33	0.33	0.32	0.33	0.34	0.33	0.51	0.42
***UAU(Y)***	***1.23***	***1.2***	***1.25***	***1.25***	***1.2***	***1.24***	***1.2***	***1.21***	***1.2***	***1.29***	***1.31***	0.80	0.89
UAC(Y)	0.77	0.8	0.75	0.75	0.8	0.76	0.8	0.79	0.8	0.71	0.69	***1.20***	***1.11***
***CAU(H)***	***1.37***	***1.39***	***1.37***	***1.36***	***1.4***	***1.37***	***1.4***	***1.4***	***1.4***	***1.4***	***1.33***	0.80	0.84
CAC(H)	0.63	0.61	0.63	0.64	0.6	0.63	0.6	0.6	0.6	0.6	0.67	***1.20***	***1.16***
***CAA(Q)***	***1.3***	***1.31***	***1.29***	***1.29***	***1.31***	***1.3***	***1.28***	***1.31***	***1.29***	***1.29***	***1.29***	0.54	0.53
CAG(Q)	0.7	0.69	0.71	0.71	0.69	0.7	0.72	0.69	0.71	0.71	0.71	***1.46***	***1.47***
***AAU(N)***	***1.28***	***1.28***	***1.28***	***1.29***	***1.32***	***1.28***	***1.29***	***1.27***	***1.29***	***1.27***	***1.27***	0.86	0.94
AAC(N)	0.72	0.72	0.72	0.71	0.68	0.72	0.71	0.73	0.71	0.73	0.73	***1.14***	***1.06***
***AAA(K)***	***1.32***	***1.3***	***1.33***	***1.31***	***1.32***	***1.32***	***1.36***	***1.31***	***1.35***	***1.28***	***1.3***	0.89	0.87
AAG(K)	0.68	0.7	0.67	0.69	0.68	0.68	0.64	0.69	0.65	0.72	0.7	***1.11***	***1.13***
***GAU(D)***	***1.25***	***1.23***	***1.25***	***1.25***	***1.22***	***1.25***	***1.22***	***1.25***	***1.23***	***1.27***	***1.29***	***1.01***	0.93
GAC(D)	0.75	0.77	0.75	0.75	0.78	0.75	0.78	0.75	0.77	0.73	0.71	0.99	***1.07***
***GAA(E)***	***1.38***	***1.4***	***1.37***	***1.37***	***1.44***	***1.38***	***1.4***	***1.4***	***1.39***	***1.35***	***1.34***	0.86	0.84
GAG(E)	0.62	0.6	0.63	0.63	0.56	0.62	0.6	0.6	0.61	0.65	0.66	***1.14***	*****1.16*****
***UGU(C)***	***1.37***	***1.37***	***1.37***	***1.37***	***1.38***	***1.37***	***1.37***	***1.37***	***1.38***	***1.36***	***1.36***	0.80	0.91
UGC(C)	0.63	0.63	0.63	0.63	0.62	0.63	0.63	0.63	0.62	0.64	0.64	***1.20***	***1.09***
CGU(R)	0.2	0.2	0.2	0.2	0.18	0.2	0.2	0.21	0.2	0.2	0.2	0.59	0.48
CGC(R)	0	0	0	0	0	0	0	0	0	0	0	1.14	1.10
CGA(R)	0.56	0.6	0.55	0.52	0.55	0.56	0.6	0.62	0.59	0.58	0.47	0.58	0.65
CGG(R)	0.51	0.44	0.54	0.57	0.56	0.51	0.4	0.41	0.39	0.58	0.71	1.07	1.21
***AGA(R)***	***3.31***	***3.28***	***3.32***	***3.33***	***3.4***	***3.3***	***3.23***	***3.33***	***3.25***	***3.35***	***3.4***	***1.34***	***1.29***
AGG(R)	1.42	1.47	1.4	1.38	1.31	1.42	1.57	1.43	1.57	1.3	1.22	1.29	1.27
GGU(G)	0.67	0.65	0.67	0.68	0.65	0.67	0.64	0.64	0.66	0.71	0.71	0.70	0.65
GGC(G)	0.48	0.48	0.47	0.48	0.49	0.48	0.48	0.48	0.48	0.48	0.47	***1.22***	***1.35***
***GGA(G)***	***1.89***	***1.97***	***1.87***	***1.83***	***1.88***	***1.9***	***2***	***2***	***1.87***	***1.71***	***1.73***	1.09	1.00
GGG(G)	0.96	0.91	0.98	1.01	0.98	0.96	0.88	0.88	0.99	1.09	1.09	0.99	1.00
(**B)NA**	**All**	**Host**			**Pathogenicity**		**Wave**					**Reference host**	
**Codon**		**Avian**	**Human**	**Environment**	**High**	**Low**	**Wave 1**	**Wave 2**	**Wave 3**	**Wave 4**	**Wave 5**	***Gallus gallus***	***Homo sapiens***
UUU(F)	0.49	0.5	0.47	0.51	0.46	0.51	0.44	0.48	0.46	0.46	0.55	0.91	0.93
***UUC(F)***	***1.51***	***1.5***	***1.53***	***1.49***	***1.54***	***1.49***	***1.56***	***1.52***	***1.54***	***1.54***	***1.45***	***1.09***	***1.07***
UUA(L)	1.02	1.03	1.01	1.06	1.03	1.03	0.98	1	1	1.14	1.06	0.45	0.46
UUG(L)	0.95	0.95	0.98	0.91	0.79	0.94	1	1.01	1	1.02	0.86	0.81	0.77
CUU(L)	0.26	0.26	0.24	0.25	0.26	0.27	0.25	0.25	0.25	0.25	0.28	0.80	0.79
CUC(L)	0.99	0.99	1.01	0.99	1.03	0.98	1	1	1	1	0.97	1.08	1.17
***CUA(L)***	***1.51***	***1.51***	***1.51***	***1.51***	1.4	***1.52***	***1.52***	***1.5***	***1.49***	***1.35***	***1.54***	0.38	0.43
CUG(L)	1.26	1.26	1.25	1.29	***1.49***	1.26	1.25	1.25	1.26	1.23	1.28	***2.48***	***2.37***
AUU(I)	0.82	0.83	0.8	0.84	0.74	0.84	0.81	0.79	0.83	0.79	0.86	1.06	1.08
AUC(I)	0.42	0.42	0.41	0.42	0.48	0.42	0.41	0.42	0.42	0.42	0.43	***1.39***	***1.41***
***AUA(I)***	***1.76***	***1.75***	***1.79***	***1.74***	***1.78***	***1.74***	***1.78***	***1.79***	***1.76***	***1.79***	***1.7***	0.55	0.51
GUU(V)	0.7	0.71	0.71	0.69	0.58	0.71	0.72	0.71	0.71	0.78	0.7	0.84	0.73
GUC(V)	0.33	0.32	0.34	0.34	0.48	0.3	0.34	0.31	0.33	0.33	0.3	0.87	0.95
***GUA(V)***	***1.66***	***1.68***	***1.64***	***1.65***	***1.66***	***1.66***	***1.73***	***1.64***	***1.71***	***1.67***	***1.64***	0.50	0.47
GUG(V)	1.3	1.3	1.32	1.31	1.28	1.32	1.21	1.33	1.26	1.21	1.36	***1.80***	***1.85***
UCU(S)	0.73	0.71	0.79	0.69	0.69	0.68	0.83	0.84	0.7	0.59	0.59	1.09	1.13
UCC(S)	0.46	0.48	0.44	0.48	0.43	0.49	0.42	0.42	0.45	0.53	0.54	1.21	1.31
***UCA(S)***	***2.07***	***2.07***	***2.09***	***2.06***	***2.03***	***2.06***	***2.07***	***2.1***	***2.1***	***2.06***	***2.03***	0.89	0.90
UCG(S)	0.43	0.43	0.42	0.43	0.52	0.43	0.43	0.4	0.41	0.44	0.45	0.40	0.33
AGU(S)	1.19	1.21	1.13	1.23	1.23	1.24	1.11	1.09	1.24	1.37	1.32	0.86	0.90
AGC(S)	1.12	1.1	1.14	1.11	1.1	1.1	1.14	1.16	1.1	1.01	1.07	*****1.55*****	*****1.44*****
CCU(P)	1.03	1.03	1.02	1.04	1.13	1.03	1	0.99	1.1	1.08	1.04	1.10	1.15
CCC(P)	0.84	0.85	0.83	0.85	0.84	0.86	0.83	0.84	0.81	0.81	0.88	***1.22***	***1.29***
***CCA(P)***	***1.61***	***1.6***	***1.65***	***1.59***	***1.66***	***1.58***	***1.67***	***1.68***	***1.61***	***1.62***	***1.51***	1.13	1.11
CCG(P)	0.52	0.53	0.5	0.51	0.37	0.54	0.5	0.49	0.49	0.49	0.57	0.56	0.45
ACU(T)	1.05	1.02	1.12	1.02	1.01	1.02	1.07	1.12	1.06	1.05	0.96	0.99	0.99
ACC(T)	0.54	0.55	0.51	0.54	0.51	0.55	0.55	0.51	0.53	0.54	0.57	***1.23***	***1.42***
***ACA(T)***	***2.4***	***2.42***	***2.36***	***2.43***	***2.47***	***2.42***	***2.38***	***2.37***	***2.4***	***2.4***	***2.45***	1.20	1.14
ACG(T)	0.01	0.01	0.01	0.01	0.01	0.01	0	0	0.01	0.01	0.02	0.57	0.46
GCU(A)	1.2	1.2	1.21	1.18	1.17	1.19	1.21	1.22	1.18	1.18	1.18	1.16	1.06
GCC(A)	0.87	0.87	0.87	0.89	0.93	0.86	0.87	0.86	0.89	0.88	0.86	***1.27***	***1.60***
***GCA(A)***	***1.76***	***1.76***	***1.75***	***1.76***	***1.71***	***1.77***	***1.74***	***1.74***	***1.75***	***1.76***	***1.8***	1.06	0.91
GCG(A)	0.17	0.17	0.17	0.17	0.2	0.17	0.18	0.17	0.18	0.17	0.16	0.51	0.42
***UAU(Y)***	***1.21***	***1.21***	***1.2***	***1.22***	***1.2***	***1.22***	***1.2***	***1.2***	***1.21***	***1.23***	***1.22***	0.80	0.89
UAC(Y)	0.79	0.79	0.8	0.78	0.8	0.78	0.8	0.8	0.79	0.77	0.78	***1.20***	***1.11***
CAU(H)	0.65	0.64	0.67	0.66	0.73	0.63	0.67	0.67	0.7	0.62	0.6	0.80	0.84
***CAC(H)***	***1.35***	***1.36***	***1.33***	***1.34***	***1.27***	***1.37***	***1.33***	***1.33***	***1.3***	***1.38***	***1.4***	***1.20***	***1.16***
***CAA(Q)***	***1.08***	***1.08***	***1.08***	***1.08***	***1.08***	***1.08***	***1.05***	***1.08***	***1.09***	***1.07***	***1.08***	0.54	0.53
CAG(Q)	0.92	0.92	0.92	0.92	0.92	0.92	0.95	0.92	0.91	0.93	0.92	***1.46***	***1.47***
AAU(N)	0.96	0.96	0.97	0.94	0.86	0.95	0.99	0.98	0.96	0.94	0.92	0.86	0.94
***AAC(N)***	***1.04***	***1.04***	***1.03***	***1.06***	***1.14***	***1.05***	***1.01***	***1.02***	***1.04***	***1.06***	***1.08***	***1.14***	***1.06***
***AAA(K)***	***1.26***	***1.26***	***1.26***	***1.26***	***1.31***	***1.25***	***1.29***	***1.26***	***1.28***	***1.28***	***1.23***	0.89	0.87
AAG(K)	0.74	0.74	0.74	0.74	0.69	0.75	0.71	0.74	0.72	0.72	0.77	***1.11***	***1.13***
GAU(D)	0.97	0.98	0.96	0.98	0.93	0.98	0.97	0.95	0.92	0.95	***1.01***	***1.01***	0.93
***GAC(D)***	***1.03***	***1.02***	***1.04***	***1.02***	***1.07***	***1.02***	***1.03***	***1.05***	***1.08***	***1.05***	0.99	0.99	***1.07***
***GAA(E)***	***1.33***	***1.35***	***1.3***	***1.35***	***1.35***	***1.35***	***1.27***	***1.26***	***1.4***	***1.43***	***1.37***	0.86	0.84
GAG(E)	0.67	0.65	0.7	0.65	0.65	0.65	0.73	0.74	0.6	0.57	0.63	***1.14***	*****1.16*****
UGU(C)	0.65	0.65	0.67	0.64	0.67	0.64	0.66	0.66	0.67	0.67	0.62	0.80	0.91
***UGC(C)***	***1.35***	***1.35***	***1.33***	***1.36***	***1.33***	***1.36***	***1.34***	***1.34***	***1.33***	***1.33***	***1.38***	***1.20***	***1.09***
CGU(R)	0.24	0.24	0.24	0.24	0.26	0.24	0.24	0.24	0.24	0.24	0.25	0.59	0.48
CGC(R)	0.25	0.24	0.25	0.27	0.45	0.24	0.24	0.24	0.32	0.24	0.23	1.14	1.10
CGA(R)	0.96	0.96	0.95	0.96	0.98	0.96	0.97	0.96	0.94	0.98	0.97	0.58	0.65
CGG(R)	0.01	0.01	0.01	0.02	0.01	0.01	0	0	0	0	0.02	1.07	1.21
***AGA(R)***	***2.43***	***2.41***	***2.49***	***2.42***	***2.46***	***2.4***	***2.4***	***2.49***	***2.38***	***2.4***	***2.38***	***1.34***	***1.29***
AGG(R)	2.11	2.13	2.06	2.09	1.85	2.14	2.16	2.06	2.12	2.13	2.15	1.29	1.27
GGU(G)	0.46	0.46	0.45	0.45	0.44	0.46	0.45	0.47	0.46	0.47	0.46	0.70	0.65
GGC(G)	0.55	0.55	0.54	0.54	0.54	0.54	0.54	0.54	0.55	0.54	0.55	***1.22***	***1.35***
***GGA(G)***	***1.7***	***1.67***	***1.76***	***1.63***	***1.47***	***1.65***	***1.78***	***1.82***	***1.61***	***1.56***	***1.56***	1.09	1.00
GGG(G)	1.3	1.32	1.24	1.38	1.55	1.34	1.23	1.17	1.38	1.43	1.43	0.99	1.00

## Supplementary Materials

The following are available online at https://www.mdpi.com/1422-0067/21/19/7129/s1. Table S1. Host, country, and date of HA and NA sequences used in this study. Table S2. Nucleotide composition of HA and NA sequences and relevant plotting data.

## References

[1] Belser J.A., Bridges C.B., Katz J.M., Tumpey T.M. (2009). Past, Present, and Possible Future Human Infection with Influenza Virus A Subtype H7. Emerg. Infect. Dis..

[2] Gao R., Cao B., Hu Y., Feng Z., Wang D., Hu W., Chen J., Jie Z., Qiu H., Xu K. (2013). Human infection with a novel avian-origin influenza A (H7N9) virus. N. Engl. J. Med..

[3] Liu J., Xiao H., Wu Y., Liu D., Qi X., Shi Y., Gao G.F. (2014). H7N9: A low pathogenic avian influenza A virus infecting humans. Curr. Opin. Virol..

[4] Liu D., Shi W., Shi Y., Wang D., Xiao H., Li W., Bi Y., Wu Y., Li X., Yan J. (2013). Origin and diversity of novel avian influenza A H7N9 viruses causing human infection: Phylogenetic, structural, and coalescent analyses. Lancet.

[5] Su S., Gu M., Liu D., Cui J., Gao G.F., Zhou J.Y., Liu X.F. (2017). Epidemiology, Evolution, and Pathogenesis of H7N9 Influenza Viruses in Five Epidemic Waves since 2013 in China. Trends Microbiol..

[6] Gao G.F. (2014). Influenza and the Live Poultry Trade. Science.

[7] Li J., Yu X.F., Pu X.Y., Xie L., Sun Y.X., Xiao H.X., Wang F.J., Din H., Wu Y., Liu D. (2013). Environmental connections of novel avian-origin H7N9 influenza virus infection and virus adaptation to the human. Sci. China-Life Sci..

[8] Wang X.L., Jiang H., Wu P., Uyeki T.M., Feng L.Z., Lai S.J., Wang L.L., Huo X., Xu K., Chen E.F. (2017). Epidemiology of avian influenza A H7N9 virus in human beings across five epidemics in mainland China, 2013–2017: An epidemiological study of laboratory-confirmed case series. Lancet Infect. Dis..

[9] Van Ranst M., Lemey P. (2013). Genesis of avian-origin H7N9 influenza A viruses. Lancet.

[10] Wu Y., Gao G.F. (2013). Lessons learnt from the human infections of avian-origin influenza A H7N9 virus: Live free markets and human health. Sci. China-Life Sci..

[11] Su S., Wong G., Shi W., Liu J., Lai A.C.K., Zhou J., Liu W., Bi Y., Gao G.F. (2016). Epidemiology, Genetic Recombination, and Pathogenesis of Coronaviruses. Trends Microbiol..

[12] Su S., Bi Y., Wong G., Gray G.C., Gao G.F., Li S. (2015). Epidemiology, Evolution, and Recent Outbreaks of Avian Influenza Virus in China. J. Virol..

[13] Sun J., He W.T., Wang L., Lai A., Ji X., Zhai X., Li G., Suchard M.A., Tian J., Zhou J. (2020). COVID-19: Epidemiology, Evolution, and Cross-Disciplinary Perspectives. Trends Mol. Med..

[14] Zhai X., Sun J., Yan Z., Zhang J., Zhao J., Zhao Z., Gao Q., He W.T., Veit M., Su S. (2020). Comparison of Severe Acute Respiratory Syndrome Coronavirus 2 Spike Protein Binding to ACE2 Receptors from Human, Pets, Farm Animals, and Putative Intermediate Hosts. J. Virol..

[15] He W.T., Ji X., He W., Dellicour S., Wang S., Li G., Zhang L., Gilbert M., Zhu H., Xing G. (2020). Genomic Epidemiology, Evolution, and Transmission Dynamics of Porcine Deltacoronavirus. Mol. Biol. Evol..

[16] He W., Auclert L.Z., Zhai X., Wong G., Zhang C., Zhu H., Xing G., Wang S., He W., Li K. (2019). Interspecies Transmission, Genetic Diversity, and Evolutionary Dynamics of Pseudorabies Virus. J. Infect. Dis..

[17] Li G., He W., Zhu H., Bi Y., Wang R., Xing G., Zhang C., Zhou J., Yuen K.Y., Gao G.F. (2018). Origin, Genetic Diversity, and Evolutionary Dynamics of Novel Porcine Circovirus 3. Adv. Sci. (Weinh. Baden-Wurtt. Ger.).

[18] Quan C., Shi W., Yang Y., Yang Y., Liu X., Xu W., Li H., Li J., Wang Q., Tong Z. (2018). New Threats from H7N9 Influenza Virus: Spread and Evolution of High- and Low-Pathogenicity Variants with High Genomic Diversity in Wave Five. J. Virol..

[19] Yang H., Carney P.J., Chang J.C., Villanueva J.M., Stevens J. (2013). Structural Analysis of the Hemagglutinin from the Recent 2013 H7N9 Influenza Virus. J. Virol..

[20] Wagner R., Matrosovich M., Klenk H.D. (2002). Functional balance between haemagglutinin and neuraminidase in influenza virus infections. Rev. Med. Virol..

[21] Wu X.M., Wu S.F., Ren D.M., Zhu Y.P., He F.C. (2007). The analysis method and progress in the study of codon bias. Yi Chuan.

[22] Hershberg R., Petrov D.A. (2008). Selection on codon bias. Annu. Rev. Genet..

[23] Plotkin J.B., Kudla G. (2011). Synonymous but not the same: The causes and consequences of codon bias. Nat. Rev. Genet..

[24] Li G., Wang H., Wang S., Xing G., Zhang C., Zhang W., Liu J., Zhang J., Su S., Zhou J. (2018). Insights into the genetic and host adaptability of emerging porcine circovirus 3. Virulence.

[25] Butt A.M., Nasrullah I., Qamar R., Tong Y.G. (2016). Evolution of codon usage in Zika virus genomes is host and vector specific. Emerg. Microbes Infect..

[26] Kumar N., Kulkarni D.D., Lee B., Kaushik R., Bhatia S., Sood R., Pateriya A.K., Bhat S., Singh V.P. (2018). Evolution of Codon Usage Bias in Henipaviruses Is Governed by Natural Selection and Is Host-Specific. Viruses.

[27] Bera B.C., Virmani N., Kumar N., Anand T., Pavulraj S., Rash A., Elton D., Rash N., Bhatia S., Sood R. (2017). Genetic and codon usage bias analyses of polymerase genes of equine influenza virus and its relation to evolution. BMC Genom..

[28] Zhou Y., Chen X., Ushijima H., Frey T.K. (2012). Analysis of base and codon usage by rubella virus. Arch. Virol..

[29] Chen F., Wu P., Deng S., Zhang H., Hou Y., Hu Z., Zhang J., Chen X., Yang J.R. (2020). Dissimilation of synonymous codon usage bias in virus-host coevolution due to translational selection. Nat. Ecol. Evol..

[30] Chaney J.L., Clark P.L. (2015). Roles for Synonymous Codon Usage in Protein Biogenesis. Annu. Rev. Biophys..

[31] Gun L., Haixian P., Yumiao R., Han T., Jingqi L., Liguang Z. (2018). Codon usage characteristics of PB2 gene in influenza A H7N9 virus from different host species. Infect. Genet. Evol. J. Mol. Epidemiol. Evol. Genet. Infect. Dis..

[32] Zhu H.N., Hughes J., Murcia P.R. (2015). Origins and Evolutionary Dynamics of H3N2 Canine Influenza Virus. J. Virol..

[33] Lam T.T., Wang J., Shen Y., Zhou B., Duan L., Cheung C.L., Ma C., Lycett S.J., Leung C.Y., Chen X. (2013). The genesis and source of the H7N9 influenza viruses causing human infections in China. Nature.

[34] Li G.R., Wang R.Y., Zhang C., Wang S.L., He W.T., Zhang J.Y., Liu J., Cai Y.C., Zhou J.Y., Su S. (2018). Genetic and evolutionary analysis of emerging H3N2 canine influenza virus. Emerg. Microbes Infect..

[35] Anhlan D., Grundmann N., Makalowski W., Ludwig S., Scholtissek C. (2011). Origin of the 1918 pandemic H1N1 influenza A virus as studied by codon usage patterns and phylogenetic analysis. RNA.

[36] Kumar N., Bera B.C., Greenbaum B.D., Bhatia S., Sood R., Selvaraj P., Anand T., Tripathi B.N., Virmani N. (2016). Revelation of Influencing Factors in Overall Codon Usage Bias of Equine Influenza Viruses. PLoS ONE.

[37] Zhou T., Gu W.J., Ma J.M., Sun X., Lu Z.H. (2005). Analysis of synonymous codon usage in H5N1 virus and other influenza A viruses. Biosystems.

[38] Zhang W., Zhang L., He W., Zhang X., Wen B., Wang C., Xu Q., Li G., Zhou J., Veit M. (2019). Genetic Evolution and Molecular Selection of the HE Gene of Influenza C Virus. Viruses.

[39] Yan Z., Wang R., Zhang L., Shen B., Wang N., Xu Q., He W., He W., Li G., Su S. (2019). Evolutionary changes of the novel Influenza D virus hemagglutinin-esterase fusion gene revealed by the codon usage pattern. Virulence.

[40] Jenkins G.M., Holmes E.C. (2003). The extent of codon usage bias in human RNA viruses and its evolutionary origin. Virus Res..

[41] Hu J.S., Wang Q.Q., Zhang J., Chen H.T., Xu Z.W., Zhu L., Ding Y.Z., Ma L.N., Xu K., Gu Y.X. (2011). The characteristic of codon usage pattern and its evolution of hepatitis C virus. Infect. Genet. Evol..

[42] Yang L., Zhu W., Li X., Chen M., Wu J., Yu P., Qi S., Huang Y., Shi W., Dong J. (2017). Genesis and Spread of Newly Emerged Highly Pathogenic H7N9 Avian Viruses in Mainland China. J. Virol..

[43] Franzo G., Tucciarone C.M., Cecchinato M., Drigo M. (2017). Canine parvovirus type 2 (CPV-2) and Feline panleukopenia virus (FPV) codon bias analysis reveals a progressive adaptation to the new niche after the host jump. Mol. Phylogenet. Evol..

[44] Zheng Z., Lu Y., Short K.R., Lu J. (2019). One health insights to prevent the next HxNy viral outbreak: Learning from the epidemiology of H7N9. BMC Infect. Dis..

[45] Greenbaum B.D., Levine A.J., Bhanot G., Rabadan R. (2008). Patterns of evolution and host gene mimicry in influenza and other RNA viruses. PLoS Pathog..

[46] Katoh K., Standley D.M. (2013). MAFFT multiple sequence alignment software version 7: Improvements in performance and usability. Mol. Biol. Evol..

[47] Stamatakis A. (2014). RAxML Version 8: A Tool for Phylogenetic Analysis and Post-Analysis of Large Phylogenies. Bioinformatics.

[48] Keane T.M., Creevey C.J., Pentony M.M., Naughton T.J., McLnerney J.O. (2006). Assessment of methods for amino acid matrix selection and their use on empirical data shows that ad hoc assumptions for choice of matrix are not justified. BMC Evol. Biol..

[49] Li G., Ji S., Zhai X., Zhang Y., Liu J., Zhu M., Zhou J., Su S. (2017). Evolutionary and genetic analysis of the VP2 gene of canine parvovirus. BMC Genom..

[50] Dave U., Srivathsan A., Kumar S. (2019). Analysis of codon usage pattern in the viral proteins of chicken anaemia virus and its possible biological relevance. Infect. Genet. Evol..

[51] Sharp P.M., Li W.H. (1986). Codon Usage in Regulatory Genes in Escherichia-Coli Does Not Reflect Selection for Rare Codons. Nucleic Acids Res..

[52] Sharp P.M., Li W.H. (1986). An Evolutionary Perspective on Synonymous Codon Usage in Unicellular Organisms. J. Mol. Evol..

[53] Wong E.H.M., Smith D.K., Rabadan R., Peiris M., Poon L.L.M. (2010). Codon usage bias and the evolution of influenza A viruses. Codon Usage Biases of Influenza Virus. BMC Evol. Biol..

[54] Wright F. (1990). The ‘effective number of codons’ used in a gene. Gene.

[55] Comeron J.M., Aguade M. (1998). An evaluation of measures of synonymous codon usage bias. J. Mol. Evol..

[56] Ma J.J., Zhao F., Zhang J., Zhou J.H., Ma L.N., Ding Y.Z., Chen H.T., Gu Y.X., Liu Y.S. (2013). Analysis of Synonymous Codon Usage in Dengue Viruses. J. Anim. Vet. Adv..

[57] Nasrullah I., Butt A.M., Tahir S., Idrees M., Tong Y.G. (2015). Genomic analysis of codon usage shows influence of mutation pressure, natural selection, and host features on Marburg virus evolution. BMC Evol. Biol..

[58] Moratorio G., Iriarte A., Moreno P., Musto H., Cristina J. (2013). A detailed comparative analysis on the overall codon usage patterns in West Nile virus. Infect. Genet. Evol..

[59] Sueoka N. (1995). Intrastrand parity rules of DNA base composition and usage biases of synonymous codons. J. Mol. Evol..

[60] Sueoka N. (1999). Translation-coupled violation of Parity Rule 2 in human genes is not the cause of heterogeneity of the DNA G plus C content of third codon position. Gene.

[61] Sueoka N. (1988). Directional mutation pressure and neutral molecular evolution. Proc. Natl. Acad. Sci. USA.

[62] Puigbo P., Bravo I.G., Garcia-Vallve S. (2008). CAIcal: A combined set of tools to assess codon usage adaptation. Biol. Direct.

[63] Sharp P.M., Li W.H. (1987). The Codon Adaptation Index—A Measure of Directional Synonymous Codon Usage Bias, and Its Potential Applications. Nucleic Acids Res..

[64] Nakamura Y., Gojobori T., Ikemura T. (2000). Codon usage tabulated from international DNA sequence databases: Status for the year 2000. Nucleic Acids Res..

[65] Puigbo P., Aragones L., Garcia-Vallve S. (2010). RCDI/eRCDI: A web-server to estimate codon usage deoptimization. BMC Res. Notes.

[66] Mueller S., Papamichail D., Coleman J.R., Skiena S., Wimmer E. (2006). Reduction of the rate of poliovirus protein synthesis through large-scale codon deoptimization causes attenuation of viral virulence by lowering specific infectivity. J. Virol..

[67] Zhou J.H., Zhang J., Sun D.J., Ma Q., Chen H.T., Ma L.N., Ding Y.Z., Liu Y.S. (2013). The Distribution of Synonymous Codon Choice in the Translation Initiation Region of Dengue Virus. PLoS ONE.

[68] Karlin S., Burge C. (1995). Dinucleotide relative abundance extremes: A genomic signature. Trends Genet..

[69] Karlin S., Doerfler W., Cardon L.R. (1994). Why is CpG suppressed in the genomes of virtually all small eukaryotic viruses but not in those of large eukaryotic viruses?. J. Virol..

