# Supplementary material for: Analysis of the Codon Usage Pattern of HA and NA Genes of H7N9 Influenza A Virus
Source: Int J Mol Sci. 2020 Sep 27;21(19):7129. doi: 10.3390/ijms21197129 (PMC7583936; doi:10.3390/ijms21197129)
Supplement: Supplementary file 1 [file ijms-21-07129-s001.zip › TableS1-HA.docx]

| Strain name-HA | Host | Province | Date |
| --- | --- | --- | --- |
| A/Anhui/01/2013 | Human | Anhui | 2013 |
| A/Anhui/01872/2014-02-19 | Human | Anhui | 2014/2/19 |
| A/Anhui/01876/2014-03-10 | Human | Anhui | 2014/3/10 |
| A/Anhui/01878/2014-04-02 | Human | Anhui | 2014/4/2 |
| A/Anhui/01879/2014-04-02 | Human | Anhui | 2014/4/2 |
| A/Anhui/01881/2014-04-13 | Human | Anhui | 2014/4/13 |
| A/Anhui/01882/2014-04-26 | Human | Anhui | 2014/4/26 |
| A/Anhui/01883/2014-04-26 | Human | Anhui | 2014/4/26 |
| A/Anhui/01887/2014-05-20 | Human | Anhui | 2014/5/20 |
| A/Anhui/02/2013-04-14 | Human | Anhui | 2013/4/14 |
| A/Anhui/03/2013-04-21 | Human | Anhui | 2013/4/21 |
| A/Anhui/04/2013-04-01 | Human | Anhui | 2013/4/1 |
| A/Anhui/09186/2014-02-07 | Human | Anhui | 2014/2/7 |
| A/Anhui/09188/2014-02-07 | Human | Anhui | 2014/2/7 |
| A/Anhui/09212/2014-02-13 | Human | Anhui | 2014/2/13 |
| A/Anhui/09214/2014-02-15 | Human | Anhui | 2014/2/15 |
| A/Anhui/1/2013_NIIDRG-10.1 | Human | Anhui | 2013 |
| A/Anhui/1/2013-03-20 | Human | Anhui | 2013/3/20 |
| A/Anhui/13420/2017-01-14 | Human | Anhui | 2017/1/14 |
| A/Anhui/13421/2017-01-20 | Human | Anhui | 2017/1/20 |
| A/Anhui/13422/2017-02-20 | Human | Anhui | 2017/2/20 |
| A/Anhui/13423/2017-01-13 | Human | Anhui | 2017/1/13 |
| A/Anhui/13424/2017-02-14 | Human | Anhui | 2017/2/14 |
| A/Anhui/13425/2017-02-14 | Human | Anhui | 2017/2/14 |
| A/Anhui/13426/2017-02-14 | Human | Anhui | 2017/2/14 |
| A/Anhui/13427/2017-02-14 | Human | Anhui | 2017/2/14 |
| A/Anhui/13428/2017-02-15 | Human | Anhui | 2017/2/15 |
| A/Anhui/13431/2017-01-13 | Human | Anhui | 2017/1/13 |
| A/Anhui/13432/2017-01-17 | Human | Anhui | 2017/1/17 |
| A/Anhui/13433/2017-01-17 | Human | Anhui | 2017/1/17 |
| A/Anhui/13434/2017-01-31 | Human | Anhui | 2017/1/31 |
| A/Anhui/13436/2016-12-31 | Human | Anhui | 2016/12/31 |
| A/Anhui/13439/2017-01-06 | Human | Anhui | 2017/1/6 |
| A/Anhui/13441/2017-01-13 | Human | Anhui | 2017/1/13 |
| A/Anhui/13442/2017-01-13 | Human | Anhui | 2017/1/13 |
| A/Anhui/13443/2017-01-13 | Human | Anhui | 2017/1/13 |
| A/Anhui/13444/2017-02-20 | Human | Anhui | 2017/2/20 |
| A/Anhui/13446/2017-01-20 | Human | Anhui | 2017/1/20 |
| A/Anhui/13447/2017-01-27 | Human | Anhui | 2017/1/27 |
| A/Anhui/13449/2017-02-05 | Human | Anhui | 2017/2/5 |
| A/Anhui/1-BALF_RG1/2013 | Human | Anhui | 2013 |
| A/Anhui/1-BALF_RG17/2013 | Human | Anhui | 2013 |
| A/Anhui/1-BALF_RG18/2013 | Human | Anhui | 2013 |
| A/Anhui/1-BALF_RG19/2013 | Human | Anhui | 2013 |
| A/Anhui/1-BALF_RG2/2013 | Human | Anhui | 2013 |
| A/Anhui/1-BALF_RG20/2013 | Human | Anhui | 2013 |
| A/Anhui/1-BALF_RG21/2013 | Human | Anhui | 2013 |
| A/Anhui/1-BALF_RG29/2013 | Human | Anhui | 2013 |
| A/Anhui/1-BALF_RG3/2013 | Human | Anhui | 2013 |
| A/Anhui/1-BALF_RG31/2013 | Human | Anhui | 2013 |
| A/Anhui/1-BALF_RG32/2013 | Human | Anhui | 2013 |
| A/Anhui/1-BALF_RG33/2013 | Human | Anhui | 2013 |
| A/Anhui/1-BALF_RG4/2013 | Human | Anhui | 2013 |
| A/Anhui/1-BALF_RG40/2013 | Human | Anhui | 2013 |
| A/Anhui/1-BALF_RG41/2013 | Human | Anhui | 2013 |
| A/Anhui/1-BALF_RG42/2013 | Human | Anhui | 2013 |
| A/Anhui/1-BALF_RG43/2013 | Human | Anhui | 2013 |
| A/Anhui/1-BALF_RG44/2013 | Human | Anhui | 2013 |
| A/Anhui/1-BALF_RG45/2013 | Human | Anhui | 2013 |
| A/Anhui/1-BALF_RG5/2013 | Human | Anhui | 2013 |
| A/Anhui/1-BALF_RG6/2013 | Human | Anhui | 2013 |
| A/Anhui/1-BALF_RG7/2013 | Human | Anhui | 2013 |
| A/Anhui/1-BALF_RG8/2013 | Human | Anhui | 2013 |
| A/Anhui/1-BALF_RG9/2013 | Human | Anhui | 2013 |
| A/Anhui/1-DEWH730/2013 | Human | Anhui | 2013 |
| A/Anhui/1-JCVI1_RG1/2013 | Human | Anhui | 2013 |
| A/Anhui/1-JCVI1_RG2/2013 | Human | Anhui | 2013 |
| A/Anhui/1-JCVI1_RG3/2013 | Human | Anhui | 2013 |
| A/Anhui/1-YK_RG01/2013 | Human | Anhui | 2013 |
| A/Anhui/1-YK_RG02/2013 | Human | Anhui | 2013 |
| A/Anhui/1-YK_RG03/2013 | Human | Anhui | 2013 |
| A/Anhui/1-YK_RG04/2013 | Human | Anhui | 2013 |
| A/Anhui/1-YK_RG05/2013 | Human | Anhui | 2013 |
| A/Anhui/1-YK_RG06/2013 | Human | Anhui | 2013 |
| A/Anhui/1-YK_RG07/2013 | Human | Anhui | 2013 |
| A/Anhui/1-YK_RG08/2013 | Human | Anhui | 2013 |
| A/Anhui/1-YK_RG09/2013 | Human | Anhui | 2013 |
| A/Anhui/1-YK_RG10/2013 | Human | Anhui | 2013 |
| A/Anhui/1-YK_RG100/2013 | Human | Anhui | 2013 |
| A/Anhui/1-YK_RG101/2013 | Human | Anhui | 2013 |
| A/Anhui/1-YK_RG102/2013 | Human | Anhui | 2013 |
| A/Anhui/1-YK_RG103/2013 | Human | Anhui | 2013 |
| A/Anhui/1-YK_RG104/2013 | Human | Anhui | 2013 |
| A/Anhui/1-YK_RG105/2013 | Human | Anhui | 2013 |
| A/Anhui/1-YK_RG106/2013 | Human | Anhui | 2013 |
| A/Anhui/1-YK_RG107/2013 | Human | Anhui | 2013 |
| A/Anhui/1-YK_RG108/2013 | Human | Anhui | 2013 |
| A/Anhui/1-YK_RG109/2013 | Human | Anhui | 2013 |
| A/Anhui/1-YK_RG110/2013 | Human | Anhui | 2013 |
| A/Anhui/1-YK_RG111/2013 | Human | Anhui | 2013 |
| A/Anhui/1-YK_RG112/2013 | Human | Anhui | 2013 |
| A/Anhui/1-YK_RG113/2013 | Human | Anhui | 2013 |
| A/Anhui/1-YK_RG114/2013 | Human | Anhui | 2013 |
| A/Anhui/1-YK_RG115/2013 | Human | Anhui | 2013 |
| A/Anhui/1-YK_RG116/2013 | Human | Anhui | 2013 |
| A/Anhui/1-YK_RG117/2013 | Human | Anhui | 2013 |
| A/Anhui/1-YK_RG118/2013 | Human | Anhui | 2013 |
| A/Anhui/1-YK_RG119/2013 | Human | Anhui | 2013 |
| A/Anhui/1-YK_RG12/2013 | Human | Anhui | 2013 |
| A/Anhui/1-YK_RG120/2013 | Human | Anhui | 2013 |
| A/Anhui/1-YK_RG121/2013 | Human | Anhui | 2013 |
| A/Anhui/1-YK_RG122/2013 | Human | Anhui | 2013 |
| A/Anhui/1-YK_RG123/2013 | Human | Anhui | 2013 |
| A/Anhui/1-YK_RG124/2013 | Human | Anhui | 2013 |
| A/Anhui/1-YK_RG125/2013 | Human | Anhui | 2013 |
| A/Anhui/1-YK_RG126/2013 | Human | Anhui | 2013 |
| A/Anhui/1-YK_RG127/2013 | Human | Anhui | 2013 |
| A/Anhui/1-YK_RG128/2013 | Human | Anhui | 2013 |
| A/Anhui/1-YK_RG129/2013 | Human | Anhui | 2013 |
| A/Anhui/1-YK_RG13/2013 | Human | Anhui | 2013 |
| A/Anhui/1-YK_RG130/2013 | Human | Anhui | 2013 |
| A/Anhui/1-YK_RG131/2013 | Human | Anhui | 2013 |
| A/Anhui/1-YK_RG132/2013 | Human | Anhui | 2013 |
| A/Anhui/1-YK_RG133/2013 | Human | Anhui | 2013 |
| A/Anhui/1-YK_RG134/2013 | Human | Anhui | 2013 |
| A/Anhui/1-YK_RG135/2013 | Human | Anhui | 2013 |
| A/Anhui/1-YK_RG136/2013 | Human | Anhui | 2013 |
| A/Anhui/1-YK_RG137/2013 | Human | Anhui | 2013 |
| A/Anhui/1-YK_RG138/2013 | Human | Anhui | 2013 |
| A/Anhui/1-YK_RG139/2013 | Human | Anhui | 2013 |
| A/Anhui/1-YK_RG14/2013 | Human | Anhui | 2013 |
| A/Anhui/1-YK_RG140/2013 | Human | Anhui | 2013 |
| A/Anhui/1-YK_RG141/2013 | Human | Anhui | 2013 |
| A/Anhui/1-YK_RG142/2013 | Human | Anhui | 2013 |
| A/Anhui/1-YK_RG143/2013 | Human | Anhui | 2013 |
| A/Anhui/1-YK_RG144/2013 | Human | Anhui | 2013 |
| A/Anhui/1-YK_RG145/2013 | Human | Anhui | 2013 |
| A/Anhui/1-YK_RG146/2013 | Human | Anhui | 2013 |
| A/Anhui/1-YK_RG147/2013 | Human | Anhui | 2013 |
| A/Anhui/1-YK_RG148/2013 | Human | Anhui | 2013 |
| A/Anhui/1-YK_RG149/2013 | Human | Anhui | 2013 |
| A/Anhui/1-YK_RG15/2013 | Human | Anhui | 2013 |
| A/Anhui/1-YK_RG150/2013 | Human | Anhui | 2013 |
| A/Anhui/1-YK_RG151/2013 | Human | Anhui | 2013 |
| A/Anhui/1-YK_RG152/2013 | Human | Anhui | 2013 |
| A/Anhui/1-YK_RG153/2013 | Human | Anhui | 2013 |
| A/Anhui/1-YK_RG154/2013 | Human | Anhui | 2013 |
| A/Anhui/1-YK_RG155/2013 | Human | Anhui | 2013 |
| A/Anhui/1-YK_RG156/2013 | Human | Anhui | 2013 |
| A/Anhui/1-YK_RG157/2013 | Human | Anhui | 2013 |
| A/Anhui/1-YK_RG158/2013 | Human | Anhui | 2013 |
| A/Anhui/1-YK_RG159/2013 | Human | Anhui | 2013 |
| A/Anhui/1-YK_RG16/2013 | Human | Anhui | 2013 |
| A/Anhui/1-YK_RG160/2013 | Human | Anhui | 2013 |
| A/Anhui/1-YK_RG161/2013 | Human | Anhui | 2013 |
| A/Anhui/1-YK_RG162/2013 | Human | Anhui | 2013 |
| A/Anhui/1-YK_RG163/2013 | Human | Anhui | 2013 |
| A/Anhui/1-YK_RG164/2013 | Human | Anhui | 2013 |
| A/Anhui/1-YK_RG165/2013 | Human | Anhui | 2013 |
| A/Anhui/1-YK_RG166/2013 | Human | Anhui | 2013 |
| A/Anhui/1-YK_RG167/2013 | Human | Anhui | 2013 |
| A/Anhui/1-YK_RG168/2013 | Human | Anhui | 2013 |
| A/Anhui/1-YK_RG169/2013 | Human | Anhui | 2013 |
| A/Anhui/1-YK_RG17/2013 | Human | Anhui | 2013 |
| A/Anhui/1-YK_RG170/2013 | Human | Anhui | 2013 |
| A/Anhui/1-YK_RG171/2013 | Human | Anhui | 2013 |
| A/Anhui/1-YK_RG172/2013 | Human | Anhui | 2013 |
| A/Anhui/1-YK_RG173/2013 | Human | Anhui | 2013 |
| A/Anhui/1-YK_RG174/2013 | Human | Anhui | 2013 |
| A/Anhui/1-YK_RG176/2013 | Human | Anhui | 2013 |
| A/Anhui/1-YK_RG177/2013 | Human | Anhui | 2013 |
| A/Anhui/1-YK_RG178/2013 | Human | Anhui | 2013 |
| A/Anhui/1-YK_RG179/2013 | Human | Anhui | 2013 |
| A/Anhui/1-YK_RG18/2013 | Human | Anhui | 2013 |
| A/Anhui/1-YK_RG180/2013 | Human | Anhui | 2013 |
| A/Anhui/1-YK_RG181/2013 | Human | Anhui | 2013 |
| A/Anhui/1-YK_RG182/2013 | Human | Anhui | 2013 |
| A/Anhui/1-YK_RG183/2013 | Human | Anhui | 2013 |
| A/Anhui/1-YK_RG184/2013 | Human | Anhui | 2013 |
| A/Anhui/1-YK_RG185/2013 | Human | Anhui | 2013 |
| A/Anhui/1-YK_RG186/2013 | Human | Anhui | 2013 |
| A/Anhui/1-YK_RG187/2013 | Human | Anhui | 2013 |
| A/Anhui/1-YK_RG188/2013 | Human | Anhui | 2013 |
| A/Anhui/1-YK_RG189/2013 | Human | Anhui | 2013 |
| A/Anhui/1-YK_RG19/2013 | Human | Anhui | 2013 |
| A/Anhui/1-YK_RG190/2013 | Human | Anhui | 2013 |
| A/Anhui/1-YK_RG191/2013 | Human | Anhui | 2013 |
| A/Anhui/1-YK_RG192/2013 | Human | Anhui | 2013 |
| A/Anhui/1-YK_RG193/2013 | Human | Anhui | 2013 |
| A/Anhui/1-YK_RG194/2013 | Human | Anhui | 2013 |
| A/Anhui/1-YK_RG195/2013 | Human | Anhui | 2013 |
| A/Anhui/1-YK_RG196/2013 | Human | Anhui | 2013 |
| A/Anhui/1-YK_RG197/2013 | Human | Anhui | 2013 |
| A/Anhui/1-YK_RG198/2013 | Human | Anhui | 2013 |
| A/Anhui/1-YK_RG199/2013 | Human | Anhui | 2013 |
| A/Anhui/1-YK_RG20/2013 | Human | Anhui | 2013 |
| A/Anhui/1-YK_RG200/2013 | Human | Anhui | 2013 |
| A/Anhui/1-YK_RG201/2013 | Human | Anhui | 2013 |
| A/Anhui/1-YK_RG202/2013 | Human | Anhui | 2013 |
| A/Anhui/1-YK_RG21/2013 | Human | Anhui | 2013 |
| A/Anhui/1-YK_RG22/2013 | Human | Anhui | 2013 |
| A/Anhui/1-YK_RG23/2013 | Human | Anhui | 2013 |
| A/Anhui/1-YK_RG24/2013 | Human | Anhui | 2013 |
| A/Anhui/1-YK_RG25/2013 | Human | Anhui | 2013 |
| A/Anhui/1-YK_RG26/2013 | Human | Anhui | 2013 |
| A/Anhui/1-YK_RG27/2013 | Human | Anhui | 2013 |
| A/Anhui/1-YK_RG28/2013 | Human | Anhui | 2013 |
| A/Anhui/1-YK_RG29/2013 | Human | Anhui | 2013 |
| A/Anhui/1-YK_RG30/2013 | Human | Anhui | 2013 |
| A/Anhui/1-YK_RG31/2013 | Human | Anhui | 2013 |
| A/Anhui/1-YK_RG32/2013 | Human | Anhui | 2013 |
| A/Anhui/1-YK_RG33/2013 | Human | Anhui | 2013 |
| A/Anhui/1-YK_RG34/2013 | Human | Anhui | 2013 |
| A/Anhui/1-YK_RG35/2013 | Human | Anhui | 2013 |
| A/Anhui/1-YK_RG36/2013 | Human | Anhui | 2013 |
| A/Anhui/1-YK_RG37/2013 | Human | Anhui | 2013 |
| A/Anhui/1-YK_RG38/2013 | Human | Anhui | 2013 |
| A/Anhui/1-YK_RG39/2013 | Human | Anhui | 2013 |
| A/Anhui/1-YK_RG40/2013 | Human | Anhui | 2013 |
| A/Anhui/1-YK_RG41/2013 | Human | Anhui | 2013 |
| A/Anhui/1-YK_RG42/2013 | Human | Anhui | 2013 |
| A/Anhui/1-YK_RG43/2013 | Human | Anhui | 2013 |
| A/Anhui/1-YK_RG44/2013 | Human | Anhui | 2013 |
| A/Anhui/1-YK_RG45/2013 | Human | Anhui | 2013 |
| A/Anhui/1-YK_RG46/2013 | Human | Anhui | 2013 |
| A/Anhui/1-YK_RG47/2013 | Human | Anhui | 2013 |
| A/Anhui/1-YK_RG48/2013 | Human | Anhui | 2013 |
| A/Anhui/1-YK_RG49/2013 | Human | Anhui | 2013 |
| A/Anhui/1-YK_RG50/2013 | Human | Anhui | 2013 |
| A/Anhui/1-YK_RG51/2013 | Human | Anhui | 2013 |
| A/Anhui/1-YK_RG52/2013 | Human | Anhui | 2013 |
| A/Anhui/1-YK_RG53/2013 | Human | Anhui | 2013 |
| A/Anhui/1-YK_RG54/2013 | Human | Anhui | 2013 |
| A/Anhui/1-YK_RG55/2013 | Human | Anhui | 2013 |
| A/Anhui/1-YK_RG56/2013 | Human | Anhui | 2013 |
| A/Anhui/1-YK_RG57/2013 | Human | Anhui | 2013 |
| A/Anhui/1-YK_RG58/2013 | Human | Anhui | 2013 |
| A/Anhui/1-YK_RG59/2013 | Human | Anhui | 2013 |
| A/Anhui/1-YK_RG60/2013 | Human | Anhui | 2013 |
| A/Anhui/1-YK_RG61/2013 | Human | Anhui | 2013 |
| A/Anhui/1-YK_RG62/2013 | Human | Anhui | 2013 |
| A/Anhui/1-YK_RG63/2013 | Human | Anhui | 2013 |
| A/Anhui/1-YK_RG64/2013 | Human | Anhui | 2013 |
| A/Anhui/1-YK_RG65/2013 | Human | Anhui | 2013 |
| A/Anhui/1-YK_RG66/2013 | Human | Anhui | 2013 |
| A/Anhui/1-YK_RG67/2013 | Human | Anhui | 2013 |
| A/Anhui/1-YK_RG68/2013 | Human | Anhui | 2013 |
| A/Anhui/1-YK_RG69/2013 | Human | Anhui | 2013 |
| A/Anhui/1-YK_RG70/2013 | Human | Anhui | 2013 |
| A/Anhui/1-YK_RG71/2013 | Human | Anhui | 2013 |
| A/Anhui/1-YK_RG72/2013 | Human | Anhui | 2013 |
| A/Anhui/1-YK_RG73/2013 | Human | Anhui | 2013 |
| A/Anhui/1-YK_RG74/2013 | Human | Anhui | 2013 |
| A/Anhui/1-YK_RG75/2013 | Human | Anhui | 2013 |
| A/Anhui/1-YK_RG76/2013 | Human | Anhui | 2013 |
| A/Anhui/1-YK_RG77/2013 | Human | Anhui | 2013 |
| A/Anhui/1-YK_RG78/2013 | Human | Anhui | 2013 |
| A/Anhui/1-YK_RG79/2013 | Human | Anhui | 2013 |
| A/Anhui/1-YK_RG80/2013 | Human | Anhui | 2013 |
| A/Anhui/1-YK_RG81/2013 | Human | Anhui | 2013 |
| A/Anhui/1-YK_RG82/2013 | Human | Anhui | 2013 |
| A/Anhui/1-YK_RG83/2013 | Human | Anhui | 2013 |
| A/Anhui/1-YK_RG84/2013 | Human | Anhui | 2013 |
| A/Anhui/1-YK_RG85/2013 | Human | Anhui | 2013 |
| A/Anhui/1-YK_RG86/2013 | Human | Anhui | 2013 |
| A/Anhui/1-YK_RG87/2013 | Human | Anhui | 2013 |
| A/Anhui/1-YK_RG88/2013 | Human | Anhui | 2013 |
| A/Anhui/1-YK_RG89/2013 | Human | Anhui | 2013 |
| A/Anhui/1-YK_RG90/2013 | Human | Anhui | 2013 |
| A/Anhui/1-YK_RG91/2013 | Human | Anhui | 2013 |
| A/Anhui/1-YK_RG92/2013 | Human | Anhui | 2013 |
| A/Anhui/1-YK_RG93/2013 | Human | Anhui | 2013 |
| A/Anhui/1-YK_RG94/2013 | Human | Anhui | 2013 |
| A/Anhui/1-YK_RG95/2013 | Human | Anhui | 2013 |
| A/Anhui/1-YK_RG96/2013 | Human | Anhui | 2013 |
| A/Anhui/1-YK_RG97/2013 | Human | Anhui | 2013 |
| A/Anhui/1-YK_RG98/2013 | Human | Anhui | 2013 |
| A/Anhui/1-YK_RG99/2013 | Human | Anhui | 2013 |
| A/Anhui/26835/2016-03-25 | Human | Anhui | 2016/3/25 |
| A/Anhui/26837/2016-03-05 | Human | Anhui | 2016/3/5 |
| A/Anhui/29170/2017-02-28 | Human | Anhui | 2017/2/28 |
| A/Anhui/29171/2017-02-28 | Human | Anhui | 2017/2/28 |
| A/Anhui/29172/2017-03-02 | Human | Anhui | 2017/3/2 |
| A/Anhui/29173/2017-05-31 | Human | Anhui | 2017/5/31 |
| A/Anhui/29174/2017-05-26 | Human | Anhui | 2017/5/26 |
| A/Anhui/29176/2017-01-10 | Human | Anhui | 2017/1/10 |
| A/Anhui/29177/2017-01-17 | Human | Anhui | 2017/1/17 |
| A/Anhui/33223/2015-03-02 | Human | Anhui | 2015/3/2 |
| A/Anhui/33224/2015-03-05 | Human | Anhui | 2015/3/5 |
| A/Anhui/33225/2015-02-26 | Human | Anhui | 2015/2/26 |
| A/Anhui/33227/2015-02-23 | Human | Anhui | 2015/2/23 |
| A/Anhui/33228/2015-04-08 | Human | Anhui | 2015/4/8 |
| A/Anhui/40094/2015-05-23 | Human | Anhui | 2015/5/23 |
| A/Anhui/40095/2015-05-23 | Human | Anhui | 2015/5/23 |
| A/Anhui/42444/2015-06-24 | Human | Anhui | 2015/6/24 |
| A/Anhui/45322/2016-06-09 | Human | Anhui | 2016/6/9 |
| A/Anhui/60924/2016-12-22 | Human | Anhui | 2016/12/22 |
| A/Anhui/60925/2016-12-21 | Human | Anhui | 2016/12/21 |
| A/Anhui/60926/2016-12-21 | Human | Anhui | 2016/12/21 |
| A/Anhui/60927/2016-12-18 | Human | Anhui | 2016/12/18 |
| A/Anhui/60928/2016-12-15 | Human | Anhui | 2016/12/15 |
| A/Anhui/60929/2016-12-15 | Human | Anhui | 2016/12/15 |
| A/Anhui/60931/2016-12-13 | Human | Anhui | 2016/12/13 |
| A/Anhui/60932/2016-12-19 | Human | Anhui | 2016/12/19 |
| A/Anhui/60933/2016-12-19 | Human | Anhui | 2016/12/19 |
| A/Anhui/60934/2016-12-19 | Human | Anhui | 2016/12/19 |
| A/Anhui/60935/2016-12-19 | Human | Anhui | 2016/12/19 |
| A/Anhui/60936/2016-12-16 | Human | Anhui | 2016/12/16 |
| A/Anhui/DEWH72-01/2013 | Human | Anhui | 2013 |
| A/Anhui/DEWH72-02/2013 | Human | Anhui | 2013 |
| A/Anhui/DEWH72-03/2013 | Human | Anhui | 2013 |
| A/Anhui/DEWH72-04/2013 | Human | Anhui | 2013 |
| A/Anhui/DEWH72-05/2013 | Human | Anhui | 2013 |
| A/Anhui/DEWH72-06/2013 | Human | Anhui | 2013 |
| A/Anhui/DEWH72-07/2013 | Human | Anhui | 2013 |
| A/Anhui/DEWH72-08/2013 | Human | Anhui | 2013 |
| A/Anhui/DEWH72-09/2013 | Human | Anhui | 2013 |
| A/Anhui/PA-1/2013 | Human | Anhui | 2013 |
| A/Beijing/01-A/2013-04-12 | Human | Beijing | 2013/4/12 |
| A/Beijing/02/2013-05-27 | Human | Beijing | 2013/5/27 |
| A/Beijing/09149/2014-02-06 | Human | Beijing | 2014/2/6 |
| A/Beijing/1/2013/04/16 | Human | Beijing | 2013/4/16 |
| A/Beijing/10934/2017-02-14 | Human | Beijing | 2017/2/14 |
| A/Beijing/19067/2017-03-31 | Human | Beijing | 2017/3/31 |
| A/Beijing/19955/2017-04-05 | Human | Beijing | 2017/4/5 |
| A/Beijing/19957/2017-04-06 | Human | Beijing | 2017/4/6 |
| A/Beijing/2/2013/04/16 | Human | Beijing | 2013/4/16 |
| A/Beijing/21564/2017-04-15 | Human | Beijing | 2017/4/15 |
| A/Beijing/21566/2017-04-14 | Human | Beijing | 2017/4/14 |
| A/Beijing/21569/2017-04-13 | Human | Beijing | 2017/4/13 |
| A/Beijing/22136/2017-04-19 | Human | Beijing | 2017/4/19 |
| A/Beijing/22137/2017-04-17 | Human | Beijing | 2017/4/17 |
| A/Beijing/22669/2017-04-23 | Human | Beijing | 2017/4/23 |
| A/Beijing/22670/2017-04-23 | Human | Beijing | 2017/4/23 |
| A/Beijing/25686/2017-05-05 | Human | Beijing | 2017/5/5 |
| A/Beijing/25687/2017-05-09 | Human | Beijing | 2017/5/9 |
| A/Beijing/27154/2017-05-18 | Human | Beijing | 2017/5/18 |
| A/Beijing/27869/2017-05-28 | Human | Beijing | 2017/5/28 |
| A/Beijing/27870/2017-05-28 | Human | Beijing | 2017/5/28 |
| A/Beijing/28244/2017-06-05 | Human | Beijing | 2017/6/5 |
| A/Beijing/28707/2017-06-07 | Human | Beijing | 2017/6/7 |
| A/Beijing/28708/2017-06-07 | Human | Beijing | 2017/6/7 |
| A/Beijing/28933/2017-05-10 | Human | Beijing | 2017/5/10 |
| A/Beijing/29275/2017-06-16 | Human | Beijing | 2017/6/16 |
| A/Beijing/3/2013/04/16 | Human | Beijing | 2013/4/16 |
| A/Beijing/39450/2016-05-26 | Human | Beijing | 2016/5/26 |
| A/Beijing/40610/2015-05-26 | Human | Beijing | 2015/5/26 |
| A/Beijing/44066/2016-06-15 | Human | Beijing | 2016/6/15 |
| A/Beijing/46530/2016-07-26 | Human | Beijing | 2016/7/26 |
| A/Changsha/1/2013-03-22 | Human | Changsha | 2013/3/22 |
| A/Changsha/2/2013-04-29 | Human | Changsha | 2013/4/29 |
| A/Changsha/26/2017-02-04 | Human | Changsha | 2017/2/4 |
| A/Changsha/41/2017-03-12 | Human | Changsha | 2017/3/12 |
| A/Changsha/44/2017-03-14 | Human | Changsha | 2017/3/14 |
| A/Changsha/58/2017-03-17 | Human | Changsha | 2017/3/17 |
| A/Changsha/70/2017-04-13 | Human | Changsha | 2017/4/13 |
| A/chicken/Anhui/AH271/2015/03/25 | chicken | Anhui | 2015/3/25 |
| A/chicken/Anhui/AH272/2015/04/01 | chicken | Anhui | 2015/4/1 |
| A/chicken/Anhui/AH275/2015/04/01 | chicken | Anhui | 2015/4/1 |
| A/chicken/Anhui/AH284/2015/04/21 | chicken | Anhui | 2015/4/21 |
| A/chicken/Anhui/HN3/2017-07-01 | chicken | Anhui | 2017/7/1 |
| A/chicken/Anhui/HN4/2017-07-01 | chicken | Anhui | 2017/7/1 |
| A/chicken/Anhui/HN6/2017-07-01 | chicken | Anhui | 2017/7/1 |
| A/chicken/Anhui-CHuzhou/01/2013-03-29 | chicken | Anhui-CHuzhou | 2013/3/29 |
| A/chicken/China/028/2014-01-10 | chicken | China | 2014/1/10 |
| A/chicken/Dongguan/1009/2014-02-21 | chicken | Dongguan | 2014/2/21 |
| A/chicken/Dongguan/1022/2014-02-21 | chicken | Dongguan | 2014/2/21 |
| A/chicken/Dongguan/1051/2014-02-21 | chicken | Dongguan | 2014/2/21 |
| A/chicken/Dongguan/1057/2014-02-21 | chicken | Dongguan | 2014/2/21 |
| A/chicken/Dongguan/1075/2014-02-21 | chicken | Dongguan | 2014/2/21 |
| A/chicken/Dongguan/1091/2014-02-21 | chicken | Dongguan | 2014/2/21 |
| A/chicken/Dongguan/1096/2014-02-21 | chicken | Dongguan | 2014/2/21 |
| A/chicken/Dongguan/1100/2014-02-21 | chicken | Dongguan | 2014/2/21 |
| A/chicken/Dongguan/1108/2014-02-21 | chicken | Dongguan | 2014/2/21 |
| A/chicken/Dongguan/1124/2014-02-21 | chicken | Dongguan | 2014/2/21 |
| A/chicken/Dongguan/1143/2014-02-21 | chicken | Dongguan | 2014/2/21 |
| A/chicken/Dongguan/1177/2014-02-21 | chicken | Dongguan | 2014/2/21 |
| A/chicken/Dongguan/1188/2014-02-21 | chicken | Dongguan | 2014/2/21 |
| A/chicken/Dongguan/1230/2014-02-21 | chicken | Dongguan | 2014/2/21 |
| A/chicken/Dongguan/1297/2014-02-21 | chicken | Dongguan | 2014/2/21 |
| A/chicken/Dongguan/1303/2014-02-21 | chicken | Dongguan | 2014/2/21 |
| A/chicken/Dongguan/1307/2014-02-21 | chicken | Dongguan | 2014/2/21 |
| A/chicken/Dongguan/1312/2014-02-21 | chicken | Dongguan | 2014/2/21 |
| A/chicken/Dongguan/1314/2014-02-21 | chicken | Dongguan | 2014/2/21 |
| A/chicken/Dongguan/1318/2014-02-21 | chicken | Dongguan | 2014/2/21 |
| A/chicken/Dongguan/1358/2014-02-21 | chicken | Dongguan | 2014/2/21 |
| A/chicken/Dongguan/1374/2014-02-21 | chicken | Dongguan | 2014/2/21 |
| A/chicken/Dongguan/1382/2014-02-21 | chicken | Dongguan | 2014/2/21 |
| A/chicken/Dongguan/1393/2014-02-21 | chicken | Dongguan | 2014/2/21 |
| A/chicken/Dongguan/1401/2014-02-21 | chicken | Dongguan | 2014/2/21 |
| A/chicken/Dongguan/1421/2014-02-21 | chicken | Dongguan | 2014/2/21 |
| A/chicken/Dongguan/1433/2014-02-21 | chicken | Dongguan | 2014/2/21 |
| A/chicken/Dongguan/1456/2014-02-21 | chicken | Dongguan | 2014/2/21 |
| A/chicken/Dongguan/1459/2014-02-21 | chicken | Dongguan | 2014/2/21 |
| A/chicken/Dongguan/1494/2014-02-21 | chicken | Dongguan | 2014/2/21 |
| A/chicken/Dongguan/1505/2014-02-21 | chicken | Dongguan | 2014/2/21 |
| A/chicken/Dongguan/1506/2014-02-21 | chicken | Dongguan | 2014/2/21 |
| A/chicken/Dongguan/1526/2014-02-21 | chicken | Dongguan | 2014/2/21 |
| A/chicken/Dongguan/1527/2014-02-21 | chicken | Dongguan | 2014/2/21 |
| A/chicken/Dongguan/1533/2014-02-21 | chicken | Dongguan | 2014/2/21 |
| A/chicken/Dongguan/1548/2014-02-21 | chicken | Dongguan | 2014/2/21 |
| A/chicken/Dongguan/1619/2014-02-21 | chicken | Dongguan | 2014/2/21 |
| A/chicken/Dongguan/1666/2014-02-21 | chicken | Dongguan | 2014/2/21 |
| A/chicken/Dongguan/1673/2014-02-21 | chicken | Dongguan | 2014/2/21 |
| A/chicken/Dongguan/169/2014-02-20 | chicken | Dongguan | 2014/2/20 |
| A/chicken/Dongguan/1690/2014-02-21 | chicken | Dongguan | 2014/2/21 |
| A/chicken/Dongguan/1697/2014-02-21 | chicken | Dongguan | 2014/2/21 |
| A/chicken/Dongguan/173/2014-02-20 | chicken | Dongguan | 2014/2/20 |
| A/chicken/Dongguan/178/2014-02-20 | chicken | Dongguan | 2014/2/20 |
| A/chicken/Dongguan/189/2014-02-20 | chicken | Dongguan | 2014/2/20 |
| A/chicken/Dongguan/191/2014-02-20 | chicken | Dongguan | 2014/2/20 |
| A/chicken/Dongguan/210/2014-02-20 | chicken | Dongguan | 2014/2/20 |
| A/chicken/Dongguan/213/2014-02-20 | chicken | Dongguan | 2014/2/20 |
| A/chicken/Dongguan/237/2014-02-20 | chicken | Dongguan | 2014/2/20 |
| A/chicken/Dongguan/248/2014-02-20 | chicken | Dongguan | 2014/2/20 |
| A/chicken/Dongguan/262/2014-02-20 | chicken | Dongguan | 2014/2/20 |
| A/chicken/Dongguan/2912/2013-12-18 | chicken | Dongguan | 2013/12/18 |
| A/chicken/Dongguan/3112/2013-12-18 | chicken | Dongguan | 2013/12/18 |
| A/chicken/Dongguan/3141/2013-12-18 | chicken | Dongguan | 2013/12/18 |
| A/chicken/Dongguan/3145/2013-12-18 | chicken | Dongguan | 2013/12/18 |
| A/chicken/Dongguan/3146/2013-12-18 | chicken | Dongguan | 2013/12/18 |
| A/chicken/Dongguan/3219/2013-12-18 | chicken | Dongguan | 2013/12/18 |
| A/chicken/Dongguan/3418/2013-12-19 | chicken | Dongguan | 2013/12/19 |
| A/chicken/Dongguan/3438/2013-12-19 | chicken | Dongguan | 2013/12/19 |
| A/chicken/Dongguan/3464/2013-12-19 | chicken | Dongguan | 2013/12/19 |
| A/chicken/Dongguan/3487/2013-12-19 | chicken | Dongguan | 2013/12/19 |
| A/chicken/Dongguan/3488/2013-12-19 | chicken | Dongguan | 2013/12/19 |
| A/chicken/Dongguan/3491/2013-12-19 | chicken | Dongguan | 2013/12/19 |
| A/chicken/Dongguan/3544/2013-12-19 | chicken | Dongguan | 2013/12/19 |
| A/chicken/Dongguan/3563/2013-12-19 | chicken | Dongguan | 2013/12/19 |
| A/chicken/Dongguan/3582/2013-12-19 | chicken | Dongguan | 2013/12/19 |
| A/chicken/Dongguan/3894/2013-12-19 | chicken | Dongguan | 2013/12/19 |
| A/chicken/Dongguan/3917/2013-12-19 | chicken | Dongguan | 2013/12/19 |
| A/chicken/Dongguan/3935/2013-12-19 | chicken | Dongguan | 2013/12/19 |
| A/chicken/Dongguan/3945/2013-12-19 | chicken | Dongguan | 2013/12/19 |
| A/chicken/Dongguan/397/2014-02-20 | chicken | Dongguan | 2014/2/20 |
| A/chicken/Dongguan/3972/2013-12-19 | chicken | Dongguan | 2013/12/19 |
| A/chicken/Dongguan/4037/2013-12-19 | chicken | Dongguan | 2013/12/19 |
| A/chicken/Dongguan/4040/2013-12-19 | chicken | Dongguan | 2013/12/19 |
| A/chicken/Dongguan/4048/2013-12-19 | chicken | Dongguan | 2013/12/19 |
| A/chicken/Dongguan/4063/2013-12-19 | chicken | Dongguan | 2013/12/19 |
| A/chicken/Dongguan/4064/2013-12-19 | chicken | Dongguan | 2013/12/19 |
| A/chicken/Dongguan/4094/2013-12-19 | chicken | Dongguan | 2013/12/19 |
| A/chicken/Dongguan/4102/2013-12-19 | chicken | Dongguan | 2013/12/19 |
| A/chicken/Dongguan/4114/2013-12-19 | chicken | Dongguan | 2013/12/19 |
| A/chicken/Dongguan/4119/2013-12-19 | chicken | Dongguan | 2013/12/19 |
| A/chicken/Dongguan/4195/2013-12-19 | chicken | Dongguan | 2013/12/19 |
| A/chicken/Dongguan/4251/2013-12-19 | chicken | Dongguan | 2013/12/19 |
| A/chicken/Dongguan/449/2014-02-20 | chicken | Dongguan | 2014/2/20 |
| A/chicken/Dongguan/518/2014-02-20 | chicken | Dongguan | 2014/2/20 |
| A/chicken/Dongguan/536/2014-02-20 | chicken | Dongguan | 2014/2/20 |
| A/chicken/Dongguan/568/2014-02-20 | chicken | Dongguan | 2014/2/20 |
| A/chicken/Dongguan/575/2014-02-20 | chicken | Dongguan | 2014/2/20 |
| A/chicken/Dongguan/584/2014-02-20 | chicken | Dongguan | 2014/2/20 |
| A/chicken/Dongguan/695/2014-02-20 | chicken | Dongguan | 2014/2/20 |
| A/chicken/Dongguan/709/2014-02-20 | chicken | Dongguan | 2014/2/20 |
| A/chicken/Dongguan/711/2014-02-20 | chicken | Dongguan | 2014/2/20 |
| A/chicken/Dongguan/744/2014-02-20 | chicken | Dongguan | 2014/2/20 |
| A/chicken/Dongguan/748/2014-02-20 | chicken | Dongguan | 2014/2/20 |
| A/chicken/Dongguan/749/2014-02-20 | chicken | Dongguan | 2014/2/20 |
| A/chicken/Dongguan/803/2014-02-20 | chicken | Dongguan | 2014/2/20 |
| A/chicken/Dongguan/815/2014-02-20 | chicken | Dongguan | 2014/2/20 |
| A/chicken/Dongguan/835/2014-02-20 | chicken | Dongguan | 2014/2/20 |
| A/chicken/Dongguan/836/2014-02-20 | chicken | Dongguan | 2014/2/20 |
| A/chicken/Dongguan/843/2014-02-20 | chicken | Dongguan | 2014/2/20 |
| A/chicken/Dongguan/850/2014-02-20 | chicken | Dongguan | 2014/2/20 |
| A/chicken/Dongguan/851/2014-02-20 | chicken | Dongguan | 2014/2/20 |
| A/chicken/Dongguan/856/2014-02-20 | chicken | Dongguan | 2014/2/20 |
| A/chicken/Dongguan/864/2014-02-20 | chicken | Dongguan | 2014/2/20 |
| A/chicken/Dongguan/874/2014-02-20 | chicken | Dongguan | 2014/2/20 |
| A/chicken/Dongguan/899/2014-02-20 | chicken | Dongguan | 2014/2/20 |
| A/chicken/Dongguan/934/2014-02-20 | chicken | Dongguan | 2014/2/20 |
| A/chicken/Fujian/06.06_NP0001/2017-06-06 | chicken | Fujian | 2017/6/6 |
| A/chicken/Fujian/S1017/2014-03-05 | chicken | Fujian | 2014/3/5 |
| A/chicken/Fujian/S1085/2014-03-05 | chicken | Fujian | 2014/3/5 |
| A/chicken/Fujian/S1096/2015-04-08 | chicken | Fujian | 2015/4/8 |
| A/chicken/Fujian/S1322/2014-03-06 | chicken | Fujian | 2014/3/6 |
| A/chicken/Fujian/S4215/2014-11-04 | chicken | Fujian | 2014/11/4 |
| A/chicken/Fujian/S4219/2014-11-04 | chicken | Fujian | 2014/11/4 |
| A/chicken/Fujian/S4248/2014-11-04 | chicken | Fujian | 2014/11/4 |
| A/chicken/Fujian/S4518/2015-12-09 | chicken | Fujian | 2015/12/9 |
| A/chicken/Fujian/SD005/2015-01-17 | chicken | Fujian | 2015/1/17 |
| A/chicken/Ganzhou/GZ79/2016-01-27 | chicken | Ganzhou | 2016/1/27 |
| A/chicken/Guangdong/01.08_SZBJ0011-O/2017-01-08 | chicken | Guangdong | 2017/1/8 |
| A/chicken/Guangdong/12.10_DGCPLB032/2016-12-10 | chicken | Guangdong | 2016/12/10 |
| A/chicken/Guangdong/3135/2013-05-16 | chicken | Guangdong | 2013/5/16 |
| A/chicken/Guangdong/3640/2013-05-16 | chicken | Guangdong | 2013/5/16 |
| A/chicken/Guangdong/38/2014-02-10 | chicken | Guangdong | 2014/2/10 |
| A/chicken/Guangdong/53/2014-02-10 | chicken | Guangdong | 2014/2/10 |
| A/Chicken/Guangdong/CZ145/2015-02-01 | Chicken | Guangdong | 2015/2/1 |
| A/Chicken/Guangdong/DG120/2015-01-15 | Chicken | Guangdong | 2015/1/15 |
| A/Chicken/Guangdong/DG478/2014-11-25 | Chicken | Guangdong | 2014/11/25 |
| A/Chicken/Guangdong/DG479/2014-11-30 | Chicken | Guangdong | 2014/11/30 |
| A/Chicken/Guangdong/DG592/2014-12-08 | Chicken | Guangdong | 2014/12/8 |
| A/Chicken/Guangdong/DG593/2014-12-12 | Chicken | Guangdong | 2014/12/12 |
| A/chicken/Guangdong/G1/2013-05-05 | chicken | Guangdong | 2013/5/5 |
| A/chicken/Guangdong/G2/2013-05-05 | chicken | Guangdong | 2013/5/5 |
| A/chicken/Guangdong/G3/2013-05-05 | chicken | Guangdong | 2013/5/5 |
| A/chicken/Guangdong/GD15/2016/01/09 | chicken | Guangdong | 2016/1/9 |
| A/chicken/Guangdong/GD20/2017/01/06 | chicken | Guangdong | 2017/1/6 |
| A/chicken/Guangdong/GSB/2014-03-03 | chicken | Guangdong | 2014/3/3 |
| A/Chicken/Guangdong/GZ068/2015-01-01 | Chicken | Guangdong | 2015/1/1 |
| A/Chicken/Guangdong/HZ098/2015-01-01 | Chicken | Guangdong | 2015/1/1 |
| A/chicken/Guangdong/J1/2017-02 | chicken | Guangdong | Feb-17 |
| A/chicken/Guangdong/J2/2017-02 | chicken | Guangdong | Feb-17 |
| A/chicken/Guangdong/MCX/2014-04-09 | chicken | Guangdong | 2014/4/9 |
| A/chicken/Guangdong/Q1/2016/06/20 | chicken | Guangdong | 2016/6/20 |
| A/chicken/Guangdong/Q26/2017/01/12 | chicken | Guangdong | 2017/1/12 |
| A/chicken/Guangdong/Q39/2017/01/15 | chicken | Guangdong | 2017/1/15 |
| A/chicken/Guangdong/S1200/2015-03-31 | chicken | Guangdong | 2015/3/31 |
| A/chicken/Guangdong/SC086/2013-08-12 | chicken | Guangdong | 2013/8/12 |
| A/chicken/Guangdong/SC092/2013-08-12 | chicken | Guangdong | 2013/8/12 |
| A/chicken/Guangdong/SD002/2014-01-09 | chicken | Guangdong | 2014/1/9 |
| A/chicken/Guangdong/SD007/2015-01-18 | chicken | Guangdong | 2015/1/18 |
| A/chicken/Guangdong/SD008/2017-01-09 | chicken | Guangdong | 2017/1/9 |
| A/chicken/Guangdong/SD010/2017-01-18 | chicken | Guangdong | 2017/1/18 |
| A/chicken/Guangdong/SD014/2014-01-27 | chicken | Guangdong | 2014/1/27 |
| A/chicken/Guangdong/SD015/2013-12-18 | chicken | Guangdong | 2013/12/18 |
| A/chicken/Guangdong/SD018/2015-01-27 | chicken | Guangdong | 2015/1/27 |
| A/chicken/Guangdong/SD021/2014-02-23 | chicken | Guangdong | 2014/2/23 |
| A/chicken/Guangdong/SD023/2014-02-23 | chicken | Guangdong | 2014/2/23 |
| A/chicken/Guangdong/SD025/2014-03-08 | chicken | Guangdong | 2014/3/8 |
| A/chicken/Guangdong/SD027/2015-03-14 | chicken | Guangdong | 2015/3/14 |
| A/chicken/Guangdong/SD027/2017-01-18 | chicken | Guangdong | 2017/1/18 |
| A/chicken/Guangdong/SD028/2017-01-18 | chicken | Guangdong | 2017/1/18 |
| A/chicken/Guangdong/SD029/2014-03-14 | chicken | Guangdong | 2014/3/14 |
| A/chicken/Guangdong/SD031/2017-01-18 | chicken | Guangdong | 2017/1/18 |
| A/chicken/Guangdong/SD032/2017-01-12 | chicken | Guangdong | 2017/1/12 |
| A/chicken/Guangdong/SD034/2017-01-22 | chicken | Guangdong | 2017/1/22 |
| A/chicken/Guangdong/SD1/2013-05-16 | chicken | Guangdong | 2013/5/16 |
| A/chicken/Guangdong/SD1433/2016-04-06 | chicken | Guangdong | 2016/4/6 |
| A/chicken/Guangdong/SD641/2013-05-03 | chicken | Guangdong | 2013/5/3 |
| A/Chicken/Guangdong/SW153/2015-02-05 | Chicken | Guangdong | 2015/2/5 |
| A/Chicken/Guangdong/SW154/2015-02-10 | Chicken | Guangdong | 2015/2/10 |
| A/chicken/Guangxi/SD004/2014-01-24 | chicken | Guangxi | 2014/1/24 |
| A/chicken/Guangxi/SD005/2014-01-24 | chicken | Guangxi | 2014/1/24 |
| A/chicken/Guangxi/SD006/2014-01-24 | chicken | Guangxi | 2014/1/24 |
| A/chicken/Guangzhou/1/2013-07-12 | chicken | Guangzhou | 2013/7/12 |
| A/chicken/Hangzhou/174/2013-05-05 | chicken | Hangzhou | 2013/5/5 |
| A/chicken/Hangzhou/48-1/2013(H7N9)\|_\|_2013-04-10 | chicken | Hangzhou | 2013/4/10 |
| A/chicken/Hangzhou/50-1/2013(H7N9)\|_\|_2013-04-10 | chicken | Hangzhou | 2013/4/10 |
| A/chicken/Hebei/02.22_XT001/2017-02-22 | chicken | Hebei | 2017/2/22 |
| A/chicken/Hebei/HB13/2016/12/31 | chicken | Hebei | 2016/12/31 |
| A/chicken/Hebei/S1257/2016-04-27 | chicken | Hebei | 2016/4/27 |
| A/chicken/Heinan/ZZ01/2017-04-24 | chicken | Heinan | 2017/4/24 |
| A/chicken/Henan/100/2013 | chicken | Henan | 2013 |
| A/chicken/Henan/102/2013 | chicken | Henan | 2013 |
| A/chicken/Henan/103/2013 | chicken | Henan | 2013 |
| A/chicken/Henan/106/2013 | chicken | Henan | 2013 |
| A/chicken/Henan/107/2013 | chicken | Henan | 2013 |
| A/chicken/Henan/109/2013 | chicken | Henan | 2013 |
| A/chicken/Henan/112/2013 | chicken | Henan | 2013 |
| A/chicken/Henan/114/2013 | chicken | Henan | 2013 |
| A/chicken/Henan/115/2013 | chicken | Henan | 2013 |
| A/chicken/Henan/117/2013 | chicken | Henan | 2013 |
| A/chicken/Henan/120/2013 | chicken | Henan | 2013 |
| A/chicken/Henan/141/2013 | chicken | Henan | 2013 |
| A/chicken/Henan/89/2013 | chicken | Henan | 2013 |
| A/chicken/Henan/93/2013 | chicken | Henan | 2013 |
| A/chicken/Henan/96/2013 | chicken | Henan | 2013 |
| A/chicken/Henan/97/2013 | chicken | Henan | 2013 |
| A/chicken/Henan/99/2013 | chicken | Henan | 2013 |
| A/chicken/Henan/F248/2013 | chicken | Henan | 2013 |
| A/chicken/Henan/S1425/2014-06-05 | chicken | Henan | 2014/6/5 |
| A/chicken/Huaian/003/2015-02-05 | chicken | Huaian | 2015/2/5 |
| A/chicken/Huaian/007/2014-01-15 | chicken | Huaian | 2014/1/15 |
| A/chicken/Huaian/041/2014-02-04 | chicken | Huaian | 2014/2/4 |
| A/chicken/Huaian/053/2014-02-10 | chicken | Huaian | 2014/2/10 |
| A/Chicken/Huizhou/HZ-3/2016(H7N9)\|_\|_2016-12-28 | Chicken | Huizhou | 2016/12/28 |
| A/Chicken/Huizhou/HZX/2017(H7N9)\|_\|_2017-01-10 | Chicken | Huizhou | 2017/1/10 |
| A/chicken/Hunan/S12753/2016-05-19 | chicken | Hunan | 2016/5/19 |
| A/chicken/Hunan/SD010/2014-01-28 | chicken | Hunan | 2014/1/28 |
| A/chicken/Hunan/SD017/2014-02-18 | chicken | Hunan | 2014/2/18 |
| A/chicken/Hunan/SD019/2014-02-18 | chicken | Hunan | 2014/2/18 |
| A/chicken/Huzhou/3765/2013-10-24 | chicken | Huzhou | 2013/10/24 |
| A/chicken/Huzhou/3791/2013-10-24 | chicken | Huzhou | 2013/10/24 |
| A/chicken/Huzhou/3802/2013-10-24 | chicken | Huzhou | 2013/10/24 |
| A/chicken/Huzhou/4045/2013-10-24 | chicken | Huzhou | 2013/10/24 |
| A/chicken/Huzhou/4067/2013-10-24 | chicken | Huzhou | 2013/10/24 |
| A/chicken/Huzhou/4073/2013-10-24 | chicken | Huzhou | 2013/10/24 |
| A/chicken/Huzhou/4074/2013-10-24 | chicken | Huzhou | 2013/10/24 |
| A/chicken/Huzhou/4076/2013-10-24 | chicken | Huzhou | 2013/10/24 |
| A/chicken/Huzhou/4083/2013-10-24 | chicken | Huzhou | 2013/10/24 |
| A/chicken/Huzhou/4141/2013-10-24 | chicken | Huzhou | 2013/10/24 |
| A/chicken/Huzhou/4169/2013-10-24 | chicken | Huzhou | 2013/10/24 |
| A/chicken/Jiangsu/0116/2017/01/16 | chicken | Jiangsu | 2017/1/16 |
| A/chicken/Jiangsu/05.02_DT001/2017-05-02 | chicken | Jiangsu | 2017/5/2 |
| A/chicken/Jiangsu/1021/2013-04 | chicken | Jiangsu | Apr-13 |
| A/chicken/Jiangsu/12.2_KS001-O/2016-12-02 | chicken | Jiangsu | 2016/12/2 |
| A/chicken/Jiangsu/12.2_KS004-O/2016-12-02 | chicken | Jiangsu | 2016/12/2 |
| A/chicken/Jiangsu/12.2_KS008-O/2016-12-02 | chicken | Jiangsu | 2016/12/2 |
| A/chicken/Jiangsu/JS11/2016/04/29 | chicken | Jiangsu | 2016/4/29 |
| A/chicken/Jiangsu/JT114/2015/03/12 | chicken | Jiangsu | 2015/3/12 |
| A/chicken/Jiangsu/JT115/2015/03/12 | chicken | Jiangsu | 2015/3/12 |
| A/chicken/Jiangsu/JT119/2015/03/16 | chicken | Jiangsu | 2015/3/16 |
| A/chicken/Jiangsu/JT123/2015/03/16 | chicken | Jiangsu | 2015/3/16 |
| A/chicken/Jiangsu/JT156/2016/12/06 | chicken | Jiangsu | 2016/12/6 |
| A/chicken/Jiangsu/JT164/2017/01/11 | chicken | Jiangsu | 2017/1/11 |
| A/chicken/Jiangsu/JT186/2017/01/16 | chicken | Jiangsu | 2017/1/16 |
| A/chicken/Jiangsu/K27/2013-04-01 | chicken | Jiangsu | 2013/4/1 |
| A/chicken/Jiangsu/K89/2013-04-01 | chicken | Jiangsu | 2013/4/1 |
| A/chicken/Jiangsu/LY246/2017/01/19 | chicken | Jiangsu | 2017/1/19 |
| A/chicken/Jiangsu/LY79/2015/03/04 | chicken | Jiangsu | 2015/3/4 |
| A/chicken/Jiangsu/RG126/2015/03/04 | chicken | Jiangsu | 2015/3/4 |
| A/chicken/Jiangsu/S002/2013-04-16 | chicken | Jiangsu | 2013/4/16 |
| A/chicken/Jiangsu/S1045/2016-03-13 | chicken | Jiangsu | 2016/3/13 |
| A/chicken/Jiangsu/S1361/2015-04-02 | chicken | Jiangsu | 2015/4/2 |
| A/chicken/Jiangsu/S1441/2016-03-15 | chicken | Jiangsu | 2016/3/15 |
| A/chicken/Jiangsu/S1460/2016-03-15 | chicken | Jiangsu | 2016/3/15 |
| A/chicken/Jiangsu/S4519/2015-11-10 | chicken | Jiangsu | 2015/11/10 |
| A/chicken/Jiangsu/SC035/2013-04-16 | chicken | Jiangsu | 2013/4/16 |
| A/chicken/Jiangsu/SC099/2013-04-16 | chicken | Jiangsu | 2013/4/16 |
| A/chicken/Jiangsu/SC537/2013-04-16 | chicken | Jiangsu | 2013/4/16 |
| A/chicken/Jiangsu/SD012/2015-01-17 | chicken | Jiangsu | 2015/1/17 |
| A/chicken/Jiangsu/TZ45/2017/01/16 | chicken | Jiangsu | 2017/1/16 |
| A/chicken/Jiangsu/W1-8/2015-01-02 | chicken | Jiangsu | 2015/1/2 |
| A/chicken/Jiangsu/XZ250/2015/03/17 | chicken | Jiangsu | 2015/3/17 |
| A/chicken/Jiangsu/XZ252/2015/03/19 | chicken | Jiangsu | 2015/3/19 |
| A/chicken/Jiangsu/XZ256/2015/03/22 | chicken | Jiangsu | 2015/3/22 |
| A/chicken/Jiangsu/YZ30/2017/01/11 | chicken | Jiangsu | 2017/1/11 |
| A/chicken/Jiangxi/02.16_ShRGF080-P/2017-02-16 | chicken | Jiangxi | 2017/2/16 |
| A/Chicken/Jiangxi/02.17_ShRGF219-O/2017(H7N9)\|_\|_2017-02-17 | Chicken | Jiangxi | 2017/2/17 |
| A/chicken/Jiangxi/10552/2014-02-18 | chicken | Jiangxi | 2014/2/18 |
| A/chicken/Jiangxi/10573/2014-02-18 | chicken | Jiangxi | 2014/2/18 |
| A/chicken/Jiangxi/10784/2014/02/18 | chicken | Jiangxi | 2014/2/18 |
| A/chicken/Jiangxi/10870/2014-02-18 | chicken | Jiangxi | 2014/2/18 |
| A/chicken/Jiangxi/10871/2014-02-18 | chicken | Jiangxi | 2014/2/18 |
| A/chicken/Jiangxi/10873/2014-02-18 | chicken | Jiangxi | 2014/2/18 |
| A/chicken/Jiangxi/10874/2014-02-18 | chicken | Jiangxi | 2014/2/18 |
| A/chicken/Jiangxi/10875/2014-02-18 | chicken | Jiangxi | 2014/2/18 |
| A/chicken/Jiangxi/10877/2014-02-18 | chicken | Jiangxi | 2014/2/18 |
| A/chicken/Jiangxi/10882/2014-02-18 | chicken | Jiangxi | 2014/2/18 |
| A/chicken/Jiangxi/10885/2014-02-18 | chicken | Jiangxi | 2014/2/18 |
| A/chicken/Jiangxi/10894/2014-02-18 | chicken | Jiangxi | 2014/2/18 |
| A/chicken/Jiangxi/10895/2014-02-18 | chicken | Jiangxi | 2014/2/18 |
| A/chicken/Jiangxi/10896/2014-02-18 | chicken | Jiangxi | 2014/2/18 |
| A/chicken/Jiangxi/10897/2014-02-18 | chicken | Jiangxi | 2014/2/18 |
| A/chicken/Jiangxi/10929/2014-02-18 | chicken | Jiangxi | 2014/2/18 |
| A/chicken/Jiangxi/10939/2014-02-18 | chicken | Jiangxi | 2014/2/18 |
| A/chicken/Jiangxi/10943/2014-02-18 | chicken | Jiangxi | 2014/2/18 |
| A/chicken/Jiangxi/10945/2014-02-18 | chicken | Jiangxi | 2014/2/18 |
| A/chicken/Jiangxi/10946/2014-02-18 | chicken | Jiangxi | 2014/2/18 |
| A/chicken/Jiangxi/10947/2014-02-18 | chicken | Jiangxi | 2014/2/18 |
| A/chicken/Jiangxi/10948/2014-02-18 | chicken | Jiangxi | 2014/2/18 |
| A/chicken/Jiangxi/10950/2014-02-18 | chicken | Jiangxi | 2014/2/18 |
| A/chicken/Jiangxi/10953/2014-02-18 | chicken | Jiangxi | 2014/2/18 |
| A/chicken/Jiangxi/10954/2014-02-18 | chicken | Jiangxi | 2014/2/18 |
| A/chicken/Jiangxi/10955/2014-02-18 | chicken | Jiangxi | 2014/2/18 |
| A/chicken/Jiangxi/10956/2014-02-18 | chicken | Jiangxi | 2014/2/18 |
| A/chicken/Jiangxi/10957/2014-02-18 | chicken | Jiangxi | 2014/2/18 |
| A/chicken/Jiangxi/10958/2014-02-18 | chicken | Jiangxi | 2014/2/18 |
| A/chicken/Jiangxi/10959/2014-02-18 | chicken | Jiangxi | 2014/2/18 |
| A/chicken/Jiangxi/10961/2014-02-18 | chicken | Jiangxi | 2014/2/18 |
| A/chicken/Jiangxi/10962/2014-02-18 | chicken | Jiangxi | 2014/2/18 |
| A/chicken/Jiangxi/10963/2014-02-18 | chicken | Jiangxi | 2014/2/18 |
| A/chicken/Jiangxi/10964/2014-02-18 | chicken | Jiangxi | 2014/2/18 |
| A/chicken/Jiangxi/10965/2014-02-18 | chicken | Jiangxi | 2014/2/18 |
| A/chicken/Jiangxi/12200/2014-03-16 | chicken | Jiangxi | 2014/3/16 |
| A/chicken/Jiangxi/12201/2014-03-16 | chicken | Jiangxi | 2014/3/16 |
| A/chicken/Jiangxi/12206/2014-03-16 | chicken | Jiangxi | 2014/3/16 |
| A/chicken/Jiangxi/12208/2014-03-16 | chicken | Jiangxi | 2014/3/16 |
| A/chicken/Jiangxi/12210/2014-03-16 | chicken | Jiangxi | 2014/3/16 |
| A/chicken/Jiangxi/12216/2014-03-16 | chicken | Jiangxi | 2014/3/16 |
| A/chicken/Jiangxi/12217/2014-03-16 | chicken | Jiangxi | 2014/3/16 |
| A/chicken/Jiangxi/12219/2014-03-16 | chicken | Jiangxi | 2014/3/16 |
| A/chicken/Jiangxi/12221/2014-03-16 | chicken | Jiangxi | 2014/3/16 |
| A/chicken/Jiangxi/12222/2014-03-16 | chicken | Jiangxi | 2014/3/16 |
| A/chicken/Jiangxi/12223/2014-03-16 | chicken | Jiangxi | 2014/3/16 |
| A/chicken/Jiangxi/12232/2014-03-16 | chicken | Jiangxi | 2014/3/16 |
| A/chicken/Jiangxi/12239/2014-03-16 | chicken | Jiangxi | 2014/3/16 |
| A/chicken/Jiangxi/12240/2014-03-16 | chicken | Jiangxi | 2014/3/16 |
| A/chicken/Jiangxi/12243/2014-03-16 | chicken | Jiangxi | 2014/3/16 |
| A/chicken/Jiangxi/12245/2014-03-16 | chicken | Jiangxi | 2014/3/16 |
| A/chicken/Jiangxi/12247/2014-03-16 | chicken | Jiangxi | 2014/3/16 |
| A/chicken/Jiangxi/12248/2014-03-16 | chicken | Jiangxi | 2014/3/16 |
| A/chicken/Jiangxi/12249/2014-03-16 | chicken | Jiangxi | 2014/3/16 |
| A/chicken/Jiangxi/12251/2014-03-16 | chicken | Jiangxi | 2014/3/16 |
| A/chicken/Jiangxi/12254/2014-03-16 | chicken | Jiangxi | 2014/3/16 |
| A/chicken/Jiangxi/12256/2014-03-16 | chicken | Jiangxi | 2014/3/16 |
| A/chicken/Jiangxi/12260/2014-03-16 | chicken | Jiangxi | 2014/3/16 |
| A/chicken/Jiangxi/12261/2014-03-16 | chicken | Jiangxi | 2014/3/16 |
| A/chicken/Jiangxi/12264/2014-03-16 | chicken | Jiangxi | 2014/3/16 |
| A/chicken/Jiangxi/12265/2014-03-16 | chicken | Jiangxi | 2014/3/16 |
| A/chicken/Jiangxi/12273/2014-03-16 | chicken | Jiangxi | 2014/3/16 |
| A/chicken/Jiangxi/12274/2014-03-16 | chicken | Jiangxi | 2014/3/16 |
| A/chicken/Jiangxi/12486/2013-04-07 | chicken | Jiangxi | 2013/4/7 |
| A/chicken/Jiangxi/12492/2013-04-07 | chicken | Jiangxi | 2013/4/7 |
| A/chicken/Jiangxi/12544/2013-04-07 | chicken | Jiangxi | 2013/4/7 |
| A/chicken/Jiangxi/12554/2013-04-07 | chicken | Jiangxi | 2013/4/7 |
| A/chicken/Jiangxi/12564/2013-04-07 | chicken | Jiangxi | 2013/4/7 |
| A/chicken/Jiangxi/12768/2014-03-22 | chicken | Jiangxi | 2014/3/22 |
| A/chicken/Jiangxi/13207/2014-03-30 | chicken | Jiangxi | 2014/3/30 |
| A/chicken/Jiangxi/13209/2014-03-30 | chicken | Jiangxi | 2014/3/30 |
| A/chicken/Jiangxi/13210/2014-03-30 | chicken | Jiangxi | 2014/3/30 |
| A/chicken/Jiangxi/13220/2014-03-30 | chicken | Jiangxi | 2014/3/30 |
| A/chicken/Jiangxi/13223/2014-03-30 | chicken | Jiangxi | 2014/3/30 |
| A/chicken/Jiangxi/13230/2014-03-30 | chicken | Jiangxi | 2014/3/30 |
| A/chicken/Jiangxi/13250/2014-03-30 | chicken | Jiangxi | 2014/3/30 |
| A/chicken/Jiangxi/13252/2014-03-30 | chicken | Jiangxi | 2014/3/30 |
| A/chicken/Jiangxi/13255/2014-03-30 | chicken | Jiangxi | 2014/3/30 |
| A/chicken/Jiangxi/13268/2014-03-30 | chicken | Jiangxi | 2014/3/30 |
| A/chicken/Jiangxi/13269/2014-03-30 | chicken | Jiangxi | 2014/3/30 |
| A/chicken/Jiangxi/13491/2014-04-11 | chicken | Jiangxi | 2014/4/11 |
| A/chicken/Jiangxi/13493/2014-04-06 | chicken | Jiangxi | 2014/4/6 |
| A/chicken/Jiangxi/13496/2014-04-11 | chicken | Jiangxi | 2014/4/11 |
| A/chicken/Jiangxi/13502/2014-04-11 | chicken | Jiangxi | 2014/4/11 |
| A/chicken/Jiangxi/13507/2014-04-06 | chicken | Jiangxi | 2014/4/6 |
| A/chicken/Jiangxi/13510/2014-04-11 | chicken | Jiangxi | 2014/4/11 |
| A/chicken/Jiangxi/13512/2014-04-06 | chicken | Jiangxi | 2014/4/6 |
| A/chicken/Jiangxi/13513/2014-04-11 | chicken | Jiangxi | 2014/4/11 |
| A/chicken/Jiangxi/13518/2014-04-11 | chicken | Jiangxi | 2014/4/11 |
| A/chicken/Jiangxi/13519/2014-04-11 | chicken | Jiangxi | 2014/4/11 |
| A/chicken/Jiangxi/13521/2014-04-06 | chicken | Jiangxi | 2014/4/6 |
| A/chicken/Jiangxi/13524/2014-04-06 | chicken | Jiangxi | 2014/4/6 |
| A/chicken/Jiangxi/13530/2014-04-06 | chicken | Jiangxi | 2014/4/6 |
| A/chicken/Jiangxi/13536/2014-04-06 | chicken | Jiangxi | 2014/4/6 |
| A/chicken/Jiangxi/13537/2014-04-06 | chicken | Jiangxi | 2014/4/6 |
| A/chicken/Jiangxi/13538/2014-04-11 | chicken | Jiangxi | 2014/4/11 |
| A/chicken/Jiangxi/13543/2014-04-06 | chicken | Jiangxi | 2014/4/6 |
| A/chicken/Jiangxi/13544/2014-04-06 | chicken | Jiangxi | 2014/4/6 |
| A/chicken/Jiangxi/13546/2014-04-11 | chicken | Jiangxi | 2014/4/11 |
| A/chicken/Jiangxi/13548/2014-04-06 | chicken | Jiangxi | 2014/4/6 |
| A/chicken/Jiangxi/13551/2014-04-06 | chicken | Jiangxi | 2014/4/6 |
| A/chicken/Jiangxi/13553/2014-04-11 | chicken | Jiangxi | 2014/4/11 |
| A/chicken/Jiangxi/13556/2014-04-06 | chicken | Jiangxi | 2014/4/6 |
| A/chicken/Jiangxi/13564/2014-04-06 | chicken | Jiangxi | 2014/4/6 |
| A/chicken/Jiangxi/14023/2014-04-13 | chicken | Jiangxi | 2014/4/13 |
| A/chicken/Jiangxi/14033/2014-04-13 | chicken | Jiangxi | 2014/4/13 |
| A/chicken/Jiangxi/14479/2014-04-20 | chicken | Jiangxi | 2014/4/20 |
| A/chicken/Jiangxi/14482/2014-04-20 | chicken | Jiangxi | 2014/4/20 |
| A/chicken/Jiangxi/14513/2014-04-20 | chicken | Jiangxi | 2014/4/20 |
| A/chicken/Jiangxi/14515/2014-04-20 | chicken | Jiangxi | 2014/4/20 |
| A/chicken/Jiangxi/14517/2014-04-20 | chicken | Jiangxi | 2014/4/20 |
| A/chicken/Jiangxi/14518/2014-04-20 | chicken | Jiangxi | 2014/4/20 |
| A/chicken/Jiangxi/14530/2014-04-20 | chicken | Jiangxi | 2014/4/20 |
| A/chicken/Jiangxi/14554/2014-04-20 | chicken | Jiangxi | 2014/4/20 |
| A/chicken/Jiangxi/15524/2014-05-05 | chicken | Jiangxi | 2014/5/5 |
| A/chicken/Jiangxi/18008/2014-06-07 | chicken | Jiangxi | 2014/6/7 |
| A/chicken/Jiangxi/18449/2014-06-15 | chicken | Jiangxi | 2014/6/15 |
| A/chicken/Jiangxi/18482/2014-06-15 | chicken | Jiangxi | 2014/6/15 |
| A/chicken/Jiangxi/18487/2014-06-15 | chicken | Jiangxi | 2014/6/15 |
| A/chicken/Jiangxi/18513/2014-06-15 | chicken | Jiangxi | 2014/6/15 |
| A/chicken/Jiangxi/18515/2014-06-15 | chicken | Jiangxi | 2014/6/15 |
| A/chicken/Jiangxi/9497/2014-02-16 | chicken | Jiangxi | 2014/2/16 |
| A/chicken/Jiangxi/9508/2014-02-16 | chicken | Jiangxi | 2014/2/16 |
| A/chicken/Jiangxi/9513/2014-02-16 | chicken | Jiangxi | 2014/2/16 |
| A/chicken/Jiangxi/9530/2014-02-16 | chicken | Jiangxi | 2014/2/16 |
| A/chicken/Jiangxi/9534/2014-02-16 | chicken | Jiangxi | 2014/2/16 |
| A/chicken/Jiangxi/9558/2014-02-16 | chicken | Jiangxi | 2014/2/16 |
| A/chicken/Jiangxi/JX4/2017/01/21 | chicken | Jiangxi | 2017/1/21 |
| A/chicken/Jiangxi/S10017/2015-03-24 | chicken | Jiangxi | 2015/3/24 |
| A/chicken/Jiangxi/S10844/2015-03-26 | chicken | Jiangxi | 2015/3/26 |
| A/chicken/Jiangxi/S11228/2015-03-27 | chicken | Jiangxi | 2015/3/27 |
| A/chicken/Jiangxi/S40993/2015-11-27 | chicken | Jiangxi | 2015/11/27 |
| A/chicken/Jiangxi/SD001/2013-05-03 | chicken | Jiangxi | 2013/5/3 |
| A/chicken/Jiaxing/4490/2013-10-25 | chicken | Jiaxing | 2013/10/25 |
| A/Chicken/Jilin/05/2014-02-21 | Chicken | Jilin | 2014/2/21 |
| A/Chicken/Jilin/13188/2014-02-20 | Chicken | Jilin | 2014/2/20 |
| A/Chicken/Jilin/13199/2014-02-20 | Chicken | Jilin | 2014/2/20 |
| A/Chicken/Jilin/13202/2014-02-21 | Chicken | Jilin | 2014/2/21 |
| A/Chicken/Jilin/13203/2014-02-21 | Chicken | Jilin | 2014/2/21 |
| A/Chicken/Jilin/13205/2014-02-21 | Chicken | Jilin | 2014/2/21 |
| A/chicken/Jilin/SD009/2016-04-15 | chicken | Jilin | 2016/4/15 |
| A/chicken/Liaoning/03.04_LN-083/2017-03-04 | chicken | Liaoning | 2017/3/4 |
| A/chicken/Liaoning/05.12_SY059/2017-05-12 | chicken | Liaoning | 2017/5/12 |
| A/chicken/Liaoning/LN1/2016/01/09 | chicken | Liaoning | 2016/1/9 |
| A/chicken/Longquan/LQ78/2016-01-30 | chicken | Longquan | 2016/1/30 |
| A/Chicken/Nanjing/761/2013-04-09 | Chicken | Nanjing | 2013/4/9 |
| A/chicken/Ningxia/S1152/2014-05-16 | chicken | Ningxia | 2014/5/16 |
| A/chicken/Quzhou/2/2015-02-24 | chicken | Quzhou | 2015/2/24 |
| A/chicken/Rizhao/515/2013-04 | chicken | Rizhao | Apr-13 |
| A/chicken/Rizhao/713/2013-04 | chicken | Rizhao | Apr-13 |
| A/chicken/Rizhao/715/2013-04 | chicken | Rizhao | Apr-13 |
| A/chicken/Rizhao/719b/2013-04 | chicken | Rizhao | Apr-13 |
| A/chicken/Rizhao/865/2013-04 | chicken | Rizhao | Apr-13 |
| A/chicken/Rizhao/867/2013-04 | chicken | Rizhao | Apr-13 |
| A/chicken/Rizhao/871/2013-04 | chicken | Rizhao | Apr-13 |
| A/chicken/Rizhao/875/2013-04 | chicken | Rizhao | Apr-13 |
| A/chicken/Shandong/02.10_QD2-758/2017-02-10 | chicken | Shandong | 2017/2/10 |
| A/chicken/Shandong/04.03_QD4-739/2017-04-03 | chicken | Shandong | 2017/4/3 |
| A/chicken/Shandong/05.05_DZ056/2017-05-05 | chicken | Shandong | 2017/5/5 |
| A/chicken/Shandong/SD183/2016/03/23 | chicken | Shandong | 2016/3/23 |
| A/chicken/Shandong/SD216/2016/12/14 | chicken | Shandong | 2016/12/14 |
| A/chicken/Shandong/SD69/2015/03/16 | chicken | Shandong | 2015/3/16 |
| A/chicken/Shanghai/017/2013-04 | chicken | Shanghai | Apr-13 |
| A/chicken/Shanghai/019/2013-04 | chicken | Shanghai | Apr-13 |
| A/chicken/Shanghai/PD-CN-02/2014-01-21 | chicken | Shanghai | 2014/1/21 |
| A/Chicken/Shanghai/S1053/2013-04-03 | Chicken | Shanghai | 2013/4/3 |
| A/chicken/Shanghai/S1055/2013-04-03 | chicken | Shanghai | 2013/4/3 |
| A/chicken/Shanghai/S1076/2013-04-03 | chicken | Shanghai | 2013/4/3 |
| A/chicken/Shanghai/S1077/2013-04-03 | chicken | Shanghai | 2013/4/3 |
| A/chicken/Shanghai/S1078/2013-04-03 | chicken | Shanghai | 2013/4/3 |
| A/chicken/Shanghai/S1079/2013-04-03 | chicken | Shanghai | 2013/4/3 |
| A/chicken/Shanghai/S1080/2013-04-03 | chicken | Shanghai | 2013/4/3 |
| A/chicken/Shanghai/S1358/2013-04-03 | chicken | Shanghai | 2013/4/3 |
| A/chicken/Shanghai/S1410/2013-04-03 | chicken | Shanghai | 2013/4/3 |
| A/chicken/Shanghai/S1413/2013-04-03 | chicken | Shanghai | 2013/4/3 |
| A/chicken/Shanghai/S4100/2015-11-11 | chicken | Shanghai | 2015/11/11 |
| A/chicken/Shantou/1550/2014-03-05 | chicken | Shantou | 2014/3/5 |
| A/chicken/Shantou/1552/2014-03-05 | chicken | Shantou | 2014/3/5 |
| A/chicken/Shantou/1554/2014-03-05 | chicken | Shantou | 2014/3/5 |
| A/chicken/Shantou/1556/2014-03-05 | chicken | Shantou | 2014/3/5 |
| A/chicken/Shantou/2537/2014-04-16 | chicken | Shantou | 2014/4/16 |
| A/chicken/Shantou/2538/2014-04-16 | chicken | Shantou | 2014/4/16 |
| A/chicken/Shantou/2539/2014-04-16 | chicken | Shantou | 2014/4/16 |
| A/chicken/Shantou/2546/2014-04-16 | chicken | Shantou | 2014/4/16 |
| A/chicken/Shantou/2550/2014-04-16 | chicken | Shantou | 2014/4/16 |
| A/chicken/Shantou/2556/2014-04-16 | chicken | Shantou | 2014/4/16 |
| A/chicken/Shantou/2562/2014-04-16 | chicken | Shantou | 2014/4/16 |
| A/chicken/Shantou/3057/2014-05-06 | chicken | Shantou | 2014/5/6 |
| A/chicken/Shantou/4325/2014-07-01 | chicken | Shantou | 2014/7/1 |
| A/chicken/Shantou/4816/2014-07-22 | chicken | Shantou | 2014/7/22 |
| A/chicken/Shantou/4824/2014-07-22 | chicken | Shantou | 2014/7/22 |
| A/chicken/Shantou/4832/2014-07-22 | chicken | Shantou | 2014/7/22 |
| A/chicken/Shantou/4833/2014-07-22 | chicken | Shantou | 2014/7/22 |
| A/chicken/Shaoxing/2417/2013-10-20 | chicken | Shaoxing | 2013/10/20 |
| A/chicken/Shaoxing/5086/2013-10-28 | chicken | Shaoxing | 2013/10/28 |
| A/chicken/Shaoxing/5087/2013-10-28 | chicken | Shaoxing | 2013/10/28 |
| A/chicken/Shaoxing/5136/2013-10-28 | chicken | Shaoxing | 2013/10/28 |
| A/chicken/Shaoxing/5146/2013-10-28 | chicken | Shaoxing | 2013/10/28 |
| A/chicken/Shaoxing/5186/2013-10-28 | chicken | Shaoxing | 2013/10/28 |
| A/chicken/Shaoxing/5201/2013-10-28 | chicken | Shaoxing | 2013/10/28 |
| A/chicken/Shaoxing/5224/2013-10-28 | chicken | Shaoxing | 2013/10/28 |
| A/chicken/Shaoxing/5227/2013-10-28 | chicken | Shaoxing | 2013/10/28 |
| A/chicken/Shaoxing/5479/2013-10-29 | chicken | Shaoxing | 2013/10/29 |
| A/chicken/Shenzhen/138/2014-02-19 | chicken | Shenzhen | 2014/2/19 |
| A/chicken/Shenzhen/1665/2013-12-12 | chicken | Shenzhen | 2013/12/12 |
| A/chicken/Shenzhen/2110/2013-12-13 | chicken | Shenzhen | 2013/12/13 |
| A/chicken/Shenzhen/2201/2013-12-13 | chicken | Shenzhen | 2013/12/13 |
| A/chicken/Shenzhen/2293/2013-12-13 | chicken | Shenzhen | 2013/12/13 |
| A/chicken/Shenzhen/3733/2013-12-19 | chicken | Shenzhen | 2013/12/19 |
| A/chicken/Shenzhen/3734/2013-12-19 | chicken | Shenzhen | 2013/12/19 |
| A/chicken/Shenzhen/3780/2013-12-19 | chicken | Shenzhen | 2013/12/19 |
| A/chicken/Shenzhen/727/2013-12-10 | chicken | Shenzhen | 2013/12/10 |
| A/chicken/Shenzhen/742/2013-12-10 | chicken | Shenzhen | 2013/12/10 |
| A/chicken/Shenzhen/747/2013-12-10 | chicken | Shenzhen | 2013/12/10 |
| A/chicken/Shenzhen/749/2013-12-10 | chicken | Shenzhen | 2013/12/10 |
| A/chicken/Shenzhen/801/2013-12-09 | chicken | Shenzhen | 2013/12/9 |
| A/chicken/Shenzhen/898/2013-12-09 | chicken | Shenzhen | 2013/12/9 |
| A/chicken/Suzhou/04020102/2013-04-02 | chicken | Suzhou | 2013/4/2 |
| A/chicken/Suzhou/040201H/2013-04 | chicken | Suzhou | Apr-13 |
| A/chicken/Suzhou/040207GH05/2013-04 | chicken | Suzhou | Apr-13 |
| A/Chicken/Suzhou/097-1/2013-04-09 | Chicken | Suzhou | 2013/4/9 |
| A/chicken/Taizhou/TZJF02/2015-01 | chicken | Taizhou | Jan-15 |
| A/chicken/Wenzhou/HATSLG01/2015-01 | chicken | Wenzhou | Jan-15 |
| A/chicken/Wenzhou/RAQL01/2015-01 | chicken | Wenzhou | Jan-15 |
| A/chicken/Wenzhou/RAQL18/2015-01 | chicken | Wenzhou | Jan-15 |
| A/chicken/Wenzhou/WZTSLG02/2015-01 | chicken | Wenzhou | Jan-15 |
| A/chicken/Wuxi/04030201/2013-04-03 | chicken | Wuxi | 2013/4/3 |
| A/chicken/Wuxi/04030201G/2013-04 | chicken | Wuxi | Apr-13 |
| A/chicken/Wuxi/0405005/2013-04-05 | chicken | Wuxi | 2013/4/5 |
| A/chicken/Wuxi/0405005G/2013-04 | chicken | Wuxi | Apr-13 |
| A/chicken/Wuxi/5852/2015-05-19 | chicken | Wuxi | 2015/5/19 |
| A/chicken/Wuxi/7144/2015-12-19 | chicken | Wuxi | 2015/12/19 |
| A/chicken/Wuxi/8048/2016-04-21 | chicken | Wuxi | 2016/4/21 |
| A/chicken/Wuxi/WX5/2014-06-01 | chicken | Wuxi | 2014/6/1 |
| A/chicken/Xinjiang/SD033/2014-12-03 | chicken | Xinjiang | 2014/12/3 |
| A/chicken/Xinjiang/SD036/2014-12-03 | chicken | Xinjiang | 2014/12/3 |
| A/chicken/ZHangzhou/8585/2014-06-23 | chicken | ZHangzhou | 2014/6/23 |
| A/chicken/ZHangzhou/8829/2014-06-30 | chicken | ZHangzhou | 2014/6/30 |
| A/chicken/Zhejiang/1.10_HZ142/2017-01-10 | chicken | Zhejiang | 2017/1/10 |
| A/chicken/Zhejiang/12.15_HZ293/2016-12-15 | chicken | Zhejiang | 2016/12/15 |
| A/chicken/Zhejiang/12.15_HZ298/2016-12-15 | chicken | Zhejiang | 2016/12/15 |
| A/chicken/Zhejiang/C481/2013-04 | chicken | Zhejiang | Apr-13 |
| A/chicken/Zhejiang/C483/2013-04 | chicken | Zhejiang | Apr-13 |
| A/chicken/Zhejiang/DTID-ZJU01/2013-04 | chicken | Zhejiang | Apr-13 |
| A/chicken/Zhejiang/DTID-ZJU06/2013-12 | chicken | Zhejiang | Dec-13 |
| A/chicken/Zhejiang/JH16/2017/02/13 | chicken | Zhejiang | 2017/2/13 |
| A/chicken/Zhejiang/JX148/2014/04 | chicken | Zhejiang | Apr-14 |
| A/chicken/Zhejiang/JX148/2014-04-05 | chicken | Zhejiang | 2014/4/5 |
| A/chicken/Zhejiang/JX158/2015/05/04 | chicken | Zhejiang | 2015/5/4 |
| A/chicken/Zhejiang/PA-DTID-ZJU01/2013 | chicken | Zhejiang | 2013 |
| A/chicken/Zhejiang/S01/2014-02-16 | chicken | Zhejiang | 2014/2/16 |
| A/chicken/Zhejiang/S1074/2016-05-10 | chicken | Zhejiang | 2016/5/10 |
| A/chicken/Zhejiang/S1190/2015-03-24 | chicken | Zhejiang | 2015/3/24 |
| A/chicken/Zhejiang/S4008/2013-12-08 | chicken | Zhejiang | 2013/12/8 |
| A/chicken/Zhejiang/S4017/2013-12-08 | chicken | Zhejiang | 2013/12/8 |
| A/chicken/Zhejiang/S4060/2014-11-25 | chicken | Zhejiang | 2014/11/25 |
| A/chicken/Zhejiang/S4071/2013-12-08 | chicken | Zhejiang | 2013/12/8 |
| A/chicken/Zhejiang/S4079/2013-12-08 | chicken | Zhejiang | 2013/12/8 |
| A/chicken/Zhejiang/S4135/2013-12-08 | chicken | Zhejiang | 2013/12/8 |
| A/chicken/Zhejiang/S4168/2013-12-08 | chicken | Zhejiang | 2013/12/8 |
| A/chicken/Zhejiang/SD001/2016-01-01 | chicken | Zhejiang | 2016/1/1 |
| A/chicken/Zhejiang/SD007/2013-04-22 | chicken | Zhejiang | 2013/4/22 |
| A/chicken/Zhejiang/SD019/2013-04-11 | chicken | Zhejiang | 2013/4/11 |
| A/chicken/Zhejiang/SD033/2013-04-11 | chicken | Zhejiang | 2013/4/11 |
| A/chicken/Zhejiang/ZJ14/2017/01/09 | chicken | Zhejiang | 2017/1/9 |
| A/chicken/Zhejiang/ZJ19/2017/01/06 | chicken | Zhejiang | 2017/1/6 |
| A/Chongqing-dianjiang/61/2017-05-01 | Human | Chongqing-dianjiang | 2017/5/1 |
| A/Chongqing-Fengjie/51/2017-03-04 | Human | Chongqing-Fengjie | 2017/3/4 |
| A/Chongqing-jiangbei/61/2017-04-25 | Human | Chongqing-jiangbei | 2017/4/25 |
| A/Chongqing-Kaixian/61/2017-06-03 | Human | Chongqing-Kaixian | 2017/6/3 |
| A/Chongqing-shapingba/61/2017-05-15 | Human | Chongqing-shapingba | 2017/5/15 |
| A/Chongqing-Shapingba/62/2017-06-06 | Human | Chongqing-Shapingba | 2017/6/6 |
| A/Chongqing-Wanzhou/67/2017-03-13 | Human | Chongqing-Wanzhou | 2017/3/13 |
| A/Chongqing-Yuzhong/1500/2017-03-22 | Human | Chongqing-Yuzhong | 2017/3/22 |
| A/Dressed_chicken/Guangdong/GZ631/2017-04-13 | Dressed_chicken | Guangdong | 2017/4/13 |
| A/duck/Anhui/S702/2013-04-04 | duck | Anhui | 2013/4/4 |
| A/duck/Anhui/SC702/2013-04-16 | duck | Anhui | 2013/4/16 |
| A/duck/Fujian/S4170/2014-11-04 | duck | Fujian | 2014/11/4 |
| A/Duck/Guangdong/DG103/2015-01-07 | Duck | Guangdong | 2015/1/7 |
| A/Duck/Guangdong/DG527/2014-12-02 | Duck | Guangdong | 2014/12/2 |
| A/duck/Guangxi/SDY129/2014-10-23 | duck | Guangxi | 2014/10/23 |
| A/duck/Hunan/S11682/2015-03-17 | duck | Hunan | 2015/3/17 |
| A/duck/Hunan/S40506/2015-11-16 | duck | Hunan | 2015/11/16 |
| A/duck/Hunan/S40685/2015-11-17 | duck | Hunan | 2015/11/17 |
| A/duck/Jiangsu/S1181/2014-05-14 | duck | Jiangsu | 2014/5/14 |
| A/duck/Jiangsu/S1220/2016-05-14 | duck | Jiangsu | 2016/5/14 |
| A/duck/Jiangsu/S1700/2016-03-15 | duck | Jiangsu | 2016/3/15 |
| A/duck/Jiangxi/02.16_ShRSX002-O/2017-02-16 | duck | Jiangxi | 2017/2/16 |
| A/duck/Jiangxi/15044/2014-04-27 | duck | Jiangxi | 2014/4/27 |
| A/duck/Jiangxi/3096/2009 | duck | Jiangxi | 2009 |
| A/duck/Jiangxi/3141/2009 | duck | Jiangxi | 2009 |
| A/duck/Jiangxi/3152/2009 | duck | Jiangxi | 2009 |
| A/duck/Jiangxi/3169/2009 | duck | Jiangxi | 2009 |
| A/duck/Jiangxi/3190/2009 | duck | Jiangxi | 2009 |
| A/duck/Jiangxi/3214/2009 | duck | Jiangxi | 2009 |
| A/duck/Jiangxi/3230/2009 | duck | Jiangxi | 2009 |
| A/duck/Jiangxi/3257/2009 | duck | Jiangxi | 2009 |
| A/duck/Jiangxi/3283/2009 | duck | Jiangxi | 2009 |
| A/duck/Jiangxi/3286/2009 | duck | Jiangxi | 2009 |
| A/duck/Jiangxi/3292/2009 | duck | Jiangxi | 2009 |
| A/duck/Shanghai/SD015/2015-01-17 | duck | Shanghai | 2015/1/17 |
| A/duck/Shanghai/SD016/2015-01-17 | duck | Shanghai | 2015/1/17 |
| A/duck/Sunan/040802G/2013-04 | duck | Sunan | Apr-13 |
| A/duck/Wenzhou/RAQL10/2015-01 | duck | Wenzhou | Jan-15 |
| A/duck/Wenzhou/YJYF24/2015-01 | duck | Wenzhou | Jan-15 |
| A/duck/Wuxi/04030204GH/2013-04 | duck | Wuxi | Apr-13 |
| A/duck/Wuxi/0405006/2013-04-05 | duck | Wuxi | 2013/4/5 |
| A/duck/Wuxi/0405006G/2013-04 | duck | Wuxi | Apr-13 |
| A/duck/Zhejiang/LS02/2014-01-12 | duck | Zhejiang | 2014/1/12 |
| A/duck/Zhejiang/S1375/2016-05-12 | duck | Zhejiang | 2016/5/12 |
| A/duck/Zhejiang/S4488/2014-11-27 | duck | Zhejiang | 2014/11/27 |
| A/duck/Zhejiang/S4489/2014-11-27 | duck | Zhejiang | 2014/11/27 |
| A/duck/Zhejiang/SC410/2013-04-16 | duck | Zhejiang | 2013/4/16 |
| A/Enviroment/Xuzhou/4030/2018-01-29 | Enviroment | Xuzhou | 2018/1/29 |
| A/Environment/Anhui/33240/2015-03-06 | Environment | Anhui | 2015/3/6 |
| A/Environment/Anhui/33248/2015-03-06 | Environment | Anhui | 2015/3/6 |
| A/Environment/Anhui/33250/2015-03-06 | Environment | Anhui | 2015/3/6 |
| A/Environment/Anhui/39280/2015-05-01 | Environment | Anhui | 2015/5/1 |
| A/Environment/Beijing/22166/2017-04-06 | Environment | Beijing | 2017/4/6 |
| A/Environment/Changzhou/022/2018-05-03 | Environment | Changzhou | 2018/5/3 |
| A/environment/ChongqingHC/2-20/2017-02-12 | environment | ChongqingHC | 2017/2/12 |
| A/Environment/Fujian/23611/2014-12-30 | Environment | Fujian | 2014/12/30 |
| A/Environment/Fujian/40791/2017-10-18 | Environment | Fujian | 2017/10/18 |
| A/Environment/Fujian/40843/2017-10-24 | Environment | Fujian | 2017/10/24 |
| A/Environment/Fujian/40844/2017-10-24 | Environment | Fujian | 2017/10/24 |
| A/Environment/Fujian/41640/2015-06-24 | Environment | Fujian | 2015/6/24 |
| A/Environment/Fujian/41642/2015-05-06 | Environment | Fujian | 2015/5/6 |
| A/Environment/Fujian/41646/2015-05-06 | Environment | Fujian | 2015/5/6 |
| A/Environment/Fujian/43639/2017-12-05 | Environment | Fujian | 2017/12/5 |
| A/environment/Fujian/SC337/2013-04-30 | environment | Fujian | 2013/4/30 |
| A/environment/Fujian/SD003/2014-01-14 | environment | Fujian | 2014/1/14 |
| A/environment/Guangdong/02621/2013-12-25 | environment | Guangdong | 2013/12/25 |
| A/environment/Guangdong/02622/2013-12-25 | environment | Guangdong | 2013/12/25 |
| A/Environment/Guangdong/02735/2016-12-26 | Environment | Guangdong | 2016/12/26 |
| A/Environment/Guangdong/02736/2016-12-26 | Environment | Guangdong | 2016/12/26 |
| A/Environment/Guangdong/02737/2016-12-26 | Environment | Guangdong | 2016/12/26 |
| A/Environment/Guangdong/02738/2016-12-26 | Environment | Guangdong | 2016/12/26 |
| A/Environment/Guangdong/02739/2016-12-26 | Environment | Guangdong | 2016/12/26 |
| A/Environment/Guangdong/02741/2016-12-26 | Environment | Guangdong | 2016/12/26 |
| A/environment/Guangdong/03.13_SZBA-E1/2017-03-13 | environment | Guangdong | 2017/3/13 |
| A/Environment/Guangdong/07103/2017-01-03 | Environment | Guangdong | 2017/1/3 |
| A/Environment/Guangdong/07104/2017-01-03 | Environment | Guangdong | 2017/1/3 |
| A/Environment/Guangdong/07125/2017-01-04 | Environment | Guangdong | 2017/1/4 |
| A/Environment/Guangdong/07128/2017-01-09 | Environment | Guangdong | 2017/1/9 |
| A/Environment/Guangdong/13921/2017-01-04 | Environment | Guangdong | 2017/1/4 |
| A/Environment/Guangdong/15120/2016-01-08 | Environment | Guangdong | 2016/1/8 |
| A/Environment/Guangdong/15123/2016-01-08 | Environment | Guangdong | 2016/1/8 |
| A/Environment/Guangdong/15125/2016-01-08 | Environment | Guangdong | 2016/1/8 |
| A/Environment/Guangdong/16609/2017-02-22 | Environment | Guangdong | 2017/2/22 |
| A/Environment/Guangdong/16661/2017-02-24 | Environment | Guangdong | 2017/2/24 |
| A/Environment/Guangdong/21123/2015-01-20 | Environment | Guangdong | 2015/1/20 |
| A/Environment/Guangdong/21231/2015-02-03 | Environment | Guangdong | 2015/2/3 |
| A/Environment/Guangdong/21275/2015-01-28 | Environment | Guangdong | 2015/1/28 |
| A/Environment/Guangdong/21286/2015-01-15 | Environment | Guangdong | 2015/1/15 |
| A/Environment/Guangdong/22351/2015-01-11 | Environment | Guangdong | 2015/1/11 |
| A/environment/Guangdong/25/2013-04-26 | environment | Guangdong | 2013/4/26 |
| A/environment/Guangdong/25003/2013-12-16 | environment | Guangdong | 2013/12/16 |
| A/environment/Guangdong/30/2013-04-26 | environment | Guangdong | 2013/4/26 |
| A/Environment/Guangdong/43966/2016-05-30 | Environment | Guangdong | 2016/5/30 |
| A/Environment/Guangdong/47403/2014-05-21 | Environment | Guangdong | 2014/5/21 |
| A/Environment/Guangdong/C13281025/2013-04-26 | Environment | Guangdong | 2013/4/26 |
| A/Environment/Guangdong/C13281030/2013-04-26 | Environment | Guangdong | 2013/4/26 |
| A/Environment/Guangdong/C16283222/2016-11-07 | Environment | Guangdong | 2016/11/7 |
| A/Environment/Guangdong/DG104/2015-01-16 | Environment | Guangdong | 2015/1/16 |
| A/Environment/Guangdong/DG127/2015-02-20 | Environment | Guangdong | 2015/2/20 |
| A/Environment/Guangdong/GZ4753/2017-02-15 | Environment | Guangdong | 2017/2/15 |
| A/Environment/Guangdong/XN01070/2013-05-13 | Environment | Guangdong | 2013/5/13 |
| A/Environment/Guangxi/06891/2014-12-23 | Environment | Guangxi | 2014/12/23 |
| A/Environment/Guangxi/23659/2017-04-07 | Environment | Guangxi | 2017/4/7 |
| A/Environment/Guangxi/24062/2017-03-09 | Environment | Guangxi | 2017/3/9 |
| A/Environment/Guangxi/24092/2017-03-14 | Environment | Guangxi | 2017/3/14 |
| A/Environment/Guangxi/24097/2017-03-14 | Environment | Guangxi | 2017/3/14 |
| A/environment/Guangzhou/1/2014-01-26 | environment | Guangzhou | 2014/1/26 |
| A/Environment/Guangzhou/4188/2016-12-21 | Environment | Guangzhou | 2016/12/21 |
| A/Environment/Guangzhou/5258-5259/2017-04-13 | Environment | Guangzhou | 2017/4/13 |
| A/Environment/Guangzhou/K4048/2016-12-15 | Environment | Guangzhou | 2016/12/15 |
| A/Environment/Guangzhou/K4068/2016-12-15 | Environment | Guangzhou | 2016/12/15 |
| A/Environment/Guangzhou/K4072/2016-12-15 | Environment | Guangzhou | 2016/12/15 |
| A/Environment/Guangzhou/K4073/2016-12-15 | Environment | Guangzhou | 2016/12/15 |
| A/Environment/Guangzhou/K4075/2016-12-15 | Environment | Guangzhou | 2016/12/15 |
| A/Environment/Guangzhou/K4756-K4776/2017-02-15 | Environment | Guangzhou | 2017/2/15 |
| A/Environment/Guangzhou/K4777-K4882/2017-02-15 | Environment | Guangzhou | 2017/2/15 |
| A/environment/Guangzhou/XN07390/2014-02-13 | environment | Guangzhou | 2014/2/13 |
| A/environment/Guangzhou/XN07874/2014-02-15 | environment | Guangzhou | 2014/2/15 |
| A/environment/Guangzhou/XN12917/2014-03-17 | environment | Guangzhou | 2014/3/17 |
| A/environment/Hangzhou/109-1/2013-04-12 | environment | Hangzhou | 2013/4/12 |
| A/environment/Hangzhou/34-1/2013-04-04 | environment | Hangzhou | 2013/4/4 |
| A/environment/Hangzhou/37/2013-04-04 | environment | Hangzhou | 2013/4/4 |
| A/environment/Hebei/1/2013-07-20 | environment | Hebei | 2013/7/20 |
| A/Environment/Hebei/22052/2017-04-17 | Environment | Hebei | 2017/4/17 |
| A/Environment/Hebei/25346/2017-04-26 | Environment | Hebei | 2017/4/26 |
| A/Environment/Hebei/27418/2017-05-06 | Environment | Hebei | 2017/5/6 |
| A/Environment/Hebei/27423/2017-05-10 | Environment | Hebei | 2017/5/10 |
| A/Environment/Hebei/27426/2017-05-01 | Environment | Hebei | 2017/5/1 |
| A/Environment/Hebei/27444/2017-05-17 | Environment | Hebei | 2017/5/17 |
| A/Environment/Hebei/27453/2017-05-19 | Environment | Hebei | 2017/5/19 |
| A/Environment/Hebei/40051/2016-05-20 | Environment | Hebei | 2016/5/20 |
| A/Environment/Hebei/40052/2016-05-20 | Environment | Hebei | 2016/5/20 |
| A/Environment/Hebei/40053/2016-05-20 | Environment | Hebei | 2016/5/20 |
| A/environment/Hebei/SC069/2013-07-22 | environment | Hebei | 2013/7/22 |
| A/environment/Henan/SC232/2013-04-24 | environment | Henan | 2013/4/24 |
| A/environment/Henan/SD429/2013-04-24 | environment | Henan | 2013/4/24 |
| A/Environment/Hubei/10497/2017-01-20 | Environment | Hubei | 2017/1/20 |
| A/Environment/Hubei/10813/2017-02-05 | Environment | Hubei | 2017/2/5 |
| A/Environment/Hubei/12133/2017-02-16 | Environment | Hubei | 2017/2/16 |
| A/Environment/Hubei/12134/2017-02-16 | Environment | Hubei | 2017/2/16 |
| A/Environment/Hubei/12136/2017-02-16 | Environment | Hubei | 2017/2/16 |
| A/Environment/Hubei/12137/2017-02-16 | Environment | Hubei | 2017/2/16 |
| A/Environment/Hubei/12167/2017-02-18 | Environment | Hubei | 2017/2/18 |
| A/Environment/Hubei/12176/2017-02-20 | Environment | Hubei | 2017/2/20 |
| A/Environment/Hubei/12177/2017-02-20 | Environment | Hubei | 2017/2/20 |
| A/Environment/Hubei/12178/2017-02-20 | Environment | Hubei | 2017/2/20 |
| A/Environment/Hunan/07002/2017-01-09 | Environment | Hunan | 2017/1/9 |
| A/Environment/Hunan/07626/2015-01-20 | Environment | Hunan | 2015/1/20 |
| A/Environment/Hunan/07836/2014-01-27 | Environment | Hunan | 2014/1/27 |
| A/Environment/Hunan/08069/2017-01-17 | Environment | Hunan | 2017/1/17 |
| A/Environment/Hunan/08070/2017-01-17 | Environment | Hunan | 2017/1/17 |
| A/Environment/Hunan/08071/2017-01-17 | Environment | Hunan | 2017/1/17 |
| A/Environment/Hunan/09790/2017-01-18 | Environment | Hunan | 2017/1/18 |
| A/Environment/Hunan/10086/2016-01-12 | Environment | Hunan | 2016/1/12 |
| A/Environment/Hunan/10125/2015-12-09 | Environment | Hunan | 2015/12/9 |
| A/Environment/Hunan/20986/2015-02-02 | Environment | Hunan | 2015/2/2 |
| A/Environment/Hunan/39719/2015-04-28 | Environment | Hunan | 2015/4/28 |
| A/Environment/Hunan/51122/2016-02-02 | Environment | Hunan | 2016/2/2 |
| A/environment/Hunan/S40858/2015-11-18 | environment | Hunan | 2015/11/18 |
| A/environment/Hunan/SD009/2015-01-16 | environment | Hunan | 2015/1/16 |
| A/Environment/Inner_Mongolia/28651/2017-06-02 | Environment | Inner_Mongolia | 2017/6/2 |
| A/Environment/Inner_Mongolia/28664/2017-05-26 | Environment | Inner_Mongolia | 2017/5/26 |
| A/Environment/Inner_Mongolia/28665/2017-05-26 | Environment | Inner_Mongolia | 2017/5/26 |
| A/Environment/Inner_Mongolia/28666/2017-05-26 | Environment | Inner_Mongolia | 2017/5/26 |
| A/Environment/Inner_Mongolia/28667/2017-05-26 | Environment | Inner_Mongolia | 2017/5/26 |
| A/Environment/Inner_Mongolia/28668/2017-05-26 | Environment | Inner_Mongolia | 2017/5/26 |
| A/Environment/Inner_Mongolia/28669/2017-05-26 | Environment | Inner_Mongolia | 2017/5/26 |
| A/Environment/Inner_Mongolia/28670/2017-05-26 | Environment | Inner_Mongolia | 2017/5/26 |
| A/environment/Jiangsu/03137/2013-12-29 | environment | Jiangsu | 2013/12/29 |
| A/environment/Jiangsu/03174/2013-12-13 | environment | Jiangsu | 2013/12/13 |
| A/Environment/Jiangsu/03758/2015-12-14 | Environment | Jiangsu | 2015/12/14 |
| A/Environment/Jiangsu/06661/2016-12-17 | Environment | Jiangsu | 2016/12/17 |
| A/Environment/Jiangsu/06662/2016-12-12 | Environment | Jiangsu | 2016/12/12 |
| A/Environment/Jiangsu/06675/2016-12-21 | Environment | Jiangsu | 2016/12/21 |
| A/Environment/Jiangsu/06690/2016-12-21 | Environment | Jiangsu | 2016/12/21 |
| A/Environment/Jiangsu/06691/2016-12-21 | Environment | Jiangsu | 2016/12/21 |
| A/Environment/Jiangsu/09386/2013-12-13 | Environment | Jiangsu | 2013/12/13 |
| A/Environment/Jiangsu/12052/2016-12-14 | Environment | Jiangsu | 2016/12/14 |
| A/Environment/Jiangsu/12058/2016-12-14 | Environment | Jiangsu | 2016/12/14 |
| A/Environment/Jiangsu/12087/2016-12-29 | Environment | Jiangsu | 2016/12/29 |
| A/Environment/Jiangsu/12089/2016-12-29 | Environment | Jiangsu | 2016/12/29 |
| A/Environment/Jiangsu/12090/2016-12-29 | Environment | Jiangsu | 2016/12/29 |
| A/Environment/Jiangsu/12096/2016-12-29 | Environment | Jiangsu | 2016/12/29 |
| A/Environment/Jiangsu/12105/2017-02-03 | Environment | Jiangsu | 2017/2/3 |
| A/Environment/Jiangsu/98337/2014-10-28 | Environment | Jiangsu | 2014/10/28 |
| A/environment/Jiangxi/02.05_ShRXZ038/2017-02-05 | environment | Jiangxi | 2017/2/5 |
| A/environment/Nanjing/2913/2013-03-29 | environment | Nanjing | 2013/3/29 |
| A/environment/Qinghai/S3032/2014-10-28 | environment | Qinghai | 2014/10/28 |
| A/Environment/SDDY/07/2015-03-08 | Environment | SDDY | 2015/3/8 |
| A/Environment/SDTA/04/2015-03-08 | Environment | SDTA | 2015/3/8 |
| A/Environment/Shaanxi/29413/2017-05-30 | Environment | Shaanxi | 2017/5/30 |
| A/Environment/Shaanxi/29414/2017-05-05 | Environment | Shaanxi | 2017/5/5 |
| A/Environment/Shaanxi/29415/2017-05-05 | Environment | Shaanxi | 2017/5/5 |
| A/Environment/Shandong/1/2013-04-27 | Environment | Shandong | 2013/4/27 |
| A/environment/Shandong/SD038/2013-05-03 | environment | Shandong | 2013/5/3 |
| A/environment/Shandong/SD039/2013-05-03 | environment | Shandong | 2013/5/3 |
| A/environment/Shandong/SD049/2013-05-03 | environment | Shandong | 2013/5/3 |
| A/Environment/Shandong/YT001/2014-05-21 | Environment | Shandong | 2014/5/21 |
| A/Environment/Shandong/YT002/2014-05-21 | Environment | Shandong | 2014/5/21 |
| A/Environment/Shandong/YT003/2014-05-21 | Environment | Shandong | 2014/5/21 |
| A/Environment/Shandong/YT004/2014-05-21 | Environment | Shandong | 2014/5/21 |
| A/Environment/Shandong/YT005/2014-05-21 | Environment | Shandong | 2014/5/21 |
| A/Environment/Shandong/YT007/2014-05-21 | Environment | Shandong | 2014/5/21 |
| A/Environment/Shandong/YT009/2014-05-21 | Environment | Shandong | 2014/5/21 |
| A/Environment/Shandong/ZZ001/2014-02-17 | Environment | Shandong | 2014/2/17 |
| A/Environment/Shandong-Linyi/EV01/2015-01-16 | Environment | Shandong-Linyi | 2015/1/16 |
| A/Environment/Shandong-Qingdao/12/2017-01-18 | Environment | Shandong-Qingdao | 2017/1/18 |
| A/Environment/Shandong-Qingdao/13/2017-01-18 | Environment | Shandong-Qingdao | 2017/1/18 |
| A/Environment/Shandong-Qingdao/14/2017-01-18 | Environment | Shandong-Qingdao | 2017/1/18 |
| A/Environment/Shandong-Qingdao/16/2017-01-18 | Environment | Shandong-Qingdao | 2017/1/18 |
| A/Environment/Shandong-Qingdao/18/2017-01-18 | Environment | Shandong-Qingdao | 2017/1/18 |
| A/Environment/Shandong-Qingdao/24/2017-01-24 | Environment | Shandong-Qingdao | 2017/1/24 |
| A/Environment/Shandong-Qingdao/25/2017-01-24 | Environment | Shandong-Qingdao | 2017/1/24 |
| A/Environment/Shandong-Qingdao/26/2017-01-24 | Environment | Shandong-Qingdao | 2017/1/24 |
| A/Environment/Shandong-Qingdao/28/2017-01-24 | Environment | Shandong-Qingdao | 2017/1/24 |
| A/Environment/Shandong-Rizhao/06/2017-01-03 | Environment | Shandong-Rizhao | 2017/1/3 |
| A/Environment/Shandong-Rizhao/08/2017-01-03 | Environment | Shandong-Rizhao | 2017/1/3 |
| A/Environment/Shandong-Taian/EV01/2015-03-09 | Environment | Shandong-Taian | 2015/3/9 |
| A/Environment/Shandong-Taian/EV02/2015-03-09 | Environment | Shandong-Taian | 2015/3/9 |
| A/Environment/Shandong-Taian/EV03/2015-03-09 | Environment | Shandong-Taian | 2015/3/9 |
| A/Environment/Shandong-Zaozhuang/32/2017-02-22 | Environment | Shandong-Zaozhuang | 2017/2/22 |
| A/Environment/Shandong-Zaozhuang/33/2017-02-22 | Environment | Shandong-Zaozhuang | 2017/2/22 |
| A/environment/Shanghai/PD-JZ-01/2014-01-21 | environment | Shanghai | 2014/1/21 |
| A/environment/Shanghai/RL01/2013-03-30 | environment | Shanghai | 2013/3/30 |
| A/Environment/Shanghai/S1088/2013-04-03 | Environment | Shanghai | 2013/4/3 |
| A/environment/Shanghai/S1436/2013-04-03 | environment | Shanghai | 2013/4/3 |
| A/environment/Shanghai/S1437/2013-04-03 | environment | Shanghai | 2013/4/3 |
| A/environment/Shanghai/S1438/2013-04-03 | environment | Shanghai | 2013/4/3 |
| A/environment/Shanghai/S1439/2013-04-03 | environment | Shanghai | 2013/4/3 |
| A/environment/Shanghai/TY01/2013/04/03 | environment | Shanghai | 2013/4/3 |
| A/environment/Suzhou/14/2013-04-04 | environment | Suzhou | 2013/4/4 |
| A/environment/Suzhou/8/2013-04-08 | environment | Suzhou | 2013/4/8 |
| A/environment/Wuxi/1/2013-04-02 | environment | Wuxi | 2013/4/2 |
| A/environment/Wuxi/Huan/2014-06-01 | environment | Wuxi | 2014/6/1 |
| A/Environment/Xinjiang/00344/2014-10-23 | Environment | Xinjiang | 2014/10/23 |
| A/Environment/Xinjiang/00347/2014-10-23 | Environment | Xinjiang | 2014/10/23 |
| A/Environment/Xinjiang/227303/2017-06-28 | Environment | Xinjiang | 2017/6/28 |
| A/Environment/Xinjiang/32311/2017-06-28 | Environment | Xinjiang | 2017/6/28 |
| A/Environment/Xinjiang/32314/2017-06-28 | Environment | Xinjiang | 2017/6/28 |
| A/Environment/Xinjiang/32316/2017-06-28 | Environment | Xinjiang | 2017/6/28 |
| A/Environment/Xinjiang/32319/2017-06-28 | Environment | Xinjiang | 2017/6/28 |
| A/Environment/Xinjiang/32320/2017-06-28 | Environment | Xinjiang | 2017/6/28 |
| A/Environment/Xinjiang/73033/2014-07-25 | Environment | Xinjiang | 2014/7/25 |
| A/Environment/Xinjiang/98679/2014-11-28 | Environment | Xinjiang | 2014/11/28 |
| A/Environment/Zhejiang/07813/2013-12-19 | Environment | Zhejiang | 2013/12/19 |
| A/Environment/Zhejiang/07819/2014-01-15 | Environment | Zhejiang | 2014/1/15 |
| A/Environment/Zhejiang/2/2014-02-25 | Environment | Zhejiang | 2014/2/25 |
| A/environment/Zhenjiang/4/2013-04-07 | environment | Zhenjiang | 2013/4/7 |
| A/Environmental/Guangdong/00307/2013-12-16 | Environmental | Guangdong | 2013/12/16 |
| A/Environmental/Guangdong/00329/2013-12-16 | Environmental | Guangdong | 2013/12/16 |
| A/Environment/SDYT/01/2016-02-17 | Environment | SDYT | 2016/2/17 |
| A/Environment/SDYT/02/2016-02-17 | Environment | SDYT | 2016/2/17 |
| A/Environment/SDYT/03/2016-02-17 | Environment | SDYT | 2016/2/17 |
| A/Environment/SDYT/04/2016-02-17 | Environment | SDYT | 2016/2/17 |
| A/Environment/SDYT/05/2016-02-17 | Environment | SDYT | 2016/2/17 |
| A/Environment/SDYT/06/2016-02-17 | Environment | SDYT | 2016/2/17 |
| A/Environment/SDYT/08/2016-02-17 | Environment | SDYT | 2016/2/17 |
| A/Environment/SDYT/10/2016-02-17 | Environment | SDYT | 2016/2/17 |
| A/Environmentironment/SDTA/11/2015-03-10 | Environmentironment | SDTA | 2015/3/10 |
| A/Environmentironment/SDTA/23/2015-03-10 | Environmentironment | SDTA | 2015/3/10 |
| A/Fujian/01/2013-04-23 | Human | Fujian | 2013/4/23 |
| A/Fujian/02/2013-04-27 | Human | Fujian | 2013/4/27 |
| A/Fujian/02150/2016-12-27 | Human | Fujian | 2016/12/27 |
| A/Fujian/02151/2017-01-01 | Human | Fujian | 2017/1/1 |
| A/Fujian/02152/2017-01-06 | Human | Fujian | 2017/1/6 |
| A/Fujian/03/2013-04-29 | Human | Fujian | 2013/4/29 |
| A/Fujian/04/2013-05-02 | Human | Fujian | 2013/5/2 |
| A/Fujian/05/2013-04-27 | Human | Fujian | 2013/4/27 |
| A/Fujian/09273/2015-01-12 | Human | Fujian | 2015/1/12 |
| A/Fujian/1/2013-04-24 | Human | Fujian | 2013/4/24 |
| A/Fujian/1/2014-01-14 | Human | Fujian | 2014/1/14 |
| A/Fujian/1/2015-01-01 | Human | Fujian | 2015/1/1 |
| A/Fujian/1/2016_\|_\|_2016-01-13 | Human | Fujian | 2016/1/13 |
| A/Fujian/10/2015-01-14 | Human | Fujian | 2015/1/14 |
| A/Fujian/10953/2017-02-05 | Human | Fujian | 2017/2/5 |
| A/Fujian/11/2014-01-26 | Human | Fujian | 2014/1/26 |
| A/Fujian/11/2015-01-15 | Human | Fujian | 2015/1/15 |
| A/Fujian/11/2017-02-03 | Human | Fujian | 2017/2/3 |
| A/Fujian/12/2014-01-31 | Human | Fujian | 2014/1/31 |
| A/Fujian/12/2017-02-04 | Human | Fujian | 2017/2/4 |
| A/Fujian/13/2014-02-02 | Human | Fujian | 2014/2/2 |
| A/Fujian/13558/2017-02-16 | Human | Fujian | 2017/2/16 |
| A/Fujian/14/2014-02-05 | Human | Fujian | 2014/2/5 |
| A/Fujian/14/2015-01-11 | Human | Fujian | 2015/1/11 |
| A/Fujian/14/2017-01-24 | Human | Fujian | 2017/1/24 |
| A/Fujian/15/2014-02-26 | Human | Fujian | 2014/2/26 |
| A/Fujian/16/2014-03-28 | Human | Fujian | 2014/3/28 |
| A/Fujian/16/2015-01-15 | Human | Fujian | 2015/1/15 |
| A/Fujian/17/2014-12-02 | Human | Fujian | 2014/12/2 |
| A/Fujian/17/2015-01-15 | Human | Fujian | 2015/1/15 |
| A/Fujian/17/2017-02-16 | Human | Fujian | 2017/2/16 |
| A/Fujian/17527/2016-02-26 | Human | Fujian | 2016/2/26 |
| A/Fujian/18/2015_\|_\|_2015-01-15 | Human | Fujian | 2015/1/15 |
| A/Fujian/19/2014-12-21 | Human | Fujian | 2014/12/21 |
| A/Fujian/19/2015-01-16 | Human | Fujian | 2015/1/16 |
| A/Fujian/2/2014-01-15 | Human | Fujian | 2014/1/15 |
| A/Fujian/2/2015-01-03 | Human | Fujian | 2015/1/3 |
| A/Fujian/20/2014-12-23 | Human | Fujian | 2014/12/23 |
| A/Fujian/20/2015-01-16 | Human | Fujian | 2015/1/16 |
| A/Fujian/21/2014-12-29 | Human | Fujian | 2014/12/29 |
| A/Fujian/21/2015-01-17 | Human | Fujian | 2015/1/17 |
| A/Fujian/22/2014-12-29 | Human | Fujian | 2014/12/29 |
| A/Fujian/22/2015-01-17 | Human | Fujian | 2015/1/17 |
| A/Fujian/23/2015-01-19 | Human | Fujian | 2015/1/19 |
| A/Fujian/24/2015-01-18 | Human | Fujian | 2015/1/18 |
| A/Fujian/25/2015-01-22 | Human | Fujian | 2015/1/22 |
| A/Fujian/27/2015-02-02 | Human | Fujian | 2015/2/2 |
| A/Fujian/27871/2017-05-11 | Human | Fujian | 2017/5/11 |
| A/Fujian/28/2015-03-15 | Human | Fujian | 2015/3/15 |
| A/Fujian/3/2014-01-16 | Human | Fujian | 2014/1/16 |
| A/Fujian/3/2015-01-04 | Human | Fujian | 2015/1/4 |
| A/Fujian/3/2016-01-15 | Human | Fujian | 2016/1/15 |
| A/Fujian/30766/2016-03-15 | Human | Fujian | 2016/3/15 |
| A/Fujian/30767/2016-02-22 | Human | Fujian | 2016/2/22 |
| A/Fujian/4/2015-01-05 | Human | Fujian | 2015/1/5 |
| A/Fujian/45970/2016-06-30 | Human | Fujian | 2016/6/30 |
| A/Fujian/5/2014-01-24 | Human | Fujian | 2014/1/24 |
| A/Fujian/5/2015-01-07 | Human | Fujian | 2015/1/7 |
| A/Fujian/54840/2016-11-16 | Human | Fujian | 2016/11/16 |
| A/Fujian/56600/2016-11-20 | Human | Fujian | 2016/11/20 |
| A/Fujian/6/2014-01-26 | Human | Fujian | 2014/1/26 |
| A/Fujian/6/2015-01-10 | Human | Fujian | 2015/1/10 |
| A/Fujian/6/2017-01-21 | Human | Fujian | 2017/1/21 |
| A/Fujian/7/2014-01-27 | Human | Fujian | 2014/1/27 |
| A/Fujian/7/2015-01-12 | Human | Fujian | 2015/1/12 |
| A/Fujian/8/2014-02-03 | Human | Fujian | 2014/2/3 |
| A/Fujian/8/2015-01-12 | Human | Fujian | 2015/1/12 |
| A/Fujian/8/2017-01-23 | Human | Fujian | 2017/1/23 |
| A/Fujian/9/2014-01-13 | Human | Fujian | 2014/1/13 |
| A/Fujian/9/2015-01-13 | Human | Fujian | 2015/1/13 |
| A/Fujian/QZ-Th001/2017-05-15 | Human | Fujian | 2017/5/15 |
| A/Fujian/QZ-Th002/2017-05-15 | Human | Fujian | 2017/5/15 |
| A/Fujian/QZ-Th005/2017-05-15 | Human | Fujian | 2017/5/15 |
| A/Fujian/S03/2015-01-23 | Human | Fujian | 2015/1/23 |
| A/Gansu/19545/2017-04-03 | Human | Gansu | 2017/4/3 |
| A/Gansu/27151/2017-04-21 | Human | Gansu | 2017/4/21 |
| A/goose/Jiangsu/1027/2013-04-06 | goose | Jiangsu | 2013/4/6 |
| A/Guangdong/0010/2014-01-03 | Human | Guangdong | 2014/1/3 |
| A/Guangdong/0012/2014-01-04 | Human | Guangdong | 2014/1/4 |
| A/Guangdong/0019/2014-01-08 | Human | Guangdong | 2014/1/8 |
| A/Guangdong/0024/2014-01-10 | Human | Guangdong | 2014/1/10 |
| A/Guangdong/0026/2014-01-11 | Human | Guangdong | 2014/1/11 |
| A/Guangdong/0029/2014-01-09 | Human | Guangdong | 2014/1/9 |
| A/Guangdong/0031/2014-01-12 | Human | Guangdong | 2014/1/12 |
| A/Guangdong/0046/2014-01-17 | Human | Guangdong | 2014/1/17 |
| A/Guangdong/0048/2014-01-20 | Human | Guangdong | 2014/1/20 |
| A/Guangdong/0065/2014-01-29 | Human | Guangdong | 2014/1/29 |
| A/Guangdong/0066/2014-01-29 | Human | Guangdong | 2014/1/29 |
| A/Guangdong/0069/2014-01-30 | Human | Guangdong | 2014/1/30 |
| A/Guangdong/0071/2014-01-30 | Human | Guangdong | 2014/1/30 |
| A/Guangdong/0074/2014-01-30 | Human | Guangdong | 2014/1/30 |
| A/Guangdong/0075/2014-01-30 | Human | Guangdong | 2014/1/30 |
| A/Guangdong/0081/2014-01-31 | Human | Guangdong | 2014/1/31 |
| A/Guangdong/02124/2014-01-04 | Human | Guangdong | 2014/1/4 |
| A/Guangdong/02125/2014-01-08 | Human | Guangdong | 2014/1/8 |
| A/Guangdong/02496/2014-11-27 | Human | Guangdong | 2014/11/27 |
| A/Guangdong/02497/2014-12-03 | Human | Guangdong | 2014/12/3 |
| A/Guangdong/02620/2014-01-08 | Human | Guangdong | 2014/1/8 |
| A/Guangdong/03/2013-12-15 | Human | Guangdong | 2013/12/15 |
| A/Guangdong/033/2014-01-13 | Human | Guangdong | 2014/1/13 |
| A/Guangdong/034/2014-01-13 | Human | Guangdong | 2014/1/13 |
| A/Guangdong/035/2014-01-13 | Human | Guangdong | 2014/1/13 |
| A/Guangdong/036/2014-01-13 | Human | Guangdong | 2014/1/13 |
| A/Guangdong/037/2014-01-14 | Human | Guangdong | 2014/1/14 |
| A/Guangdong/04/2013-12-16 | Human | Guangdong | 2013/12/16 |
| A/Guangdong/05/2013-12-17 | Human | Guangdong | 2013/12/17 |
| A/Guangdong/1/2013-08-10 | Human | Guangdong | 2013/8/10 |
| A/Guangdong/15SF001/2015-01-04 | Human | Guangdong | 2015/1/4 |
| A/Guangdong/15SF002/2015-01-05 | Human | Guangdong | 2015/1/5 |
| A/Guangdong/15SF010/2015-01-06 | Human | Guangdong | 2015/1/6 |
| A/Guangdong/15SF017/2015-01-15 | Human | Guangdong | 2015/1/15 |
| A/Guangdong/15SF018/2015-01-12 | Human | Guangdong | 2015/1/12 |
| A/Guangdong/15SF020/2015-01-13 | Human | Guangdong | 2015/1/13 |
| A/Guangdong/15SF021/2015-01-17 | Human | Guangdong | 2015/1/17 |
| A/Guangdong/15SF027/2015-01-21 | Human | Guangdong | 2015/1/21 |
| A/Guangdong/15SF030/2015-01-22 | Human | Guangdong | 2015/1/22 |
| A/Guangdong/15SF043/2015-01-23 | Human | Guangdong | 2015/1/23 |
| A/Guangdong/15SF050/2015-01-28 | Human | Guangdong | 2015/1/28 |
| A/Guangdong/15SF051/2015-01-31 | Human | Guangdong | 2015/1/31 |
| A/Guangdong/15SF052/2015-01-21 | Human | Guangdong | 2015/1/21 |
| A/Guangdong/15SF053/2015-02-04 | Human | Guangdong | 2015/2/4 |
| A/Guangdong/15SF054/2015-01-22 | Human | Guangdong | 2015/1/22 |
| A/Guangdong/15SF055/2015-01-25 | Human | Guangdong | 2015/1/25 |
| A/Guangdong/15SF057/2015-02-04 | Human | Guangdong | 2015/2/4 |
| A/Guangdong/15SF080/2015-02-10 | Human | Guangdong | 2015/2/10 |
| A/Guangdong/15SF081/2015-02-10 | Human | Guangdong | 2015/2/10 |
| A/Guangdong/15SF082/2015-02-06 | Human | Guangdong | 2015/2/6 |
| A/Guangdong/16SF007/2016-01-12 | Human | Guangdong | 2016/1/12 |
| A/Guangdong/16SF202/2016-12-30 | Human | Guangdong | 2016/12/30 |
| A/Guangdong/16SF203/2016-12-30 | Human | Guangdong | 2016/12/30 |
| A/Guangdong/17SF001/2016-12-28 | Human | Guangdong | 2016/12/28 |
| A/Guangdong/17SF003/2016_____NIBRG-375\|_\|_2017-06-19 | Human | Guangdong | 2017/6/19 |
| A/Guangdong/17SF003/2017-01-03 | Human | Guangdong | 2017/1/3 |
| A/Guangdong/17SF003/2017-06-19 | Human | Guangdong | 2017/6/19 |
| A/Guangdong/17SF004/2017-01-06 | Human | Guangdong | 2017/1/6 |
| A/Guangdong/17SF006/2017-01-12 | Human | Guangdong | 2017/1/12 |
| A/Guangdong/17SF009/2017-01-16 | Human | Guangdong | 2017/1/16 |
| A/Guangdong/17SF010/2017-01-12 | Human | Guangdong | 2017/1/12 |
| A/Guangdong/17SF011/2017-01-22 | Human | Guangdong | 2017/1/22 |
| A/Guangdong/17SF017/2017-01-23 | Human | Guangdong | 2017/1/23 |
| A/Guangdong/17SF027/2017-02-07 | Human | Guangdong | 2017/2/7 |
| A/Guangdong/17SF028/2016-12-30 | Human | Guangdong | 2016/12/30 |
| A/Guangdong/17SF030/2017-02-07 | Human | Guangdong | 2017/2/7 |
| A/Guangdong/17SF031/2017-02-09 | Human | Guangdong | 2017/2/9 |
| A/Guangdong/17SF032/2017-01-23 | Human | Guangdong | 2017/1/23 |
| A/Guangdong/17SF033/2017-02-22 | Human | Guangdong | 2017/2/22 |
| A/Guangdong/17SF036/2017-02-25 | Human | Guangdong | 2017/2/25 |
| A/Guangdong/17SF037/2017-02-09 | Human | Guangdong | 2017/2/9 |
| A/Guangdong/17SF039/2017-02-15 | Human | Guangdong | 2017/2/15 |
| A/Guangdong/17SF042/2017-03-01 | Human | Guangdong | 2017/3/1 |
| A/Guangdong/17SF059/2017-03-06 | Human | Guangdong | 2017/3/6 |
| A/Guangdong/17SF060/2017-01-13 | Human | Guangdong | 2017/1/13 |
| A/Guangdong/17SF061/2017-01-19 | Human | Guangdong | 2017/1/19 |
| A/Guangdong/17SF062/2017-03-11 | Human | Guangdong | 2017/3/11 |
| A/Guangdong/17SF064/2017-03-12 | Human | Guangdong | 2017/3/12 |
| A/Guangdong/24997/2013-11-30 | Human | Guangdong | 2013/11/30 |
| A/Guangdong/24999/2013-12-16 | Human | Guangdong | 2013/12/16 |
| A/Guangdong/6/2013/10 | Human | Guangdong | Oct-13 |
| A/Guangdong/60060/2016-12-09 | Human | Guangdong | 2016/12/9 |
| A/Guangdong/60061/2016-12-13 | Human | Guangdong | 2016/12/13 |
| A/Guangdong/60923/2016-12-28 | Human | Guangdong | 2016/12/28 |
| A/Guangdong/7/2013/12 | Human | Guangdong | Dec-13 |
| A/Guangdong/8H324/2017-01-04 | Human | Guangdong | 2017/1/4 |
| A/Guangdong/DG-02/2013-11 | Human | Guangdong | Nov-13 |
| A/Guangdong/DG-03/2013-12 | Human | Guangdong | Dec-13 |
| A/Guangdong/HP001/2017-01-04 | Human | Guangdong | 2017/1/4 |
| A/Guangdong/MZ828/2015-11-21 | Human | Guangdong | 2015/11/21 |
| A/Guangdong/SP440/2017-01-04 | Human | Guangdong | 2017/1/4 |
| A/Guangdong/Th005/2017-02-01 | Human | Guangdong | 2017/2/1 |
| A/Guangdong/Th008/2017-01-04 | Human | Guangdong | 2017/1/4 |
| A/Guangdong/YJ-04/2013-12 | Human | Guangdong | Dec-13 |
| A/Guangdong/YJ-05/2013-12 | Human | Guangdong | Dec-13 |
| A/Guangdong/XN00175/2014-01-29 | Human | Guangdong | 2014/1/29 |
| A/Guangdong/XN00298/2014-02-02 | Human | Guangdong | 2014/2/2 |
| A/Guangdong/XN00392/2014-02-05 | Human | Guangdong | 2014/2/5 |
| A/Guangdong/XN00429/2014-02-05 | Human | Guangdong | 2014/2/5 |
| A/Guangdong/XN00457/2014-02-07 | Human | Guangdong | 2014/2/7 |
| A/Guangdong/XN00509/2014-02-10 | Human | Guangdong | 2014/2/10 |
| A/Guangdong/XN00588/2014-02-16 | Human | Guangdong | 2014/2/16 |
| A/Guangdong/XN02778/2014-01-15 | Human | Guangdong | 2014/1/15 |
| A/Guangdong/XN02933/2014-01-16 | Human | Guangdong | 2014/1/16 |
| A/Guangdong/XN05586/2014-01-29 | Human | Guangdong | 2014/1/29 |
| A/Guangdong/XN06587/2014-02-10 | Human | Guangdong | 2014/2/10 |
| A/Guangdong/XN06839/2014-02-11 | Human | Guangdong | 2014/2/11 |
| A/Guangdong/XN08423/2014-02-19 | Human | Guangdong | 2014/2/19 |
| A/Guangdong/XN08664/2014-02-21 | Human | Guangdong | 2014/2/21 |
| A/Guangdong/XN08747/2014-02-21 | Human | Guangdong | 2014/2/21 |
| A/Guangdong/XN08763/2014-02-21 | Human | Guangdong | 2014/2/21 |
| A/Guangdong/XN09086/2014-02-24 | Human | Guangdong | 2014/2/24 |
| A/Guangdong/XN13392/2014-03-24 | Human | Guangdong | 2014/3/24 |
| A/Guangdong/XN14050/2014-03-31 | Human | Guangdong | 2014/3/31 |
| A/Guangdong/XN14670/2014-04-04 | Human | Guangdong | 2014/4/4 |
| A/Guangdong/XN15122/2014-04-10 | Human | Guangdong | 2014/4/10 |
| A/Guangxi/08309/2014-01-27 | Human | Guangxi | 2014/1/27 |
| A/Guangxi/08970/2014-02-03 | Human | Guangxi | 2014/2/3 |
| A/Guangxi/08971/2014-02-05 | Human | Guangxi | 2014/2/5 |
| A/Guangxi/1/2017-02-18 | Human | Guangxi | 2017/2/18 |
| A/Guangxi/13450/2017-02-23 | Human | Guangxi | 2017/2/23 |
| A/Guangxi/13452/2017-02-18 | Human | Guangxi | 2017/2/18 |
| A/Guangxi/18888/2017-02-28 | Human | Guangxi | 2017/2/28 |
| A/Guangxi/18891/2017-02-27 | Human | Guangxi | 2017/2/27 |
| A/Guangxi/18892/2017-02-27 | Human | Guangxi | 2017/2/27 |
| A/Guangxi/18895/2017-03-03 | Human | Guangxi | 2017/3/3 |
| A/Guangxi/18897/2017-03-04 | Human | Guangxi | 2017/3/4 |
| A/Guangxi/18899/2017-03-06 | Human | Guangxi | 2017/3/6 |
| A/Guangxi/18902/2017-03-12 | Human | Guangxi | 2017/3/12 |
| A/Guangxi/18903/2017-03-12 | Human | Guangxi | 2017/3/12 |
| A/Guangxi/18905/2017-03-12 | Human | Guangxi | 2017/3/12 |
| A/Guangxi/18906/2017-03-14 | Human | Guangxi | 2017/3/14 |
| A/Guangxi/18907/2017-03-14 | Human | Guangxi | 2017/3/14 |
| A/Guangxi/18908/2017-03-16 | Human | Guangxi | 2017/3/16 |
| A/Guangxi/18910/2017-03-18 | Human | Guangxi | 2017/3/18 |
| A/Guangxi/18911/2017-03-18 | Human | Guangxi | 2017/3/18 |
| A/Guangxi/2/2017-02-03 | Human | Guangxi | 2017/2/3 |
| A/Guangxi/3/2017-02-22 | Human | Guangxi | 2017/2/22 |
| A/Guangxi/4/2017-02-27 | Human | Guangxi | 2017/2/27 |
| A/Guangxi/5/2017-02-28 | Human | Guangxi | 2017/2/28 |
| A/Guangxi/6/2017-03-03 | Human | Guangxi | 2017/3/3 |
| A/Guangxi/7/2017-03-04 | Human | Guangxi | 2017/3/4 |
| A/Guangzhou/1/2014-01-26 | Human | Guangzhou | 2014/1/26 |
| A/Guangzhou/2/2014-01-26 | Human | Guangzhou | 2014/1/26 |
| A/Guizhou/01502/2014-01-08 | Human | Guizhou | 2014/1/8 |
| A/Guizhou/03240/2015-01-09 | Human | Guizhou | 2015/1/9 |
| A/Guizhou/07733/2017-01-10 | Human | Guizhou | 2017/1/10 |
| A/Guizhou/07734/2017-01-07 | Human | Guizhou | 2017/1/7 |
| A/Guizhou/18973/2017-03-07 | Human | Guizhou | 2017/3/7 |
| A/Guizhou/18975/2017-03-25 | Human | Guizhou | 2017/3/25 |
| A/Guizhou/18976/2017-03-25 | Human | Guizhou | 2017/3/25 |
| A/Guizhou/18977/2017-03-21 | Human | Guizhou | 2017/3/21 |
| A/Guizhou/18978/2017-03-10 | Human | Guizhou | 2017/3/10 |
| A/Guizhou/18980/2017-02-22 | Human | Guizhou | 2017/2/22 |
| A/Guizhou/21078/2017-03-01 | Human | Guizhou | 2017/3/1 |
| A/Hangzhou/1/2013-03-24 | Human | Hangzhou | 2013/3/24 |
| A/Hangzhou/10-1/2014-01-13 | Human | Hangzhou | 2014/1/13 |
| A/Hangzhou/112/2014-02-09 | Human | Hangzhou | 2014/2/9 |
| A/Hangzhou/118/2014-02-10 | Human | Hangzhou | 2014/2/10 |
| A/Hangzhou/169/2013-04-11 | Human | Hangzhou | 2013/4/11 |
| A/Hangzhou/17-1/2014-01-18 | Human | Hangzhou | 2014/1/18 |
| A/Hangzhou/173/2013-04-11 | Human | Hangzhou | 2013/4/11 |
| A/Hangzhou/19/2014-01-18 | Human | Hangzhou | 2014/1/18 |
| A/Hangzhou/2/2013-03-25 | Human | Hangzhou | 2013/3/25 |
| A/Hangzhou/254/2013-04-17 | Human | Hangzhou | 2013/4/17 |
| A/Hangzhou/3/2013-04-02 | Human | Hangzhou | 2013/4/2 |
| A/Hangzhou/30/2014-01-20 | Human | Hangzhou | 2014/1/20 |
| A/Hangzhou/327/2013-04-19 | Human | Hangzhou | 2013/4/19 |
| A/Hangzhou/35/2014-01-23 | Human | Hangzhou | 2014/1/23 |
| A/Hangzhou/38/2014-01-23 | Human | Hangzhou | 2014/1/23 |
| A/Hangzhou/39/2014-01-23 | Human | Hangzhou | 2014/1/23 |
| A/Hangzhou/40/2014-01-24 | Human | Hangzhou | 2014/1/24 |
| A/Hangzhou/49/2014-01-29 | Human | Hangzhou | 2014/1/29 |
| A/Hebei/01/2013-07-19 | Human | Hebei | 2013/7/19 |
| A/Hebei/22042/2017-04-15 | Human | Hebei | 2017/4/15 |
| A/Hebei/22044/2017-04-14 | Human | Hebei | 2017/4/14 |
| A/Hebei/22045/2017-04-13 | Human | Hebei | 2017/4/13 |
| A/Hebei/24991/2017-04-22 | Human | Hebei | 2017/4/22 |
| A/Hebei/24992/2017-04-27 | Human | Hebei | 2017/4/27 |
| A/Hebei/24994/2017-04-29 | Human | Hebei | 2017/4/29 |
| A/Hebei/24995/2017-04-30 | Human | Hebei | 2017/4/30 |
| A/Hebei/24997/2017-04-29 | Human | Hebei | 2017/4/29 |
| A/Hebei/27400/2017-05-05 | Human | Hebei | 2017/5/5 |
| A/Hebei/27401/2017-05-04 | Human | Hebei | 2017/5/4 |
| A/Hebei/27402/2017-05-06 | Human | Hebei | 2017/5/6 |
| A/Hebei/27403/2017-05-07 | Human | Hebei | 2017/5/7 |
| A/Hebei/27404/2017-05-08 | Human | Hebei | 2017/5/8 |
| A/Hebei/27407/2017-05-13 | Human | Hebei | 2017/5/13 |
| A/Hebei/27408/2017-05-15 | Human | Hebei | 2017/5/15 |
| A/Hebei/28939/2017-05-24 | Human | Hebei | 2017/5/24 |
| A/Hebei/28940/2017-05-27 | Human | Hebei | 2017/5/27 |
| A/Hebei/38604/2016-05-16 | Human | Hebei | 2016/5/16 |
| A/Hebei/46586/2016-07-27 | Human | Hebei | 2016/7/27 |
| A/Hebei/46587/2016-07-28 | Human | Hebei | 2016/7/28 |
| A/Henan/01/2013-04-16 | Human | Henan | 2013/4/16 |
| A/Henan/02/2013-04-23 | Human | Henan | 2013/4/23 |
| A/Henan/11156/2017-01-21 | Human | Henan | 2017/1/21 |
| A/Henan/11157/2017-02-04 | Human | Henan | 2017/2/4 |
| A/Henan/11158/2017-02-07 | Human | Henan | 2017/2/7 |
| A/Henan/11159/2017-02-08 | Human | Henan | 2017/2/8 |
| A/Henan/13705/2017-02-21 | Human | Henan | 2017/2/21 |
| A/Henan/13706/2017-02-21 | Human | Henan | 2017/2/21 |
| A/Henan/14903/2017-03-01 | Human | Henan | 2017/3/1 |
| A/Henan/14905/2017-03-04 | Human | Henan | 2017/3/4 |
| A/Henan/15336/2017-03-07 | Human | Henan | 2017/3/7 |
| A/Henan/15337/2017-03-04 | Human | Henan | 2017/3/4 |
| A/Henan/15338/2017-03-13 | Human | Henan | 2017/3/13 |
| A/Henan/15339/2017-03-10 | Human | Henan | 2017/3/10 |
| A/Henan/17867/2017-03-18 | Human | Henan | 2017/3/18 |
| A/Henan/21080/2017-04-08 | Human | Henan | 2017/4/8 |
| A/Henan/22190/2017-04-11 | Human | Henan | 2017/4/11 |
| A/Henan/27454/2017-05-02 | Human | Henan | 2017/5/2 |
| A/Henan/27456/2017-05-10 | Human | Henan | 2017/5/10 |
| A/Henan/29263/2017-06-05 | Human | Henan | 2017/6/5 |
| A/Henan/32240/2017-06-06 | Human | Henan | 2017/6/6 |
| A/homing_pigeon/Jiangsu/SD184/2013-04-20 | homing_pigeon | Jiangsu | 2013/4/20 |
| A/Hong_Kong/125/2017-01-05 | Human | Hong_Kong | 2017/1/5 |
| A/Hong_Kong/214/2017-01-09 | Human | Hong_Kong | 2017/1/9 |
| A/Hong_Kong/2212982/2014-01-28 | Human | Hong_Kong | 2014/1/28 |
| A/Hong_Kong/2550/2015-01-23 | Human | Hong_Kong | 2015/1/23 |
| A/Hong_Kong/3263/2014-02-12 | Human | Hong_Kong | 2014/2/12 |
| A/Hong_Kong/4495/2014-03-01 | Human | Hong_Kong | 2014/3/1 |
| A/Hong_Kong/470129/2013-11-30 | Human | Hong_Kong | 2013/11/30 |
| A/Hong_Kong/5581/2014-04-03 | Human | Hong_Kong | 2014/4/3 |
| A/Hong_Kong/5731/2014-04-08 | Human | Hong_Kong | 2014/4/8 |
| A/Hong_Kong/5942/2013-11-30 | Human | Hong_Kong | 2013/11/30 |
| A/Hong_Kong/734/2014-01-07 | Human | Hong_Kong | 2014/1/7 |
| A/Hong_Kong/8113530/2014-03-17 | Human | Hong_Kong | 2014/3/17 |
| A/Hong_Kong/8122430/2014-04-13 | Human | Hong_Kong | 2014/4/13 |
| A/Hong_Kong/8130773/2015-02-22 | Human | Hong_Kong | 2015/2/22 |
| A/Hong_Kong/8194273/2014-12-25 | Human | Hong_Kong | 2014/12/25 |
| A/Hong_Kong/VB16021618/2016-02-12 | Human | Hong_Kong | 2016/2/12 |
| A/Hong_Kong/VB16049808/2016-03-24 | Human | Hong_Kong | 2016/3/24 |
| A/Hong_Kong/VB16064646/2016-04-19 | Human | Hong_Kong | 2016/4/19 |
| A/Hong_Kong/VB16184091/2016-12-19 | Human | Hong_Kong | 2016/12/19 |
| A/Hong_Kong/VB16189623/2016-12-28 | Human | Hong_Kong | 2016/12/28 |
| A/Hong_Kong/VB17002884/2017-01-05 | Human | Hong_Kong | 2017/1/5 |
| A/Hong_Kong/VB17037915/2017-03-06 | Human | Hong_Kong | 2017/3/6 |
| A/Huaian/001/2015-01-23 | Human | Huaian | 2015/1/23 |
| A/Huaian/002/2015-02-03 | Human | Huaian | 2015/2/3 |
| A/Huaian/062/2014-02-14 | Human | Huaian | 2014/2/14 |
| A/Huaian/065/2014-02-24 | Human | Huaian | 2014/2/24 |
| A/Huaian/074/2014-03-02 | Human | Huaian | 2014/3/2 |
| A/Huaian/083/2014-05-27 | Human | Huaian | 2014/5/27 |
| A/Hubei/09907/2017-01-31 | Human | Hubei | 2017/1/31 |
| A/Hubei/09909/2017-02-07 | Human | Hubei | 2017/2/7 |
| A/Hubei/09910/2017-02-03 | Human | Hubei | 2017/2/3 |
| A/Hubei/09911/2017-02-04 | Human | Hubei | 2017/2/4 |
| A/Hubei/09912/2017-02-04 | Human | Hubei | 2017/2/4 |
| A/Hubei/09913/2017-02-06 | Human | Hubei | 2017/2/6 |
| A/Hubei/09914/2017-02-06 | Human | Hubei | 2017/2/6 |
| A/Hubei/09929/2017-01-26 | Human | Hubei | 2017/1/26 |
| A/Hubei/09937/2017-02-04 | Human | Hubei | 2017/2/4 |
| A/Hubei/11944/2017-02-20 | Human | Hubei | 2017/2/20 |
| A/Hubei/11950/2017-02-20 | Human | Hubei | 2017/2/20 |
| A/Hubei/11951/2017-02-20 | Human | Hubei | 2017/2/20 |
| A/Hubei/34007/2015-04-22 | Human | Hubei | 2015/4/22 |
| A/Hubei/39269/2016-03-14 | Human | Hubei | 2016/3/14 |
| A/Huizhou/01/2013-08-08 | Human | Huizhou | 2013/8/8 |
| A/Hunan/00001/2016-12-27 | Human | Hunan | 2016/12/27 |
| A/Hunan/0070/2013-04-29 | Human | Hunan | 2013/4/29 |
| A/Hunan/0070A/2013-04 | Human | Hunan | Apr-13 |
| A/Hunan/01/2013-04-24 | Human | Hunan | 2013/4/24 |
| A/Hunan/02/2013-04-25 | Human | Hunan | 2013/4/25 |
| A/Hunan/02285/2017-01-08 | Human | Hunan | 2017/1/8 |
| A/Hunan/02286/2017-01-06 | Human | Hunan | 2017/1/6 |
| A/Hunan/02287/2017-01-11 | Human | Hunan | 2017/1/11 |
| A/Hunan/02650/2016-01-26 | Human | Hunan | 2016/1/26 |
| A/Hunan/04135/2016-01-01 | Human | Hunan | 2016/1/1 |
| A/Hunan/04137/2016-01-02 | Human | Hunan | 2016/1/2 |
| A/Hunan/06196/2016-02-03 | Human | Hunan | 2016/2/3 |
| A/Hunan/06943/2017-01-13 | Human | Hunan | 2017/1/13 |
| A/Hunan/06944/2017-01-13 | Human | Hunan | 2017/1/13 |
| A/Hunan/06945/2017-01-15 | Human | Hunan | 2017/1/15 |
| A/Hunan/06946/2017-01-16 | Human | Hunan | 2017/1/16 |
| A/Hunan/06947/2017-01-09 | Human | Hunan | 2017/1/9 |
| A/Hunan/06948/2017-01-16 | Human | Hunan | 2017/1/16 |
| A/Hunan/06949/2017-01-18 | Human | Hunan | 2017/1/18 |
| A/Hunan/07833/2014-01-27 | Human | Hunan | 2014/1/27 |
| A/Hunan/08591/2017-01-22 | Human | Hunan | 2017/1/22 |
| A/Hunan/08593/2017-01-18 | Human | Hunan | 2017/1/18 |
| A/Hunan/08963/2014-02-02 | Human | Hunan | 2014/2/2 |
| A/Hunan/09191/2014-02-11 | Human | Hunan | 2014/2/11 |
| A/Hunan/09193/2014-02-05 | Human | Hunan | 2014/2/5 |
| A/Hunan/09917/2017-02-06 | Human | Hunan | 2017/2/6 |
| A/Hunan/09918/2017-02-04 | Human | Hunan | 2017/2/4 |
| A/Hunan/09920/2017-01-31 | Human | Hunan | 2017/1/31 |
| A/Hunan/09921/2017-01-25 | Human | Hunan | 2017/1/25 |
| A/Hunan/09922/2017-01-23 | Human | Hunan | 2017/1/23 |
| A/Hunan/11025/2017-02-10 | Human | Hunan | 2017/2/10 |
| A/Hunan/11026/2017-02-11 | Human | Hunan | 2017/2/11 |
| A/Hunan/12172/2014-02-16 | Human | Hunan | 2014/2/16 |
| A/Hunan/12173/2014-02-17 | Human | Hunan | 2014/2/17 |
| A/Hunan/17868/2017-03-17 | Human | Hunan | 2017/3/17 |
| A/Hunan/17869/2017-03-12 | Human | Hunan | 2017/3/12 |
| A/Hunan/17872/2017-03-21 | Human | Hunan | 2017/3/21 |
| A/Hunan/17873/2017-03-04 | Human | Hunan | 2017/3/4 |
| A/Hunan/17874/2017-03-06 | Human | Hunan | 2017/3/6 |
| A/Hunan/17875/2017-02-28 | Human | Hunan | 2017/2/28 |
| A/Hunan/19762/2015-02-10 | Human | Hunan | 2015/2/10 |
| A/Hunan/19763/2015-02-10 | Human | Hunan | 2015/2/10 |
| A/Hunan/25349/2017-04-13 | Human | Hunan | 2017/4/13 |
| A/Hunan/25350/2017-03-29 | Human | Hunan | 2017/3/29 |
| A/Hunan/25351/2017-04-11 | Human | Hunan | 2017/4/11 |
| A/Hunan/25353/2017-03-14 | Human | Hunan | 2017/3/14 |
| A/Hunan/25355/2017-03-29 | Human | Hunan | 2017/3/29 |
| A/Hunan/25356/2017-03-23 | Human | Hunan | 2017/3/23 |
| A/Hunan/25358/2017-03-24 | Human | Hunan | 2017/3/24 |
| A/Hunan/25360/2017-02-04 | Human | Hunan | 2017/2/4 |
| A/Hunan/25362/2017-03-14 | Human | Hunan | 2017/3/14 |
| A/Hunan/25363/2017-03-16 | Human | Hunan | 2017/3/16 |
| A/Hunan/25364/2017-03-19 | Human | Hunan | 2017/3/19 |
| A/Hunan/25365/2017-03-21 | Human | Hunan | 2017/3/21 |
| A/Hunan/26937/2014-04-19 | Human | Hunan | 2014/4/19 |
| A/Hunan/26938/2014-04-19 | Human | Hunan | 2014/4/19 |
| A/Hunan/27316/2017-02-28 | Human | Hunan | 2017/2/28 |
| A/Inner_Mongolia/29169/2017-06-14 | Human | Inner_Mongolia | 2017/6/14 |
| A/Inner_Mongolia/Tongliao29169/2017-06-14 | Human | Inner_Mongolia | 2017/6/14 |
| A/Jiangsu/01/2013-03-30 | Human | Jiangsu | 2013/3/30 |
| A/Jiangsu/02/2013-03-31 | Human | Jiangsu | 2013/3/31 |
| A/Jiangsu/03/2013-04-06 | Human | Jiangsu | 2013/4/6 |
| A/Jiangsu/03753/2016-01-20 | Human | Jiangsu | 2016/1/20 |
| A/Jiangsu/03756/2016-01-12 | Human | Jiangsu | 2016/1/12 |
| A/Jiangsu/04/2013-04-05 | Human | Jiangsu | 2013/4/5 |
| A/Jiangsu/05/2013-04-08 | Human | Jiangsu | 2013/4/8 |
| A/Jiangsu/06/2013-04-10 | Human | Jiangsu | 2013/4/10 |
| A/Jiangsu/06306/2014-12-27 | Human | Jiangsu | 2014/12/27 |
| A/Jiangsu/06307/2014-12-29 | Human | Jiangsu | 2014/12/29 |
| A/Jiangsu/06454/2017-01-06 | Human | Jiangsu | 2017/1/6 |
| A/Jiangsu/06455/2016-12-13 | Human | Jiangsu | 2016/12/13 |
| A/Jiangsu/06456/2017-01-09 | Human | Jiangsu | 2017/1/9 |
| A/Jiangsu/06462/2017-01-01 | Human | Jiangsu | 2017/1/1 |
| A/Jiangsu/06463/2017-01-07 | Human | Jiangsu | 2017/1/7 |
| A/Jiangsu/06464/2017-01-12 | Human | Jiangsu | 2017/1/12 |
| A/Jiangsu/07/2013-04-03 | Human | Jiangsu | 2013/4/3 |
| A/Jiangsu/08/2013-04-11 | Human | Jiangsu | 2013/4/11 |
| A/Jiangsu/08167/2016-12-16 | Human | Jiangsu | 2016/12/16 |
| A/Jiangsu/08173/2016-12-20 | Human | Jiangsu | 2016/12/20 |
| A/Jiangsu/08174/2016-12-19 | Human | Jiangsu | 2016/12/19 |
| A/Jiangsu/08178/2016-12-26 | Human | Jiangsu | 2016/12/26 |
| A/Jiangsu/08179/2016-12-27 | Human | Jiangsu | 2016/12/27 |
| A/Jiangsu/08180/2016-12-26 | Human | Jiangsu | 2016/12/26 |
| A/Jiangsu/08184/2016-12-28 | Human | Jiangsu | 2016/12/28 |
| A/Jiangsu/08186/2016-12-29 | Human | Jiangsu | 2016/12/29 |
| A/Jiangsu/08187/2016-12-30 | Human | Jiangsu | 2016/12/30 |
| A/Jiangsu/08188/2016-12-31 | Human | Jiangsu | 2016/12/31 |
| A/Jiangsu/08189/2016-12-31 | Human | Jiangsu | 2016/12/31 |
| A/Jiangsu/08190/2017-01-02 | Human | Jiangsu | 2017/1/2 |
| A/Jiangsu/08191/2017-01-03 | Human | Jiangsu | 2017/1/3 |
| A/Jiangsu/08192/2017-01-04 | Human | Jiangsu | 2017/1/4 |
| A/Jiangsu/08193/2017-01-04 | Human | Jiangsu | 2017/1/4 |
| A/Jiangsu/08195/2017-01-06 | Human | Jiangsu | 2017/1/6 |
| A/Jiangsu/08196/2017-01-06 | Human | Jiangsu | 2017/1/6 |
| A/Jiangsu/08197/2017-01-09 | Human | Jiangsu | 2017/1/9 |
| A/Jiangsu/08200/2016-12-24 | Human | Jiangsu | 2016/12/24 |
| A/Jiangsu/08201/2017-01-14 | Human | Jiangsu | 2017/1/14 |
| A/Jiangsu/08202/2016-12-19 | Human | Jiangsu | 2016/12/19 |
| A/Jiangsu/08203/2016-12-28 | Human | Jiangsu | 2016/12/28 |
| A/Jiangsu/08204/2016-12-23 | Human | Jiangsu | 2016/12/23 |
| A/Jiangsu/08206/2017-01-05 | Human | Jiangsu | 2017/1/5 |
| A/Jiangsu/08207/2016-12-30 | Human | Jiangsu | 2016/12/30 |
| A/Jiangsu/09/2013-04-09 | Human | Jiangsu | 2013/4/9 |
| A/Jiangsu/09041/2014-01-20 | Human | Jiangsu | 2014/1/20 |
| A/Jiangsu/09043/2014-01-26 | Human | Jiangsu | 2014/1/26 |
| A/Jiangsu/09049/2014-02-05 | Human | Jiangsu | 2014/2/5 |
| A/Jiangsu/09387/2014-02-07 | Human | Jiangsu | 2014/2/7 |
| A/Jiangsu/09389/2014-01-07 | Human | Jiangsu | 2014/1/7 |
| A/Jiangsu/1/2013-04-20 | Human | Jiangsu | 2013/4/20 |
| A/Jiangsu/11550/2017-01-23 | Human | Jiangsu | 2017/1/23 |
| A/Jiangsu/11553/2017-01-29 | Human | Jiangsu | 2017/1/29 |
| A/Jiangsu/11554/2017-02-03 | Human | Jiangsu | 2017/2/3 |
| A/Jiangsu/11555/2017-02-03 | Human | Jiangsu | 2017/2/3 |
| A/Jiangsu/11556/2017-02-01 | Human | Jiangsu | 2017/2/1 |
| A/Jiangsu/11557/2017-02-07 | Human | Jiangsu | 2017/2/7 |
| A/Jiangsu/11581/2017-01-13 | Human | Jiangsu | 2017/1/13 |
| A/Jiangsu/11582/2017-01-11 | Human | Jiangsu | 2017/1/11 |
| A/Jiangsu/11583/2017-01-29 | Human | Jiangsu | 2017/1/29 |
| A/Jiangsu/11585/2017-01-31 | Human | Jiangsu | 2017/1/31 |
| A/Jiangsu/11586/2017-02-03 | Human | Jiangsu | 2017/2/3 |
| A/Jiangsu/11587/2017-02-03 | Human | Jiangsu | 2017/2/3 |
| A/Jiangsu/11588/2017-02-03 | Human | Jiangsu | 2017/2/3 |
| A/Jiangsu/11589/2017-02-06 | Human | Jiangsu | 2017/2/6 |
| A/Jiangsu/11590/2017-02-07 | Human | Jiangsu | 2017/2/7 |
| A/Jiangsu/15026/2016-01-26 | Human | Jiangsu | 2016/1/26 |
| A/Jiangsu/18828/2014-12-31 | Human | Jiangsu | 2014/12/31 |
| A/Jiangsu/19757/2015-01-23 | Human | Jiangsu | 2015/1/23 |
| A/Jiangsu/19758/2015-01-23 | Human | Jiangsu | 2015/1/23 |
| A/Jiangsu/2/2013-04-20 | Human | Jiangsu | 2013/4/20 |
| A/Jiangsu/22184/2015-01-16 | Human | Jiangsu | 2015/1/16 |
| A/Jiangsu/24147/2017-02-26 | Human | Jiangsu | 2017/2/26 |
| A/Jiangsu/24149/2017-03-22 | Human | Jiangsu | 2017/3/22 |
| A/Jiangsu/24150/2017-01-31 | Human | Jiangsu | 2017/1/31 |
| A/Jiangsu/24154/2017-04-06 | Human | Jiangsu | 2017/4/6 |
| A/Jiangsu/24159/2017-01-18 | Human | Jiangsu | 2017/1/18 |
| A/Jiangsu/24161/2017-02-10 | Human | Jiangsu | 2017/2/10 |
| A/Jiangsu/33446/2015-02-01 | Human | Jiangsu | 2015/2/1 |
| A/Jiangsu/60447/2016-12-16 | Human | Jiangsu | 2016/12/16 |
| A/Jiangsu/60450/2016-12-19 | Human | Jiangsu | 2016/12/19 |
| A/Jiangsu/60451/2016-12-19 | Human | Jiangsu | 2016/12/19 |
| A/Jiangsu/60452/2016-12-19 | Human | Jiangsu | 2016/12/19 |
| A/Jiangsu/60454/2016-11-25 | Human | Jiangsu | 2016/11/25 |
| A/Jiangsu/60456/2016-12-02 | Human | Jiangsu | 2016/12/2 |
| A/Jiangsu/60457/2016-12-06 | Human | Jiangsu | 2016/12/6 |
| A/Jiangsu/60458/2016-12-10 | Human | Jiangsu | 2016/12/10 |
| A/Jiangsu/60459/2016-12-12 | Human | Jiangsu | 2016/12/12 |
| A/Jiangsu/60460/2016-12-13 | Human | Jiangsu | 2016/12/13 |
| A/Jiangsu/60461/2016-12-20 | Human | Jiangsu | 2016/12/20 |
| A/Jiangsu/60462/2016-12-16 | Human | Jiangsu | 2016/12/16 |
| A/Jiangsu/60463/2016-11-15 | Human | Jiangsu | 2016/11/15 |
| A/Jiangsu/60465/2016-12-09 | Human | Jiangsu | 2016/12/9 |
| A/Jiangsu/60466/2016-11-21 | Human | Jiangsu | 2016/11/21 |
| A/Jiangsu/60467/2016-10-26 | Human | Jiangsu | 2016/10/26 |
| A/Jiangsu/98342/2014-11-17 | Human | Jiangsu | 2014/11/17 |
| A/Jiangsu/FF16068/2016-12-16 | Human | Jiangsu | 2016/12/16 |
| A/Jiangsu/HM16001/2016-12-15 | Human | Jiangsu | 2016/12/15 |
| A/Jiangsu/JT530/2016-12-24 | Human | Jiangsu | 2016/12/24 |
| A/Jiangsu/Wuxi/Yangtze04/2013-04-26 | Human | Jiangsu | 2013/4/26 |
| A/Jiangsu/YY201602/2016-12-29 | Human | Jiangsu | 2016/12/29 |
| A/Jiangxi/01/2013-04-24 | Human | Jiangxi | 2013/4/24 |
| A/Jiangxi/10661/2017-01-03 | Human | Jiangxi | 2017/1/3 |
| A/Jiangxi/10662/2017-01-06 | Human | Jiangxi | 2017/1/6 |
| A/Jiangxi/10663/2017-01-05 | Human | Jiangxi | 2017/1/5 |
| A/Jiangxi/10665/2017-01-07 | Human | Jiangxi | 2017/1/7 |
| A/Jiangxi/10666/2017-01-10 | Human | Jiangxi | 2017/1/10 |
| A/Jiangxi/10667/2017-01-10 | Human | Jiangxi | 2017/1/10 |
| A/Jiangxi/10668/2017-01-07 | Human | Jiangxi | 2017/1/7 |
| A/Jiangxi/10669/2017-01-10 | Human | Jiangxi | 2017/1/10 |
| A/Jiangxi/10670/2017-01-14 | Human | Jiangxi | 2017/1/14 |
| A/Jiangxi/10671/2017-01-19 | Human | Jiangxi | 2017/1/19 |
| A/Jiangxi/10672/2017-01-20 | Human | Jiangxi | 2017/1/20 |
| A/Jiangxi/10673/2017-01-22 | Human | Jiangxi | 2017/1/22 |
| A/Jiangxi/10674/2017-01-23 | Human | Jiangxi | 2017/1/23 |
| A/Jiangxi/10675/2017-01-24 | Human | Jiangxi | 2017/1/24 |
| A/Jiangxi/10676/2017-01-24 | Human | Jiangxi | 2017/1/24 |
| A/Jiangxi/10678/2017-01-27 | Human | Jiangxi | 2017/1/27 |
| A/Jiangxi/10679/2017-02-01 | Human | Jiangxi | 2017/2/1 |
| A/Jiangxi/10680/2017-02-02 | Human | Jiangxi | 2017/2/2 |
| A/Jiangxi/10682/2017-02-04 | Human | Jiangxi | 2017/2/4 |
| A/Jiangxi/10683/2017-02-03 | Human | Jiangxi | 2017/2/3 |
| A/Jiangxi/27569/2014-04-21 | Human | Jiangxi | 2014/4/21 |
| A/Jiaxing/20/2014-01-18 | Human | Jiaxing | 2014/1/18 |
| A/Jilin/10117/2014-02-19 | Human | Jilin | 2014/2/19 |
| A/Jilin/24141/2017-04-20 | Human | Jilin | 2017/4/20 |
| A/Kunming/KMCDC-YHY/2017-03-02 | Human | Kunming | 2017/3/2 |
| A/Liaoning/13453/2017-01-27 | Human | Liaoning | 2017/1/27 |
| A/Liaoning/13454/2017-01-27 | Human | Liaoning | 2017/1/27 |
| A/Liaoning/14356/2017-01-27 | Human | Liaoning | 2017/1/27 |
| A/Liaoning/14358/2017-01-27 | Human | Liaoning | 2017/1/27 |
| A/Liaoning/28252/2017-04-19 | Human | Liaoning | 2017/4/19 |
| A/Liaoning/44068/2016-06-13 | Human | Liaoning | 2016/6/13 |
| A/Minhang/S01/2013-02-27 | Human | Minhang | 2013/2/27 |
| A/Nanchang/1/2013-04-24 | Human | Nanchang | 2013/4/24 |
| A/Nanjing/1/2013-03-28 | Human | Nanjing | 2013/3/28 |
| A/Nanjing/2/2013-04-05 | Human | Nanjing | 2013/4/5 |
| A/Nanjing/3/2013-04-19 | Human | Nanjing | 2013/4/19 |
| A/Nanjing/4/2013-04-07 | Human | Nanjing | 2013/4/7 |
| A/Nanjing/5/2013-04-25 | Human | Nanjing | 2013/4/25 |
| A/Nanjing/6/2013-04-11 | Human | Nanjing | 2013/4/11 |
| A/Nanjing/7/2013-04-13 | Human | Nanjing | 2013/4/13 |
| A/Nanjing/f0874/2013-04-09 | Human | Nanjing | 2013/4/9 |
| A/Nanjing/M2/2013-04-26 | Human | Nanjing | 2013/4/26 |
| A/Pigeon/Shanghai/S1069/2013-04-02 | Pigeon | Shanghai | 2013/4/2 |
| A/pigeon/Shanghai/S1421/2013-04-03 | pigeon | Shanghai | 2013/4/3 |
| A/pigeon/Shanghai/S1423/2013-04-03 | pigeon | Shanghai | 2013/4/3 |
| A/pigeon/Wuxi/0405007/2013-04-05 | pigeon | Wuxi | 2013/4/5 |
| A/pigeon/Wuxi/0405007G/2013-04 | pigeon | Wuxi | Apr-13 |
| A/pigeon/Zhejiang/P1/2013-04 | pigeon | Zhejiang | Apr-13 |
| A/pigeon/Zhejiang/P2/2013-04 | pigeon | Zhejiang | Apr-13 |
| A/Qingyuan/GIRD01/2017-01-14 | Human | Qingyuan | 2017/1/14 |
| A/Qingyuan/GIRD1/2017-01-14 | Human | Qingyuan | 2017/1/14 |
| A/Quzhou/1/2015-04-25 | Human | Quzhou | 2015/4/25 |
| A/Quzhou/2/2015-04-23 | Human | Quzhou | 2015/4/23 |
| A/SDYT/01/2016-02-17 | Human | SDYT | 2016/2/17 |
| A/SDYT/02/2016-04-28 | Human | SDYT | 2016/4/28 |
| A/Shaanxi/29410/2017-05-05 | Human | Shaanxi | 2017/5/5 |
| A/Shaanxi/29412/2017-05-05 | Human | Shaanxi | 2017/5/5 |
| A/Shaanxi/32282/2017-05-02 | Human | Shaanxi | 2017/5/2 |
| A/Shaanxi/32284/2017-05-05 | Human | Shaanxi | 2017/5/5 |
| A/Shaanxi/32285/2017-05-13 | Human | Shaanxi | 2017/5/13 |
| A/Shaanxi/32287/2017-06-06 | Human | Shaanxi | 2017/6/6 |
| A/Shandong/0068A/2013-04-28 | Human | Shandong | 2013/4/28 |
| A/Shandong/01/2013-04-21 | Human | Shandong | 2013/4/21 |
| A/Shandong/01/2014-05-22 | Human | Shandong | 2014/5/22 |
| A/Shandong/02/2014-03-04 | Human | Shandong | 2014/3/4 |
| A/Shandong/19419/2017-02-24 | Human | Shandong | 2017/2/24 |
| A/Shandong/21077/2017-01-07 | Human | Shandong | 2017/1/7 |
| A/Shandong-Laiwu/02/2017-01-06 | Human | Shandong-Laiwu | 2017/1/6 |
| A/Shandong-Qingdao/03/2017-01-24 | Human | Shandong-Qingdao | 2017/1/24 |
| A/Shandong-Rizhao/01/2017-01-02 | Human | Shandong-Rizhao | 2017/1/2 |
| A/Shandong-Rizhao/04/2017-02-16 | Human | Shandong-Rizhao | 2017/2/16 |
| A/Shandong-Taian/01/2015-03-07 | Human | Shandong-Taian | 2015/3/7 |
| A/Shanghai/01/2014-01-03 | Human | Shanghai | 2014/1/3 |
| A/Shanghai/02/2013-03-05 | Human | Shanghai | 2013/3/5 |
| A/Shanghai/02619/2014-01-03 | Human | Shanghai | 2014/1/3 |
| A/Shanghai/05/2013-04-02 | Human | Shanghai | 2013/4/2 |
| A/Shanghai/06-A/2013-04-10 | Human | Shanghai | 2013/4/10 |
| A/Shanghai/07/2013-03-18 | Human | Shanghai | 2013/3/18 |
| A/Shanghai/1/2013-02-26 | Human | Shanghai | 2013/2/26 |
| A/Shanghai/1/2013-02-28 | Human | Shanghai | 2013/2/28 |
| A/Shanghai/10/2013-04-09 | Human | Shanghai | 2013/4/9 |
| A/Shanghai/11/2013-04-10 | Human | Shanghai | 2013/4/10 |
| A/Shanghai/12/2013-04-10 | Human | Shanghai | 2013/4/10 |
| A/Shanghai/13/2013-04-10 | Human | Shanghai | 2013/4/10 |
| A/Shanghai/14/2013-04-10 | Human | Shanghai | 2013/4/10 |
| A/Shanghai/15/2013-04-09 | Human | Shanghai | 2013/4/9 |
| A/Shanghai/16/2013-04-10 | Human | Shanghai | 2013/4/10 |
| A/Shanghai/17/2013-04-03 | Human | Shanghai | 2013/4/3 |
| A/Shanghai/2/2013-03-05 | Human | Shanghai | 2013/3/5 |
| A/Shanghai/3/2013-02-27 | Human | Shanghai | 2013/2/27 |
| A/Shanghai/4/2013-03-09 | Human | Shanghai | 2013/3/9 |
| A/Shanghai/4664T/2013-03-05 | Human | Shanghai | 2013/3/5 |
| A/Shanghai/5190T/2013-05 | Human | Shanghai | May-13 |
| A/Shanghai/5190T/2013 | Human | Shanghai | 2013 |
| A/Shanghai/8/2013-04-07 | Human | Shanghai | 2013/4/7 |
| A/Shanghai/9/2013-04-08 | Human | Shanghai | 2013/4/8 |
| A/Shanghai/CN02/2013/04/02 | Human | Shanghai | 2013/4/2 |
| A/Shanghai/ION/2013-04-04 | Human | Shanghai | 2013/4/4 |
| A/Shanghai/JS01/2013/04/03 | Human | Shanghai | 2013/4/3 |
| A/Shanghai/MH01/2013-04-17 | Human | Shanghai | 2013/4/17 |
| A/Shanghai/Mix1/2014/01/03 | Human | Shanghai | 2014/1/3 |
| A/Shanghai/PD-01/2014-01-17 | Human | Shanghai | 2014/1/17 |
| A/Shanghai/PD-02/2014-01-17 | Human | Shanghai | 2014/1/17 |
| A/Shantou/1001/2014-03-10 | Human | Shantou | 2014/3/10 |
| A/Shantou/1002/2014-03-23 | Human | Shantou | 2014/3/23 |
| A/Shantou/1003/2014-04-11_ | Human | Shantou | 2014/4/11 |
| A/Shantou/1004/2014-04-19 | Human | Shantou | 2014/4/19 |
| A/Shanxi/26569/2017-05-14 | Human | Shanxi | 2017/5/14 |
| A/Shanxi/26570/2017-05-14 | Human | Shanxi | 2017/5/14 |
| A/Shanxi/26571/2017-05-14 | Human | Shanxi | 2017/5/14 |
| A/Shaoxing/70/2014-02-03 | Human | Shaoxing | 2014/2/3 |
| A/Shenzhen/SP113/2014-03-12 | Human | Shenzhen | 2014/3/12 |
| A/Shenzhen/SP116/2014-03-15 | Human | Shenzhen | 2014/3/15 |
| A/Shenzhen/SP118/2014-03-18 | Human | Shenzhen | 2014/3/18 |
| A/Shenzhen/SP126/2014-03-23 | Human | Shenzhen | 2014/3/23 |
| A/Shenzhen/SP139/2014-04-02 | Human | Shenzhen | 2014/4/2 |
| A/Shenzhen/SP16/2014-01-18 | Human | Shenzhen | 2014/1/18 |
| A/Shenzhen/SP17/2014-01-20 | Human | Shenzhen | 2014/1/20 |
| A/Shenzhen/SP26/2014-01-20 | Human | Shenzhen | 2014/1/20 |
| A/Shenzhen/SP38/2014-01-22 | Human | Shenzhen | 2014/1/22 |
| A/Shenzhen/SP4/2014-01-16 | Human | Shenzhen | 2014/1/16 |
| A/Shenzhen/SP44/2014-01-23 | Human | Shenzhen | 2014/1/23 |
| A/Shenzhen/SP48/2014-01-23 | Human | Shenzhen | 2014/1/23 |
| A/Shenzhen/SP49/2014-01-25 | Human | Shenzhen | 2014/1/25 |
| A/Shenzhen/SP58/2014-01-25 | Human | Shenzhen | 2014/1/25 |
| A/Shenzhen/SP60/2014-01-30 | Human | Shenzhen | 2014/1/30 |
| A/Shenzhen/SP62/2014-02-05 | Human | Shenzhen | 2014/2/5 |
| A/Shenzhen/SP75/2014-02-15 | Human | Shenzhen | 2014/2/15 |
| A/Shenzhen/SP-W1/2014-01-14 | Human | Shenzhen | 2014/1/14 |
| A/Shenzhen/SP-Z93/2014-01-11 | Human | Shenzhen | 2014/1/11 |
| A/Shenzhen/Th0007/2017-03-12 | Human | Shenzhen | 2017/3/12 |
| A/Shenzhen/Th001/2016-12-31 | Human | Shenzhen | 2016/12/31 |
| A/Shenzhen/Th002/2016_H7N9\|_\|_2016-12-30 | Human | Shenzhen | 2016/12/30 |
| A/Shenzhen/Th003/2017_H7N9\|_\|_2017-01-16 | Human | Shenzhen | 2017/1/16 |
| A/Shenzhen/Th004/2017_H7N9\|_\|_2017-01-20 | Human | Shenzhen | 2017/1/20 |
| A/Shenzhen/Th006/2017_H7N9\|_\|_2017-02-07 | Human | Shenzhen | 2017/2/7 |
| A/Shenzhen/Th008/2017(H7N9)\|_\|_2017-04-27 | Human | Shenzhen | 2017/4/27 |
| A/Sichuan/08606/2017-01-24 | Human | Sichuan | 2017/1/24 |
| A/Sichuan/09679/2017-02-03 | Human | Sichuan | 2017/2/3 |
| A/Sichuan/09680/2017-01-25 | Human | Sichuan | 2017/1/25 |
| A/Sichuan/11020/2017-02-07 | Human | Sichuan | 2017/2/7 |
| A/Sichuan/11021/2017-02-09 | Human | Sichuan | 2017/2/9 |
| A/Sichuan/11022/2017-02-11 | Human | Sichuan | 2017/2/11 |
| A/Sichuan/12218/2017-02-19 | Human | Sichuan | 2017/2/19 |
| A/Sichuan/12219/2017-02-19 | Human | Sichuan | 2017/2/19 |
| A/Sichuan/17267/2017-03-06 | Human | Sichuan | 2017/3/6 |
| A/Sichuan/24974/2017-04-07 | Human | Sichuan | 2017/4/7 |
| A/Sichuan/24976/2017-04-27 | Human | Sichuan | 2017/4/27 |
| A/Sichuan/24977/2017-04-13 | Human | Sichuan | 2017/4/13 |
| A/Sichuan/24980/2017-04-19 | Human | Sichuan | 2017/4/19 |
| A/Sichuan/24981/2017-04-21 | Human | Sichuan | 2017/4/21 |
| A/Sichuan/24982/2017-04-26 | Human | Sichuan | 2017/4/26 |
| A/Sichuan/24983/2017-04-30 | Human | Sichuan | 2017/4/30 |
| A/Sichuan/24984/2017-05-01 | Human | Sichuan | 2017/5/1 |
| A/Sichuan/24985/2017-04-19 | Human | Sichuan | 2017/4/19 |
| A/Sichuan/24986/2017-04-07 | Human | Sichuan | 2017/4/7 |
| A/Sichuan/24987/2017-04-22 | Human | Sichuan | 2017/4/22 |
| A/Sichuan/24989/2017-04-30 | Human | Sichuan | 2017/4/30 |
| A/Sichuan/27180/2017-05-04 | Human | Sichuan | 2017/5/4 |
| A/Sichuan/27181/2017-05-06 | Human | Sichuan | 2017/5/6 |
| A/Sichuan/27182/2017-05-07 | Human | Sichuan | 2017/5/7 |
| A/Sichuan/27183/2017-05-19 | Human | Sichuan | 2017/5/19 |
| A/Sichuan/27184/2017-05-15 | Human | Sichuan | 2017/5/15 |
| A/Sichuan/27872/2017-05-24 | Human | Sichuan | 2017/5/24 |
| A/Sichuan/27873/2017-05-22 | Human | Sichuan | 2017/5/22 |
| A/Sichuan/29763/2017-06-16 | Human | Sichuan | 2017/6/16 |
| A/Sichuan/29764/2017-06-17 | Human | Sichuan | 2017/6/17 |
| A/Sichuan/29765/2017-05-31 | Human | Sichuan | 2017/5/31 |
| A/silkie_chicken/Dongguan/1264/2014-02-21 | silkie_chicken | Dongguan | 2014/2/21 |
| A/silkie_chicken/Dongguan/1268/2014-02-21 | silkie_chicken | Dongguan | 2014/2/21 |
| A/silkie_chicken/Dongguan/1271/2014-02-21 | silkie_chicken | Dongguan | 2014/2/21 |
| A/silkie_chicken/Dongguan/1274/2014-02-21 | silkie_chicken | Dongguan | 2014/2/21 |
| A/silkie_chicken/Dongguan/1448/2014-02-21 | silkie_chicken | Dongguan | 2014/2/21 |
| A/silkie_chicken/Dongguan/1450/2014-02-21 | silkie_chicken | Dongguan | 2014/2/21 |
| A/silkie_chicken/Dongguan/1451/2014-02-21 | silkie_chicken | Dongguan | 2014/2/21 |
| A/silkie_chicken/Dongguan/1516/2014-02-21 | silkie_chicken | Dongguan | 2014/2/21 |
| A/silkie_chicken/Dongguan/1519/2014-02-21 | silkie_chicken | Dongguan | 2014/2/21 |
| A/silkie_chicken/Dongguan/157/2014-02-20 | silkie_chicken | Dongguan | 2014/2/20 |
| A/silkie_chicken/Dongguan/1641/2014-02-21 | silkie_chicken | Dongguan | 2014/2/21 |
| A/silkie_chicken/Dongguan/3049/2013-12-18 | silkie_chicken | Dongguan | 2013/12/18 |
| A/silkie_chicken/Dongguan/3166/2013-12-18 | silkie_chicken | Dongguan | 2013/12/18 |
| A/silkie_chicken/Dongguan/3275/2013-12-18 | silkie_chicken | Dongguan | 2013/12/18 |
| A/silkie_chicken/Dongguan/3281/2013-12-18 | silkie_chicken | Dongguan | 2013/12/18 |
| A/silkie_chicken/Dongguan/3284/2013-12-18 | silkie_chicken | Dongguan | 2013/12/18 |
| A/silkie_chicken/Dongguan/3520/2013-12-19 | silkie_chicken | Dongguan | 2013/12/19 |
| A/silkie_chicken/Dongguan/3522/2013-12-19 | silkie_chicken | Dongguan | 2013/12/19 |
| A/silkie_chicken/Dongguan/3525/2013-12-19 | silkie_chicken | Dongguan | 2013/12/19 |
| A/silkie_chicken/Dongguan/3526/2013-12-19 | silkie_chicken | Dongguan | 2013/12/19 |
| A/silkie_chicken/Dongguan/3528/2013-12-19 | silkie_chicken | Dongguan | 2013/12/19 |
| A/silkie_chicken/Dongguan/3605/2013-12-19 | silkie_chicken | Dongguan | 2013/12/19 |
| A/silkie_chicken/Dongguan/3606/2013-12-19_ | silkie_chicken | Dongguan | 2013-12-19_ |
| A/silkie_chicken/Dongguan/3980/2013-12-19 | silkie_chicken | Dongguan | 2013/12/19 |
| A/silkie_chicken/Dongguan/3990/2013-12-19 | silkie_chicken | Dongguan | 2013/12/19 |
| A/silkie_chicken/Dongguan/4126/2013-12-19 | silkie_chicken | Dongguan | 2013/12/19 |
| A/silkie_chicken/Dongguan/4127/2013-12-19 | silkie_chicken | Dongguan | 2013/12/19 |
| A/silkie_chicken/Dongguan/4129/2013-12-19 | silkie_chicken | Dongguan | 2013/12/19 |
| A/silkie_chicken/Dongguan/523/2014-02-20 | silkie_chicken | Dongguan | 2014/2/20 |
| A/silkie_chicken/Dongguan/635/2014-02-20 | silkie_chicken | Dongguan | 2014/2/20 |
| A/silkie_chicken/Dongguan/656/2014-02-20 | silkie_chicken | Dongguan | 2014/2/20 |
| A/silkie_chicken/Dongguan/953/2014-02-21 | silkie_chicken | Dongguan | 2014/2/21 |
| A/silkie_chicken/Dongguan/963/2014-02-21 | silkie_chicken | Dongguan | 2014/2/21 |
| A/silkie_chicken/Dongguan/967/2014-02-21 | silkie_chicken | Dongguan | 2014/2/21 |
| A/silkie_chicken/Dongguan/969/2014-02-21 | silkie_chicken | Dongguan | 2014/2/21 |
| A/silkie_chicken/Dongguan/979/2014-02-21 | silkie_chicken | Dongguan | 2014/2/21 |
| A/silkie_chicken/Dongguan/981/2014-02-21 | silkie_chicken | Dongguan | 2014/2/21 |
| A/silkie_chicken/Dongguan/986/2014-02-21 | silkie_chicken | Dongguan | 2014/2/21 |
| A/silkie_chicken/Dongguan/988/2014-02-21 | silkie_chicken | Dongguan | 2014/2/21 |
| A/silkie_chicken/Dongguan/991/2014-02-21 | silkie_chicken | Dongguan | 2014/2/21 |
| A/silkie_chicken/Dongguan/997/2014-02-21 | silkie_chicken | Dongguan | 2014/2/21 |
| A/silkie_chicken/Huzhou/4213/2013-10-24 | silkie_chicken | Huzhou | 2013/10/24 |
| A/silkie_chicken/Jiangxi/9469/2014-02-16 | silkie_chicken | Jiangxi | 2014/2/16 |
| A/silkie_chicken/Jiangxi/9472/2014-02-16 | silkie_chicken | Jiangxi | 2014/2/16 |
| A/silkie_chicken/Jiangxi/9476/2014-02-16 | silkie_chicken | Jiangxi | 2014/2/16 |
| A/silkie_chicken/Shantou/1406/2014-02-25 | silkie_chicken | Shantou | 2014/2/25 |
| A/silkie_chicken/Shantou/2050/2014-03-25 | silkie_chicken | Shantou | 2014/3/25 |
| A/silkie_chicken/Shantou/2054/2014-03-25 | silkie_chicken | Shantou | 2014/3/25 |
| A/silkie_chicken/Shantou/2056/2014-03-25 | silkie_chicken | Shantou | 2014/3/25 |
| A/silkie_chicken/Shaoxing/5130/2013-10-28 | silkie_chicken | Shaoxing | 2013/10/28 |
| A/silkie_chicken/Shaoxing/5235/2013-10-28 | silkie_chicken | Shaoxing | 2013/10/28 |
| A/silkie_chicken/Shenzhen/2134/2013-12-13 | silkie_chicken | Shenzhen | 2013/12/13 |
| A/silkie_chicken/Shenzhen/2139/2013-12-13 | silkie_chicken | Shenzhen | 2013/12/13 |
| A/silkie_chicken/Shenzhen/3781/2013-12-19 | silkie_chicken | Shenzhen | 2013/12/19 |
| A/silkie_chicken/Shenzhen/3782/2013-12-19 | silkie_chicken | Shenzhen | 2013/12/19 |
| A/silkie_chicken/Shenzhen/918/2013-12-09 | silkie_chicken | Shenzhen | 2013/12/9 |
| A/silkie_chicken/Shenzhen/919/2013-12-09 | silkie_chicken | Shenzhen | 2013/12/9 |
| A/silky_chicken/Hong_Kong/1772-3/2014-01-27 | silky_chicken | Hong_Kong | 2014/1/27 |
| A/Suzhou/_78/2016-12-26 | Human | Suzhou | 2016/12/26 |
| A/Suzhou/_79/2016-12-27 | Human | Suzhou | 2016/12/27 |
| A/Suzhou/3/2013-04-09 | Human | Suzhou | 2013/4/9 |
| A/Suzhou/467/2016-10-26 | Human | Suzhou | 2016/10/26 |
| A/Suzhou/5/2013-04-12 | Human | Suzhou | 2013/4/12 |
| A/Suzhou/51/2016-12-19 | Human | Suzhou | 2016/12/19 |
| A/Suzhou/54/2016-11-25 | Human | Suzhou | 2016/11/25 |
| A/Suzhou/56/2016-12-02 | Human | Suzhou | 2016/12/2 |
| A/Suzhou/57/2016-12-06 | Human | Suzhou | 2016/12/6 |
| A/Suzhou/58/2016-12-10 | Human | Suzhou | 2016/12/10 |
| A/Suzhou/59/2016-12-12 | Human | Suzhou | 2016/12/12 |
| A/Suzhou/60/2016-12-13 | Human | Suzhou | 2016/12/13 |
| A/Suzhou/63/2016-11-15 | Human | Suzhou | 2016/11/15 |
| A/Suzhou/65/2016-12-09 | Human | Suzhou | 2016/12/9 |
| A/Suzhou/67/2016-12-16 | Human | Suzhou | 2016/12/16 |
| A/Suzhou/67/2016-12-16 | Human | Suzhou | 2016/12/16 |
| A/Suzhou/73/2016-12-20 | Human | Suzhou | 2016/12/20 |
| A/Suzhou/73/2016-12-20 | Human | Suzhou | 2016/12/20 |
| A/Suzhou/74/2016-12-19 | Human | Suzhou | 2016/12/19 |
| A/Suzhou/74/2016-12-19 | Human | Suzhou | 2016/12/19 |
| A/Suzhou/78/2016-12-26 | Human | Suzhou | 2016/12/26 |
| A/Suzhou/79/2016-12-27 | Human | Suzhou | 2016/12/27 |
| A/Suzhou/80/2016-12-26 | Human | Suzhou | 2016/12/26 |
| A/Suzhou/84/2016-12-28 | Human | Suzhou | 2016/12/28 |
| A/Suzhou/86/2016-12-29 | Human | Suzhou | 2016/12/29 |
| A/Suzhou/87/2016-12-30 | Human | Suzhou | 2016/12/30 |
| A/Suzhou/88/2016-12-31 | Human | Suzhou | 2016/12/31 |
| A/Suzhou/90/2017-01-02 | Human | Suzhou | 2017/1/2 |
| A/Suzhou/91/2017-01-02 | Human | Suzhou | 2017/1/2 |
| A/Suzhou/92/2017-01-02 | Human | Suzhou | 2017/1/2 |
| A/Suzhou/93/2017-01-03 | Human | Suzhou | 2017/1/3 |
| A/Suzhou/95/2017-01-04 | Human | Suzhou | 2017/1/4 |
| A/Suzhou/96/2017-01-04 | Human | Suzhou | 2017/1/4 |
| A/Taiwan/01/2013-04-24 | Human | Taiwan | 2013/4/24 |
| A/Taiwan/1/2013-04-24 | Human | Taiwan | 2013/4/24 |
| A/Taiwan/1/2013 | Human | Taiwan | 2013 |
| A/Taiwan/1/2014-04-21 | Human | Taiwan | 2014/4/21 |
| A/Taiwan/1/2017-02-03 | Human | Taiwan | 2017/2/3 |
| A/Taiwan/2/2014-04-24 | Human | Taiwan | 2014/4/24 |
| A/Taiwan/3/2013-12-27 | Human | Taiwan | 2013/12/27 |
| A/Taiwan/4-CGMH1/2014-04-24 | Human | Taiwan | 2014/4/24 |
| A/Taiwan/4-CGMH2/2014-04-24 | Human | Taiwan | 2014/4/24 |
| A/Tianjin/22163/2017-04-13 | Human | Tianjin | 2017/4/13 |
| A/Tianjin/22164/2017-04-13 | Human | Tianjin | 2017/4/13 |
| A/Tianjin/31940/2017-06-20 | Human | Tianjin | 2017/6/20 |
| A/Tianjin/43917/2016-06-12 | Human | Tianjin | 2016/6/12 |
| A/tree_sparrow/Shanghai/01/2013-05-09 | tree_sparrow | Shanghai | 2013/5/9 |
| A/wild_pigeon/Jiangsu/SD001/2013-04-17 | wild_pigeon | Jiangsu | 2013/4/17 |
| A/Wuxi/1/2013-03-31 | Human | Wuxi | 2013/3/31 |
| A/Wuxi/2/2013-03-31 | Human | Wuxi | 2013/3/31 |
| A/Wuxi/3/2013-04-07 | Human | Wuxi | 2013/4/7 |
| A/Wuxi/4/2013-04-07 | Human | Wuxi | 2013/4/7 |
| A/Xinjiang/04062/2018-01-10 | Human | Xinjiang | 2018/1/10 |
| A/Xinjiang/05845/2015-01-08 | Human | Xinjiang | 2015/1/8 |
| A/Xinjiang/05914/2015-01-08 | Human | Xinjiang | 2015/1/8 |
| A/Xinjiang/05915/2014-12-25 | Human | Xinjiang | 2014/12/25 |
| A/Xinjiang/05916/2014-12-25 | Human | Xinjiang | 2014/12/25 |
| A/Xinjiang/73030/2014-07-25 | Human | Xinjiang | 2014/7/25 |
| A/Xinjiang/75802/2014-08-15 | Human | Xinjiang | 2014/8/15 |
| A/Xinjiang/98691/2014-11-29 | Human | Xinjiang | 2014/11/29 |
| A/Xinjiang/98692/2014-11-25 | Human | Xinjiang | 2014/11/25 |
| A/XinjiangBintuan/99117/2014-10-25 | Human | XinjiangBintuan | 2014/10/25 |
| A/XinjiangBintuan/99118/2014-11-20 | Human | XinjiangBintuan | 2014/11/20 |
| A/Xuzhou/1/2013-04-25 | Human | Xuzhou | 2013/4/25 |
| A/Yunnan/0129/2017-02-09 | Human | Yunnan | 2017/2/9 |
| A/Yunnan/09678/2017-02-07 | Human | Yunnan | 2017/2/7 |
| A/Yunnan/13500/2017-02-09 | Human | Yunnan | 2017/2/9 |
| A/Yunnan/13501/2017-01-14 | Human | Yunnan | 2017/1/14 |
| A/Yunnan/32291/2017-06-13 | Human | Yunnan | 2017/6/13 |
| A/Yunnan/32293/2017-06-25 | Human | Yunnan | 2017/6/25 |
| A/Yunnan/32294/2017-06-28 | Human | Yunnan | 2017/6/28 |
| A/Yunnan/40430/2017-11-29 | Human | Yunnan | 2017/11/29 |
| A/Yunnan/YN001/2017_H7N9\|_\|_2017-02-07 | Human | Yunnan | 2017/2/7 |
| A/Yunnan/YN002/2017_H7N9\|_\|_2017-02-09 | Human | Yunnan | 2017/2/9 |
| A/Zhejiang/01/2013-03-25 | Human | Zhejiang | 2013/3/25 |
| A/Zhejiang/02/2013-04-03 | Human | Zhejiang | 2013/4/3 |
| A/Zhejiang/07800/2014-01-23 | Human | Zhejiang | 2014/1/23 |
| A/Zhejiang/07801/2014-01-23 | Human | Zhejiang | 2014/1/23 |
| A/Zhejiang/07802/2014-01-20 | Human | Zhejiang | 2014/1/20 |
| A/Zhejiang/07803/2014-01-23 | Human | Zhejiang | 2014/1/23 |
| A/Zhejiang/07807/2014-01-19 | Human | Zhejiang | 2014/1/19 |
| A/Zhejiang/1/2013-03-24 | Human | Zhejiang | 2013/3/24 |
| A/Zhejiang/1/2014-01-03 | Human | Zhejiang | 2014/1/3 |
| A/Zhejiang/1/2015-01-13 | Human | Zhejiang | 2015/1/13 |
| A/Zhejiang/1/2016-03-07 | Human | Zhejiang | 2016/3/7 |
| A/Zhejiang/1/2017-01-07 | Human | Zhejiang | 2017/1/7 |
| A/Zhejiang/10/2015-02-12 | Human | Zhejiang | 2015/2/12 |
| A/Zhejiang/10/2016-12-18 | Human | Zhejiang | 2016/12/18 |
| A/Zhejiang/11/2015-03-03 | Human | Zhejiang | 2015/3/3 |
| A/Zhejiang/11/2016-12-21 | Human | Zhejiang | 2016/12/21 |
| A/Zhejiang/12/2016-12-25 | Human | Zhejiang | 2016/12/25 |
| A/Zhejiang/13/2014-01-27 | Human | Zhejiang | 2014/1/27 |
| A/Zhejiang/13/2016-12-24 | Human | Zhejiang | 2016/12/24 |
| A/Zhejiang/14/2014-01-14 | Human | Zhejiang | 2014/1/14 |
| A/Zhejiang/14/2016-12-23 | Human | Zhejiang | 2016/12/23 |
| A/Zhejiang/15/2014-01-25 | Human | Zhejiang | 2014/1/25 |
| A/Zhejiang/15/2016-12-21 | Human | Zhejiang | 2016/12/21 |
| A/Zhejiang/16/2014-02-03 | Human | Zhejiang | 2014/2/3 |
| A/Zhejiang/16/2016-12-21 | Human | Zhejiang | 2016/12/21 |
| A/Zhejiang/17/2014-11-25 | Human | Zhejiang | 2014/11/25 |
| A/Zhejiang/17/2016-12-31 | Human | Zhejiang | 2016/12/31 |
| A/Zhejiang/18/2016-12-27 | Human | Zhejiang | 2016/12/27 |
| A/Zhejiang/19/2014-01-23 | Human | Zhejiang | 2014/1/23 |
| A/Zhejiang/19/2016-12-28 | Human | Zhejiang | 2016/12/28 |
| A/Zhejiang/2/2013-04-03 | Human | Zhejiang | 2013/4/3 |
| A/Zhejiang/2/2014-01-09 | Human | Zhejiang | 2014/1/9 |
| A/Zhejiang/2/2017-01-04 | Human | Zhejiang | 2017/1/4 |
| A/Zhejiang/20/2013-04-24 | Human | Zhejiang | 2013/4/24 |
| A/Zhejiang/20/2014-02-03 | Human | Zhejiang | 2014/2/3 |
| A/Zhejiang/20/2016-12-30 | Human | Zhejiang | 2016/12/30 |
| A/Zhejiang/21/2014-02-10 | Human | Zhejiang | 2014/2/10 |
| A/Zhejiang/21/2017-01-04 | Human | Zhejiang | 2017/1/4 |
| A/Zhejiang/22/2013-10-14 | Human | Zhejiang | 2013/10/14 |
| A/Zhejiang/22/2014-02-08 | Human | Zhejiang | 2014/2/8 |
| A/Zhejiang/22/2017-01-08 | Human | Zhejiang | 2017/1/8 |
| A/Zhejiang/23/2014-02-09 | Human | Zhejiang | 2014/2/9 |
| A/Zhejiang/23/2017-01-13 | Human | Zhejiang | 2017/1/13 |
| A/Zhejiang/24/2014-01-24 | Human | Zhejiang | 2014/1/24 |
| A/Zhejiang/24/2017-01-05 | Human | Zhejiang | 2017/1/5 |
| A/Zhejiang/25/2017-01-08 | Human | Zhejiang | 2017/1/8 |
| A/Zhejiang/26/2017-01-18 | Human | Zhejiang | 2017/1/18 |
| A/Zhejiang/27/2017-01-13 | Human | Zhejiang | 2017/1/13 |
| A/Zhejiang/28/2017-01-13 | Human | Zhejiang | 2017/1/13 |
| A/Zhejiang/29/2017-01-08 | Human | Zhejiang | 2017/1/8 |
| A/Zhejiang/3/2014-01-09 | Human | Zhejiang | 2014/1/9 |
| A/Zhejiang/3/2015-01-19 | Human | Zhejiang | 2015/1/19 |
| A/Zhejiang/3/2017-01-01 | Human | Zhejiang | 2017/1/1 |
| A/Zhejiang/30/2017-01-16 | Human | Zhejiang | 2017/1/16 |
| A/Zhejiang/32/2013-04 | Human | Zhejiang | Apr-13 |
| A/Zhejiang/32/2017-02-06 | Human | Zhejiang | 2017/2/6 |
| A/Zhejiang/33/2014-11-27 | Human | Zhejiang | 2014/11/27 |
| A/Zhejiang/33/2017-02-06 | Human | Zhejiang | 2017/2/6 |
| A/Zhejiang/34/2014-12-25 | Human | Zhejiang | 2014/12/25 |
| A/Zhejiang/34/2017-02-06 | Human | Zhejiang | 2017/2/6 |
| A/Zhejiang/35/2014-12-26 | Human | Zhejiang | 2014/12/26 |
| A/Zhejiang/35/2017-02-04 | Human | Zhejiang | 2017/2/4 |
| A/Zhejiang/36/2014-12-28 | Human | Zhejiang | 2014/12/28 |
| A/Zhejiang/36/2017-02-04 | Human | Zhejiang | 2017/2/4 |
| A/Zhejiang/37/2017-01-24 | Human | Zhejiang | 2017/1/24 |
| A/Zhejiang/38/2017-01-05 | Human | Zhejiang | 2017/1/5 |
| A/Zhejiang/4/2014-01-11 | Human | Zhejiang | 2014/1/11 |
| A/Zhejiang/4/2015-01-23 | Human | Zhejiang | 2015/1/23 |
| A/Zhejiang/4/2017-01-05 | Human | Zhejiang | 2017/1/5 |
| A/Zhejiang/5/2014-01-08 | Human | Zhejiang | 2014/1/8 |
| A/Zhejiang/5/2015-01-26 | Human | Zhejiang | 2015/1/26 |
| A/Zhejiang/5/2017-01-02 | Human | Zhejiang | 2017/1/2 |
| A/Zhejiang/6/2014-01-15 | Human | Zhejiang | 2014/1/15 |
| A/Zhejiang/6/2015-01-28 | Human | Zhejiang | 2015/1/28 |
| A/Zhejiang/6/2016-10-07 | Human | Zhejiang | 2016/10/7 |
| A/Zhejiang/6/2017-01-05 | Human | Zhejiang | 2017/1/5 |
| A/Zhejiang/7/2014-02-24 | Human | Zhejiang | 2014/2/24 |
| A/Zhejiang/7/2015-02-04 | Human | Zhejiang | 2015/2/4 |
| A/Zhejiang/7/2016-12-05 | Human | Zhejiang | 2016/12/5 |
| A/Zhejiang/8/2014-02-27 | Human | Zhejiang | 2014/2/27 |
| A/Zhejiang/8/2015-02-02 | Human | Zhejiang | 2015/2/2 |
| A/Zhejiang/8/2016-12-11 | Human | Zhejiang | 2016/12/11 |
| A/Zhejiang/9/2015-02-25 | Human | Zhejiang | 2015/2/25 |
| A/Zhejiang/9/2016-12-12 | Human | Zhejiang | 2016/12/12 |
| A/Zhejiang/DTID-ZJU01/2013/04/03 | Human | Zhejiang | 2013/4/3 |
| A/Zhejiang/DTID-ZJU01/2013-04-03 | Human | Zhejiang | 2013/4/3 |
| A/Zhejiang/DTID-ZJU02/2013/04 | Human | Zhejiang | Apr-13 |
| A/Zhejiang/HZ1/2013-04 | Human | Zhejiang | Apr-13 |
| A/Zhejiang/LS01/2014-02-08 | Human | Zhejiang | 2014/2/8 |
| A/Zhenjiang/1/2013-04-07 | Human | Zhenjiang | 2013/4/7 |
